# High-throughput matrix screening identifies synergistic and antagonistic antimalarial drug combinations

**DOI:** 10.1038/srep13891

**Published:** 2015-09-25

**Authors:** Bryan T. Mott, Richard T. Eastman, Rajarshi Guha, Katy S. Sherlach, Amila Siriwardana, Paul Shinn, Crystal McKnight, Sam Michael, Norinne Lacerda-Queiroz, Paresma R. Patel, Pwint Khine, Hongmao Sun, Monica Kasbekar, Nima Aghdam, Shaun D. Fontaine, Dongbo Liu, Tim Mierzwa, Lesley A. Mathews-Griner, Marc Ferrer, Adam R. Renslo, James Inglese, Jing Yuan, Paul D. Roepe, Xin-zhuan Su, Craig J. Thomas

**Affiliations:** 1 Division of Preclinical Innovation, National Center for Advancing Translational Sciences, National Institutes of Health, Rockville, MD; 2 Laboratory of Malaria and Vector Research, National Institute of Allergy and Infectious Diseases, National Institutes of Health, Bethesda, MD; 3 Department of Chemistry, Georgetown University, 37th and O St., NW, Washington, DC; 4 Department of Biochemistry, Cellular and Molecular Biology and Center for Infectious Diseases, Georgetown University, 37th and O St., NW, Washington, DC; 5 Department of Pharmaceutical Chemistry, Small Molecule Discovery Center, University of California, San Francisco, CA; 6 National Human Genome Research Institute, National Institutes of Health, Bethesda, MD.

## Abstract

Drug resistance in *Plasmodium* parasites is a constant threat. Novel therapeutics, especially new drug combinations, must be identified at a faster rate. In response to the urgent need for new antimalarial drug combinations we screened a large collection of approved and investigational drugs, tested 13,910 drug pairs, and identified many promising antimalarial drug combinations. The activity of known antimalarial drug regimens was confirmed and a myriad of new classes of positively interacting drug pairings were discovered. Network and clustering analyses reinforced established mechanistic relationships for known drug combinations and identified several novel mechanistic hypotheses. From eleven screens comprising >4,600 combinations per parasite strain (including duplicates) we further investigated interactions between approved antimalarials, calcium homeostasis modulators, and inhibitors of phosphatidylinositide 3-kinases (PI3K) and the mammalian target of rapamycin (mTOR). These studies highlight important targets and pathways and provide promising leads for clinically actionable antimalarial therapy.

Current antimalarial treatments rely on drug combinations as recommended by the World Health Organization^1^. Former standards of care such as chloroquine (CQ) and sulfadoxine-pyrimethamine (SP) have been significantly compromised due to drug resistance, leading to adoption of artemisinin combination therapies (ACTs)^2^,^3^. However, most ACTs were found empirically without full validation of drug-drug interactions or mode of action (MOA) and therefore may not represent ideal combinations. For example, partner drugs such as mefloquine (MFQ) or lumefantrine (LUM) appear to act on pathways similar to those of artemisinin-derived drugs and mutations that modulate susceptibility to one drug may also alter effectiveness of the other, leading to increased tolerance to both compounds^4^,^5^,^6^. Disturbingly, parasites exhibiting reduced clearance following ACT treatment have begun to emerge, indicating that new drug combinations are desperately needed^7^,^8^,^9^. Ideal partner drugs would have compatible pharmacokinetics and pharmacodynamics, MOAs that do not promote concurrent resistance, efficacy against existing drug-resistant parasites, and no toxicity. Developing effective, long lasting drug combinations requires evaluation of large numbers of known and candidate antimalarials. While large-scale single agent screens have identified novel antimalarials, there remains a need for an assessment of new antimalarial drug combinations^10^,^11^. We therefore performed high-throughput combination screens on compounds with diverse MOAs to identify multiple classes of compounds that interact favorably against *P. falciparum*.

## Results

### Iterative combination screens result in new antimalarial combinations

We first evaluated 2317 single agents (Supplementary Table 1), including known antimalarials, approved and investigational drugs and mechanistically annotated small molecules against three *P. falciparum* strains (3D7, HB3 and Dd2) (Summary AID: 743367)^5^. The activities of many pharmacologically diverse agents were confirmed including alvespimycin (human HSP90 inhibitor), propafenone (ion channel modulator) and carfilzomib (human proteasome inhibitor) (Supplementary Fig. 1)^12^,^13^,^14^. Other notable findings were the potent activities associated with small molecules targeting human phosphatidylinositide 3-kinases (hPI3K) including GSK-2126458 and NVP-BGT226. We next performed eleven iterative combination screens, with compounds selected from the single agent screen based on potency, mechanistic interest and clinical status (Supplementary Table 2 and 3)^15^. The progression of each subsequent screen incorporated lessons learned and often expanded upon drugs from similar mechanistic classes to further inform on potential mechanistic interactions. Compounds were plated in either 6 × 6 (with 1:3 dilutions) or 10 × 10 (with 1:2 dilutions) dose response matrices. In total, these screens tested 13,910 combinations (including duplicates across screens) and 728,216 data points (for all three parasite lines); all data are accessible via a web-based visualization tool (https://tripod.nih.gov/matrix-client/). As an example, the penultimate screen was the pairwise evaluation of 56 select agents yielding 1540 unique combinations. A comprehensive set of 240 combinations of interest was further assessed in duplicate against two individual cultures for each of the three parasite strains (Supplementary Table 2 and 3). After removing assays that failed to meet our QC criteria (see SI), we applied several combination response metrics to prioritize agents that could be explored as potential antimalarial combination therapies.

**Figure 1:**
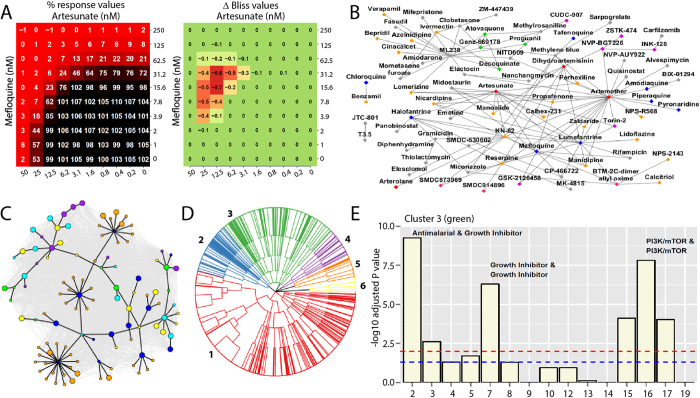
Single agent and combination analysis of a large collection of approved and investigational drugs for antimalarial activity. (**A**) Examples of response profiles (10 × 10 plots) for Artesunate (AS) + Mefloquine (MFQ). Percent response values represent normalized growth, relative to controls based on SybrGreen fluorescence intensities. (**B**) Interaction plot of combinations passing a defined threshold (DBSumNeg <−3; reflecting synergy) against the *Plasmodium falciparum* 3D7 strain (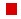 = Endoperoxides; 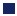 = HLF, LUM, MFQ, CQ, TFQ; 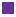 = hPI3K/mTOR; 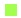 = mitochondrial/DHODH; 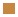 = ion channel modulator; 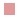 = hybrid mechanism; 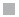 = other). Vector lengths do not reflect the strength or weakness of the interaction. Additional network plots reflecting antagonistic outcomes are found in the SI. Enlarged versions of this figure can be found online at http://tripod.nih.gov/pub/malaria-matrix/. (**C**) The interaction network formed from 2134 combinations of 111 single agents is colored grey. Overlaid on top (black edges) is the sub-network of 110 combinations involving all the single agents representing the most synergistic combinations (as measured by the sum of the DBSumNeg metric). (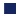 = antimalarial drugs; 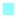 = growth inhibitor; 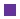 = hPI3K/mTOR; 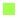 = mitochondrial/DHODH; 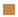 = ion channel modulator; 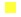 = signaling/transporter inhibitor. Large symbols correspond to the high potency class and small symbols correspond to medium potency class). (**D**) Hierarchical clustering of combination profiles based upon 1) synergy assessment; 2) potency class; 3) mechanism of action (MOA) relationship. The number of clusters (six) was selected based on the largest number of clusters that led to zero or one cluster that was not enriched in any MOA combination (at the 0.01 level). (**E**) A summary of MOA combinations enriched in cluster 3 (green), relative to the entire dataset (see SI for full list of code definitions). Enrichment was tested using Fisher’s exact test with p-values adjusted using the Benjamini-Hochberg method, and plotted as –log_10_ p-value. The blue and red dashed lines correspond to p = 0.05 and p = 0.01 respectively. While six MOA combinations were significant at the 0.01 level, we annotated the top three. See SI for full figure.

The approved and investigational drugs included a collection of antimalarials including dihydroartemisinin (DHA), artemether (ATM), artesunate (AS), CQ, MFQ, amodiaquine (AQ) and piperaquine (PPQ) as well as drugs designated for numerous and diverse indications. To better understand the standard-of-care for treating malaria infections we analyzed the currently approved ACTs (ATM-LUM, AS-MFQ, AS-pyronaridine, AS-AQ, DHA-PPQ). Consistent with previous reports both ATM-LUM and AS-MFQ were noted to interact favorably (Fig. 1A, Supplementary Fig. 2)^16^,^17^. Several combinations exceeded the synergy noted for ATM-LUM and AS-MFQ including 13 drug combinations listed in Table 1. Additionally, many hitherto unexplored drug combinations were identified as being synergistic or additive, including combinations of approved antimalarials (ARTs, LUM, MFQ) with ion channel modulators (e.g. nicardipine), novel mitochondrial targeting agents (e.g. ML238), drugs targeting human enzymes and receptors (e.g. BIX-01294, alvespimycin and NVP-BGT226), and agents currently undergoing single agent clinical assessment in malaria trials (tafenoquine) (Table 1, Supplementary Fig. 2). The novel spiroindolone NITD609 was found to possess several interesting combinatorial outcomes (Supplementary Tables S38 and S39). Comparatively, far fewer antagonistic combinations were identified, reflecting potential bias toward synergy as the effort evolved. New combinations found within this effort represent exciting leads for further evaluation as potential antimalarial therapies.

### Network and clustering analyses highlight common interaction themes

To study the nature and potential mechanism of drug-drug interactions uncovered in these screens we constructed interaction networks for the subset of combinations that were judged most synergistic using the Delta Bliss Summary (DBSum) metric (e.g. DBSum <−3 for the 3D7 strain, Fig. 1B; see Supplementary Fig. 3 for similar plots for strains Dd2 and HB3; see SI for cutoff justification). While agents with unique and often unknown MOA can marginalize attempts to derive useful interaction networks, the incorporation of drug class redundancy enhances the likelihood that networks built for agents with common MOAs will reflect true outcomes. These analyses demonstrated that related antimalarials (ATM, AS and DHA, for instance) possess similar interaction networks (Supplementary Fig. 4). Furthermore, interactions involving ART drugs parallel those seen for LUM and halofantrine (HLF), promoting the notion that these drugs possess a degree of overlap in the parasite response network (Supplementary Fig. 5). Similar network analyses also identified potentially antagonistic interactions exhibiting DBSum >3 (Supplementary Fig. 6). Antagonistic combinations were also identified including benzamil-pyronaridine, NITD609-monensin and lumefantrine-nanchangmycin (Supplementary Fig. 6, Supplementary Tables S22, S23, S24 and S39); confirmatory screens and investigation into mechanistic drug-drug interaction are ongoing. We further analyzed a subset of 2,134 high quality combinations using 111 single agents and identified a set of 110 combinations involving all single agents that were judged most synergistic by the DBSum metric (Fig. 1C) (see Supplementary Table S6 for a listing of these combinations and the SI for details). These analyses clearly show the extensive synergy that occurs between antimalarials (blue nodes) and ion channel blockers (orange nodes) and provide a broad interaction database for development of new antimalarial drug combinations.

As we gained an appreciation of mechanistic classes that achieved impressive single agent and combination activity we noted a consistent enrichment of certain pharmacologically related classes of agents. We therefore analyzed a subset of 1760 combinations exploiting three categorical variables; single agent potency class, MOA relationship, and synergy assessment (see SI). Utilizing these variables, a hierarchical clustering identified six clusters that demonstrated enrichment of specific MOA relationships that generally supported the outcomes of each progressing matrix experiment (see SI for full details of the method, cluster selection and enrichment analysis.). For example, Cluster 3 (green in Fig. 1D) is significantly enriched in combinations belonging to six MOA pairs (Fishers exact test, p < 0.01). The top three MOA combinations include antimalarials (ARTs, quinolines, LUM/HLF), growth inhibitors and PI3K/mTOR inhibitors (Fig. 1E). The network and clustering analyses generated mechanistic hypotheses that can be exploited to better understand individual combination results.

### Association profiles suggest targets of interest

The MOA and interaction analyses can provide information for inferring drug target(s). For instance, the novel antimalarial ML238 was found to be highly synergistic with mitochondrial targeting drugs atovaquone and decoquinate, suggesting it may also modify mitochondrial function (Supplementary Fig. 7 and 8). Strong synergy was also noted for ML238+proguanil (a known mitochondrial sensitizer) and ML238+Genz669178 (a PfDHODH inhibitor) further supporting a mitochondrial MOA for this agent (Supplementary Fig. 8). ML238 was, in fact, recently shown to inhibit cytochrome b at a domain distinct from atovaquone^18^.

Among the MOA classes that repetitively clustered together were agents that modify ion homeostasis in combination with the ART class. Indeed the largest enrichment within our hierarchical clustering analysis was the combination of antimalarials (ARTs, quinolines, LUM/HLF) and ion channel modulators [cluster one (red in Fig. 1D)]. Ion homeostasis, particularly calcium homeostasis, plays a significant role in parasite biology and the interaction of ion channel blockers and antimalarial drugs is well documented^19^. Ion channel antagonists such as nicardipine, manidipine (Ca^2+^) and propafenone (Na^+^) were synergistic with the ARTs and quinolines (Fig. 1B, Supplementary Fig. 3–5,9)^14^. Other modulators of ion homeostasis such as the small molecule KN-62 demonstrated strong synergy with many approved antimalarial drugs (Supplementary Fig. 3–5,9; Table S4, S5). KN-62 is an inhibitor of the human Ca^2+^/calmodulin-dependent protein kinase II (CaMKII), which previously has been shown to perturb cytosolic Ca^2+^ flux and disrupt or modulate cell signaling pathways in *Plasmodium*^20^.

Interestingly, there was a notable lack of differential responses based upon which *P. falciparum* strain was tested. Strain dependent potency changes were noted. For instance, a significant loss of CQ potency was noted in the CQ resistant (CQR) Dd2 strain. However, differentially synergistic or additive or antagonistic drug combinations were rarely found. Utilizing a converted Z’ score of <−2 for DBSumNeg and >+2 for DBSumPos values in several matched assays we found that less than 1% of all combinations possessed strain-dependent differences in combination response. For instance, in one of the 6 × 6 assay sets versus 3D7, HB3 and Dd2 (Assay ID’s 1463, 1465 and 1464, respectively) there was only one combination found to possess significant divergence (block 684 representing a combination of antibiotics monesin and clindamycin which possessed a DBSumPos value of 1.17 in 3D7 and a DBSumNeg value of -1.03 in HB3).

### Connectivity between Ca^2+^ homeostasis and mitochondrial fitness in P. falciparum

**Figure 2:**
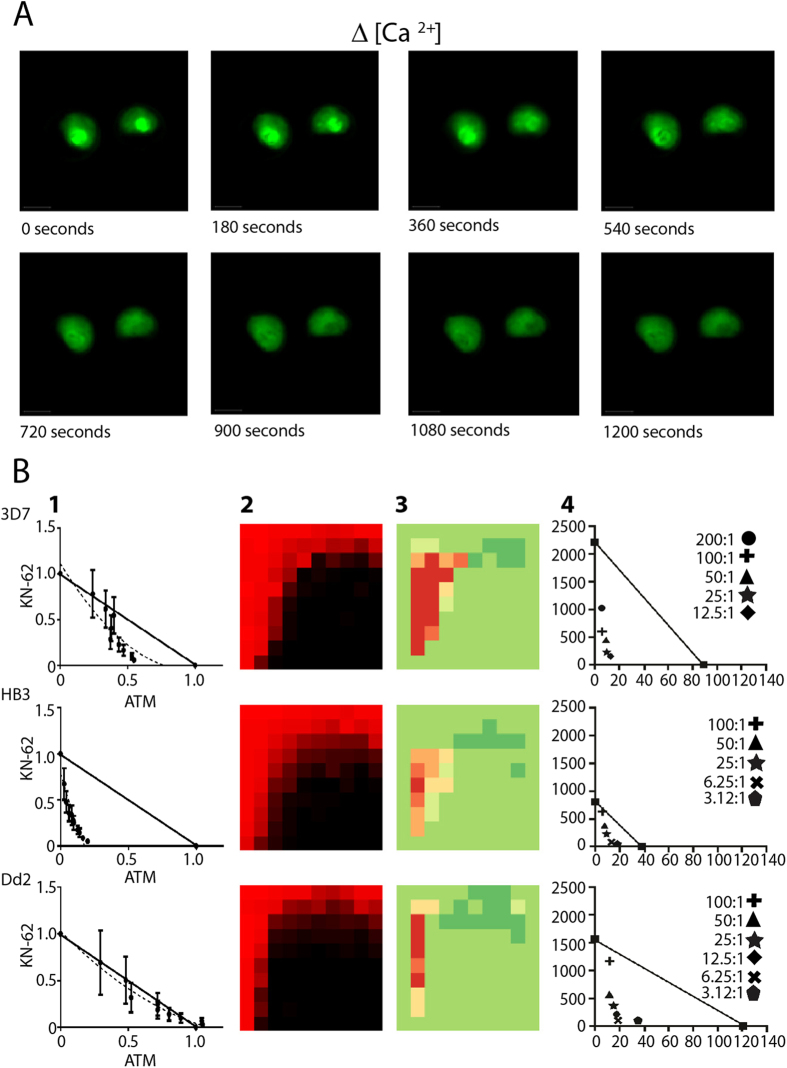
Disruption of calcium homeostasis and alteration of mitochondrial potential for selected drugs and drug pairs. (**A**) Time lapsed capture of calcium dependent Fura −2 fluorescence for two live side-by-side intraerythrocytic strain Dd2 parasites showing rapid loss of digestive vacuole (D.V.) Ca^2+^ (bright green inner circle, top panels) upon perfusion with cytocidal (2 × LD_50_) dose of CQ (see methods). (**B**) Examination of the combination responses of KN-62 and Artemether (ATM) in three parasite lines via an isobologram analysis of the mitochondrial membrane potential as judged by a combination JC1 assay (panel 1), heatmap analysis of the viability combination response (10 × 10 matrix) (panel 2), Delta Bliss analysis (panel 3), and isobolographic analysis of the viability combination response (panel 4).

The calcium channel inhibitor verapamil is known to reverse CQ resistance in *P. falciparum*. Several reports demonstrate that this phenotype is the result of verapamil’s interaction with the *P. falciparum* CQ resistance transporter (PfCRT) altering carrier-mediated drug efflux of CQ^21^,^22^,^23^. At least one study failed to associate the level of CQ efflux with sensitivity in the presence and absence of verapamil and a recent analysis confirmed that diverse PfCRT variants differ in their ability to modulate CQ transporter^24^,^25^. Our studies indicate that the level of synergy found for antimalarials and ion channel modulators is maintained across the CQ sensitive (CQS) 3D7 and HB3 strains and the CQR Dd2 strain (Fig. 2B, Supplementary Fig. 3–5,9). This generalized and consistent level of synergy in both CQR and CQS lines is noteworthy given that the potential contribution of drug transporters might otherwise be theorized to result in differential synergy in these lines. We performed a Tanimoto similarity index analysis for verapamil, reserpine, nicardipine, manidipine, and propafenone which confirmed that with the exception of nicardipine and manidipine the chemical structures of these agents were diverse and unrelated. While structural variation does not rule out comparable interactions with PfCRT or other drug transporters, this result prompted us to examine alternative connectivities between ion transport modulation and antimalarial action.

An examination of calcium homeostasis and mitochondrial membrane polarization, both closely tied to parasite viability, was conducted for potential insight into the mechanism of these combination responses. Utilizing single cell photometry we noted an acute release of Ca^2+^ from the parasite digestive vacuole (DV) with a concomitant increase in cytosolic Ca^2+^ upon treatment with CQ (Fig. 2A, Supplementary Fig. 10, Supplementary Table S7 and S8). A similar increase in cytosolic Ca^2+^ was noted for ATM, LUM, propafenone, nicardipine, reserpine and KN-62 (Supplementary Table S9). Calcium homeostasis is regulated in all cells through several complementary mechanisms including the action of membrane transporters and the use of ER and lysosomal (DV) stores^26^. Mitochondrial Ca^2+^ uptake is also an important regulatory process and pronounced Ca^2+^ uptake can be accompanied by a transient loss of polarization which can be detected experimentally. By monitoring the mitochondrial potential in all three *P. falciparum* strains (3D7, HB3 and Dd2) in response to selected drugs applied singly or in combination we were able to judge which agents altered mitochondrial membrane potential and assess if these actions were synergistic (Fig. 2B, Supplementary Fig. 11). Strikingly, each of the drugs shown to cause an increase in cytosolic Ca^2+^ was found to induce mitochondrial depolarization (Supplementary Fig. 11, Supplementary Table S9). Furthermore, several agents (PIK-93, cinacalcet, elesclomol, GSK-1059615) that did not modify cytosolic Ca^2+^ levels were found to have no effect on mitochondrial polarization (Supplementary Table S9). Examination of drug combination effects on mitochondrial depolarization mirrored the outcomes from our parasite viability screens. For instance, the effect on mitochondrial potential for the combination of ATM and KN-62 was synergistic or additive in each line (Fig. 2A, panel 1) which mirrored the synergistic responses in our studies (Fig. 2B, panel 2, 3 and 4). Similar results were seen for many ARTs and ion channel modulator combinations including the combination of ATM and reserpine (Supplementary Fig. 11). These data suggest a relationship between Ca^2+^ release and mitochondrial depolarization, and further substantiate the connectivity of calcium homeostasis to the actions of multiple antimalarial agents. While these data represent a new element to the debate surrounding the contribution of calcium channel modulation to antimalarial action they do not eliminate the potential contribution of PfCRT or other drug transporters to the synergy noted between antimalarials and ion channel modulators.

### Drugs that influence oxidative stress and autophagy responses combine to kill P. falciparum

Pharmacological induction of oxidative stress or alteration of the parasite response to elevated reactive oxygen species (ROS) is a postulated MOA for several antimalarial drug classes. ARTs are reportedly activated by hemoglobin degradation products, presumably heme or ferrous iron, thereby inducing oxidative stress within the DV^27^. The endoperoxide pharmacophore of ARTs is absolutely required for activity, and several studies have correlated excess ROS levels with activity^27^,^28^. Cross-talk between ROS levels and calcium signaling is well documented, and our analyses showed ROS induction by DHA, ATM and methylene blue (Supplementary Fig. 12)^29^. Additional stress response elements such as heat shock proteins (HSP) are being evaluated as therapeutic targets in *Plasmodium* and various HSP90 inhibitors such as alvespimycin and NVP-AUY922 demonstrated strong single agent activity in our screens (IC_50_ values less than 100 nM, Supplementary Table 1)^13^. Notably, both compounds generally combined favorably with ART drugs (Supplementary Fig. 13).

Recent reports suggest that a regulated autophagy cascade in response to oxidative stress may play a role in *P. falciparum* fitness and response to selected therapies. A quantitative trait loci (QTL) analysis of CQR versus CQS parasite strains correlates increased numbers of *Plasmodium* autophagy-related protein 8 (PfATG8) puncta with drug response and identified candidate drug resistance genes involved in modulating this cascade^30^. Interestingly, in all eukaryotes, the autophagy cascade requires the coordinated function of multiple autophagy-related proteins and the regulatory activities of the PI3K Vps34^31^,^32^. Our single agent screens included numerous investigational drugs targeting human PI3Ks and mTOR (Supplementary Table 1), including the potent mTOR inhibitor torin 2 which was recently reported to have impressive antimalarial activity against the asexual, liver and gametocyte stages of malaria^33^,^34^. Additional examples include advanced clinical candidates NVP-BGT226 (a structural congener of torin 2, Dd2 IC_50_ = 1 nM), GSK-2126458 (Dd2 IC_50_ = 124 nM), INK-128 (Dd2 IC_50_ = 69 nM) and ZSTK-474 (Dd2 IC_50_ = 67 nM) (Supplementary Fig. 1). *Plasmodium* possesses only one PI3K (a class III Vps34 ortholog) and homology between this ortholog and the *Drosophila melanogaster* Vps34 indicates that targeting of PfVps34 by these agents is possible (Supplementary Fig. 14)^35^. There are, nevertheless, other potential targets for these drugs that could explain their strong activity. The combination experiments highlighted an array of interactions between PI3K inhibitors and HSP90 inhibitors, protein synthesis inhibitors, mitochondrial function disruptors, CQ, MFQ, DHA, ATM, AS, and LUM (Supplementary Fig. 15).

**Figure 3:**
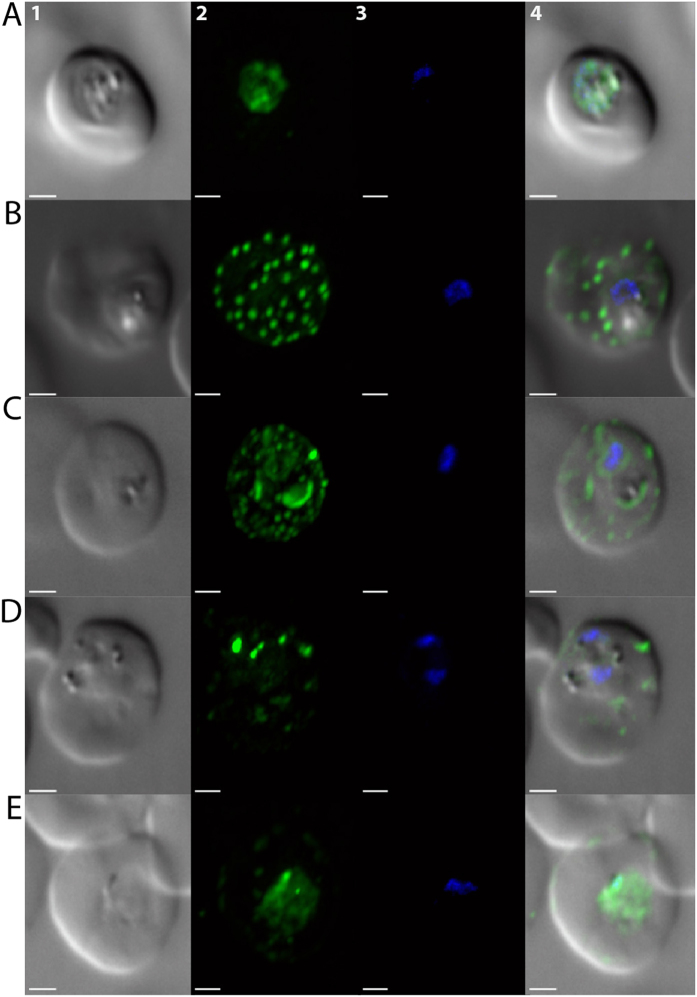
Analysis of autophagosomal body puncta formation and trafficking in response to environmental and/or pharmacological stress. Imaging and quantification of PfAtg8 containing puncta within parasite (the *Plasmodium falciparum* HB3 strain) infected RBC after 6 hour bolus exposure to Artemether (ATM) or ATM combinations: (**A**) ATM at the defined IC_50_ value (23 nM). (**B**) ATM at the defined LD_50_ value (80 nM). (**C**) ATM and Lumefantrine (LUM) at their respectively defined LD_50_ values (80 nM and 323 nM, respectively). (**D**) ATM at the defined LD_50_ value and GSK-2126458 at the defined LD_50_ value (102 μM). (**E**) ATM at the defined LD_50_ value and NVP-BGT226 at the defined LD_50_ value (18 nM). Panel 1: transmittance image. Panel 2: anti-PfAtg8 peptide antibody imaging (ex: 450-490, em: 500 to 550). Panel 3: DAPI nuclear staining (ex: 340–380, em: 450 to 490). Panel 4: a merged image of all three views.

An investigation of the parasite autophagy-related response to selected drug combinations proved insightful. In eukaryotes, PfAtg8 positive vesicle formation is downstream from Vps34. In *P. falciparum*, PfAtg8 in part tracks to apicoplast-targeted vesicles^31^,^32^,^36^. In response to cytocidal CQ treatment PfAtg8 has been shown to traffic in a more radially dispersed pattern along with an autophagosomal complex that includes PfRab7^30^,^32^,^36^. A similar radial distribution of puncta occurs upon treatment with DHA, ATM, AS, and LUM (Fig. 3A,B, Supplementary Fig 16). Similar to CQ, radial distribution of puncta in response to ATM was increased at LD_50_ dose (Fig. 3B) relative to IC_50_ dose (Fig. 3A), and application of Coartem (ATM+LUM) induced an even greater response (Fig. 3C, Supplementary Fig. 16). Interestingly, administration of NVP-BGT226 or GSK-2126458 at their LD_50_ concentrations in combination with ARTs, LUM, AQ and CQ or applied to parasites cultured in starvation conditions did not elicit the same increase in autophagosomal trafficking (Figs 3D, 3E, Supplementary Fig. 16–18). Autophagy is a prosurvival response; however, rapid overstimulation of the autophagy response can lead to cell death. Following administration of two autophagy-inducing agents (e.g. ATM and LUM), parasite fitness may be compromised due to the rapid onset of the autophagy response. Administration of a stimulant and inhibitor (e.g. ATM and NVP-BGT226) may lead to parasite death through blockade of this key survival response. Inhibition of human class I PI3Ks (the targets of NVP-BGT226 and GSK-2126458) and mTOR can have contextually diverse effects on autophagy while inhibition of Vsp34 is generally regarded as a means to mitigate the autophagy response. If these drugs do inhibit PfVps34 it suggests a role for this key regulator protein and PI(3)P in the parasite autophagy response and that targeting this response is deleterious to parasite fitness.

### *In vivo* outcomes highlight combinations of antimalarials and PI3K inhibitors

**Figure 4:**
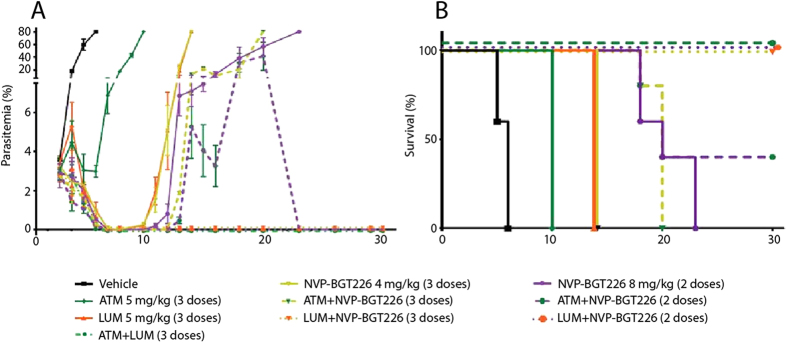
Comparative *in vivo* activities for Artemether (ATM), Lumefantrine (LUM) and NVP-BGT226 as single agents and in combination. *P. berghei* infected BALB/c mice were treated with ATM (5 mg/kg), LUM (5 mg/kg), NVP-BGT226 (4 mg/kg), or NVP-BGT226 (8 mg/kg), or combinations thereof as indicated above. (**A**) Parasitemia values for ATM, LUM and NVP-BGT226 as either single agents or in combination. All treatment was started three days post infection, with doses administered by oral gavage on day 3, 4 and 5 (3 doses) or on days 3 and 4 (2 doses). (**B**) Survival plot for ATM, LUM and NVP-BGT226 as either single agents or in combination. All mice that survived until day 30 (5/5- ATM/LUM (3 doses); 5/5- LUM/NVP-BGT226 (3 doses); 2/5- ATM/NVP-BGT226 (2 doses); 5/5- LUM/NVP-BGT226 (2 doses)) remained negative for parasites through 60 day post infection follow-up.

To assess the clinical potential of selected agents and combinations, we utilized a modified Peter’s 3-day test to confirm the activities of several drugs including propofanone, NVP-BGT226 and GSK-2126458 (Supplementary Fig. 19). Key drug combinations were advanced into a modified Thompson 30-day test where cohorts of 5 animals were infected and treated orally for 2 or 3 days^37^. Single agent cohorts treated with ATM (5 mg/kg), LUM (5 mg/kg), and NVP-BGT226 (4 mg/kg) experienced a rebound of parasitemia reaching 80% between days 5 and 14 (Fig. 4A,B). Cohorts treated three times with the combination of ATM (5 mg/kg) + NVP-BGT226 (4 mg/kg) and a higher single agent dose of NVP-BGT226 (8 mg/kg) administered twice had no detectable parasites by day 7, but eventually succumbed to infection between days 18 and 23 (Fig. 4A,B). Of note, a three dose treatment with LUM (5 mg/kg) + NVP-BGT226 (4 mg/kg) or a two dose treatment of LUM (5 mg/kg) + NVP-BGT226 (8 mg/kg) was curative, as was the standard of care regimen ATM+LUM, remaining parasite free up to the termination of the experiment (day 60). Further, a fraction of the cohort receiving the two dose treatment of ATM (5 mg/kg) + NVP-BGT226 (8 mg/kg) were cured of the infection. The relative success of this combination requires additional study as mice experienced significant weight loss during the 2 and 3 day treatment window. All mice cured of parasites, however, did recover following cessation of treatment (Supplementary Fig. 20). These data suggest structural analogues of NVP-BGT226 that retain antimalarial activity with decreased toxicity would be strong translational candidates.

### Cytocidal and cytostatic potentials in new antimalarial combinations

Translation of drugs and drug combinations requires an appreciation of both IC_50_ and LD_50_ (cytocidal) activities. A recent study of quinoline-based combinations highlights that drug pairs found to be synergistic by a fixed-ratio isobologram methodology using IC_50_ derived values to quantify activity were not necessarily synergistic when LD_50_ values were tested^38^. We therefore examined the LD_50_ values for several agents including ATM, LUM, NVP-BGT226, torin 2, and GSK-2126458 (Supplementary Table 10). Importantly, while LD_50_ doses for quinoline-based drugs are often higher relative to IC_50_ doses (for example CQ; LD_50_ = 250 nM, IC_50_ = 20 nM for the CQS strain HB3, LD_50_ = 15,000 nM, IC_50_ = 200 nM for the CQR strain Dd2) the LD_50_ and IC_50_ values for ARTs and some PI3K targeting drugs are more closely aligned. Further, the *in vivo* ratios of drug combinations are governed by each agent’s pharmacokinetics and change throughout the duration of each agent’s exposure. To assess the translational potential of a given combination, it is necessary to reassess synergy/additivity/antagonism at the estimated *in vivo* exposure concentrations (and ratios) of each agent. For instance, the published single agent exposure profiles for LUM and NVP-BGT226 suggest that these agents will exist at a 500:1 relative ratio (LUM:NVP-BGT226) during their *in vivo* exposure (see SI). To appreciate how each alternate drug:drug ratio will alter their combination profile we analyzed several combinations at ratios determined by their IC_50_, LD_50_ and anticipated *in vivo* concentrations using the Chou-Talalay method. Interestingly, several combinations shown to be additive using IC_50_ data were found to be synergistic or antagonistic when examined at LD_50_ defined ratios (Table 2). Examples include the combinations of ATM+NVP-BGT226 and GSK-2126458 as well as LUM+NVP-BGT226 and GSK-2126458 (Table 2 and Supplementary Table S8). Synergy/antagonism estimations were also noted to be strain dependent. For instance, ATM+NVP-BGT226 combinations at ratios of 1:100, 1:200 and 1:400 were noted to be synergistic in Dd2 but antagonistic in HB3. These results substantiate that synergy, additivity and antagonism are conditional and will depend on many variables including drug concentration, ratio and environmental factors.

## Discussion

Here, we present the results of large-scale combination screens of known antimalarial drugs and approved or investigational drugs shown to possess antimalarial activity and identify many promising drug combinations against *P. falciparum*. This effort provides over 4,600 discreet combinations across three diverse parasite lines and the entirety of this dataset is publically available at https://tripod.nih.gov/matrix-client/. Evaluation of the dataset as a whole can be pursued using multiple techniques. Our own clustering analyses show that distinct MOAs often yield similar combination outcomes and interaction mapping further highlights connectivity between drug classes. These data prompted further evaluation of interesting mechanistic hypotheses. Modulators of calcium homeostasis and endoperoxide-based drugs were found to have profound effects on mitochondrial polarity, which often mirrored the outcomes of viability assays for these drug combinations. Selected mammalian PI3K inhibitors were found to block the parasite autophagy-like response to drug and environmental stress. Many of these mammalian PI3K inhibitors possess impressive cytocidal activity, including NVP-BGT226 which demonstrated activities equal to ART based drugs. *In vivo* analysis of this drug alone and in combination at comparable doses demonstrated equivalent survival and reduction of parasitemia relative to approved standards of care. The response to a single drug or drug combination is a multi-step processes involving target engagement, drug transport, activation and degradation, with possible involvement of other unknown molecular interactions and genetic divergences. Variations in these steps as well as the natural parasite cycles which may alter target candidacy will have significant consequences for drug efficacy. Strain-dependent differences further highlight the complexity of anti-malarial drug discovery efforts and many of the outcomes of this study will require detailed follow-up with these variability’s in mind. The breadth of the combination screen prevents detailed follow-up of every combination within this report. However, the release of the entire dataset provides a public archive that we hope stimulates broader examination of drugs and drug combinations for the treatment of malaria.

## Materials and Methods

### Parasites, parasite culture, quantitative high throughput drug assay and matrix combination screening

The *P. falciparum* parasite lines were previously described and maintained in *in vitro* culture conditions as described^5^. Methods for the SYBR qHTS and calculation of IC_50_ and definition of curve classes have been described^5^,^39^,^40^. Plating of compounds in matrix formation using acoustic droplet ejection and numerical characterization of synergy, additivity and/or antagonism have also been described^15^. All HTS assays were read at 72 hours. Percent response values shown in matrix heat maps represent relative growth as judged by SybrGreen fluorescence intensity values normalized to controls.

### Compound screening collection

The compound screening collection consisted primarily of commercially available molecules in advanced clinical stages, obtained from suppliers such as Tocris Bioscience, Selleck Chemicals, Santa Cruz Biotechnology and Sigma Aldrich (among others). Priority was given to compounds based on known or generally accepted mechanisms of action, clinical status, FDA approval, or novelty in MOA. Several compounds, including NITD609, that were not commercially available were synthesized according to literature procedure^11^,^41^,^42^,^43^.

### QC criteria

The quality control score is a numerical characterization of the quality of a combination that attempts to take into account single agent performance and the presence of noise in the dose combination region of the matrix. It is composed of a number of heuristics, developed by examination of a series of matrix screening runs.

### Additional matrix metrics

In addition to the previously described metrics^15^ we expanded our analysis to include two new quantifiers of synergy – DBSumNeg and DBSumPos. Considering all dose combinations tested, these are defined as the sum of positive deviations from the Bliss model and the sum of the negative deviations from the Bliss model, respectively. In contrast to simply summing all deviations from the Bliss model, these two variables characterize the extent of synergy and antagonism, respectively, within a set of dose combinations.

### Expanded isobologram analyses

Many studies evaluating drug synergies rely upon static values such as isobolographic analyses or combination indices (CI) derived from the multiple drug effect equation developed by Chou and Talalay^44^,^45^,^46^.

### Response network maps

We constructed interaction networks by denoting single agents as nodes and joining two nodes by an undirected edge if those single agents were tested in combination. Subnetworks were investigated in two ways:

Interaction networks were generated and analyzed using Cytoscape v3.0.2 (www.cytoscape.org). All combination screen data (Supplementary Table 3) was initially filtered based on a QC Score equal or less than 5 (See QC Criteria above for description), subsequently all results were manually analyzed to verify 1) 100% ± 10% growth in the untreated control well, 2) At least 25% drug-induced growth inhibition present in assay, and 3) A dose-dependent drug inhibition trend with less than 20% variance not in the trending direction with a tolerance of 3 or less wells in the 6 × 6 matrix and 5 or less in the 10 × 10 matrix assays not within this criteria, as long as the wells not in compliance did not fall along the inhibition edge (i.e. non-tolerant wells did not impede the interpretation of the interaction plot) (Supplementary Table 14). We then analyzed all of the self-cross assays (Supplementary Table 15) that were run to determine the interaction cut-off values for evaluating positive interactions (additive/synergy) and negative interactions (antagonism). The average Delta Bliss Sum Positive (DBSum Pos; suggestive of antagonism) was 2.7 with a STD of 4, and the average Delta Bliss Sum Negative (DBSumNeg; suggestive of additive/synergy) was −2.4 with a STD of 2.2. Based on these criteria we set a conservative cut off for both DBSumPos and DBSumNeg of 3 or −3, respectively that reflected the spectrum of weak to strong negative and positive interactions. Network edges are visual representation of the indicated interaction, they do not indicate the strength of the interaction. Only a single interaction is illustrated for each drug-drug combination, self-loops have been removed. Interaction Networks were generated using these criteria for all three *P. falciparum* lines (3D7, Dd2 and HB3; Supplementary Tables 16–18) for all compounds for both DBSumNeg (Supplementary Tables 19–21) and DBSumPos (Supplementary Tables 22–24) as well as networks with a DBSumNeg of less than -3 for specific compounds (Supplementary Tables 25–38).

Second, rather than considering the 13,910 combinations tested in all, we focused on a subset of 2,134 high quality 10 × 10 combinations as well as smaller subsets selected on the basis of a DBSumNeg threshold. We were interested in identifying the subset of combinations involving all 111 single agents that represented the most synergistic combinations (as measured by DBSumNeg). Given the complete network of 2,134 combinations this minimal subset is equivalent to the minimum spanning tree^47^ (MST) of the whole network (Supplemenatry Fig 21). The MST of a given network is a subnetwork that connects all the nodes such that the sum of the weights of the edges in this subnetwork is smaller than the sum of weights of any other subnetwork that connects all the nodes. Network edges are weighted by the DBSumNeg value, however the length depicted is an artifact of the layout and does not reflect the degree of drug-drug interaction. Since the edges are weighted by DBSumNeg the MST is the subnetwork of all the single agents that minimizes the sum of DBSumNeg. Since more negative values of DBSumNeg correspond to (theoretically) increased synergy, the MST represents the set of combinations that are the most synergistic. Figure 1C displays the MST of the 111 single agents overlaid on the full network (grey) of 2,134 combinations. Supplementary Table 6 lists the 2,134 combinations.

However, the use of DBSumNeg can be problematic since it can be skewed by poor quality or noisy combination responses. Combinations that exhibit large negative values of DBSumNeg can be false positives (i.e., not necessarily synergistic). While the use of the QC score alleviates this to an extent, manual inspection of the most synergistic subset must be performed. This can be seen from Supplementary Figure 22, which redraws the MST but now colors the edges (i.e., combinations) based on a manual inspection of the combination response matrix and subsequent classification of these combinations into four classes: *synergistic, additive, antagonistic and inconclusive*. By using DBSumNeg, we do not observe antagonistic combinations (since by definition their DBSumNeg must be 0). However, it is clear that many combinations in the MST are not synergistic based on manual inspection. Given that the bulk of them were manually classified as *inconclusive* suggests that they are borderline and that the classification is somewhat subjective. For example, Supplementary Figure 23 displays two response matrices, both considered to be synergistic according to DBSumNeg, but visual inspection suggests that one is in fact *inconclusive* (in the sense that the response matrix is too noisy to conclude that the response is synergistic, antagonistic or additive).

### Single cell photometry

Live parasites within iRBC were imaged under constant perfusion using a custom single–cell photometry apparatus described previously^48^.

### Mitochondrial depolarization

Measurement of mitochondrial membrane potential perturbation was assessed in synchronized parasites using the fluorescent probe JC1 (Invitrogen, Carlsbad, CA). Briefly, synchronized ring stage parasites were inoculated into serially diluted compound or no-drug control wells, as previously described for *P. falciparum* growth inhibition assays^49^. Drug assay plates were then incubated in 5% O_2_/5% CO_2_/90% N_2_ gassed chambers for 24 hrs at 37 °C. Post-incubation 30 μl of each resuspended well was transferred to a replicate plate with 100 μl 0.9% NaCl/0.2% Dextrose (Baxter Healthcare, Deerfield, IL) and centrifuged at 500 × *g* for 1 min. The supernatant was removed and cells were resuspended in 50 μl of 0.9% NaCl/0.2% Dextrose (Baxter Healthcare, Deerfield, IL) with 2 μM JC1 dye (Invitrogen) and 1 μM Syto61 dye (Invitrogen). Plates were incubated for another 30 min in gassed chambers at 37 °C. Post-incubation plates were spun, and cells washed once, and resuspended in 100 μl NaCl/Dextrose solution. Fluorescent intensities were analyzed by flow cytometry on an Accuri C6 flow cytometer (BD Biosciences, Franklin Lakes, NJ). An initial gate FSC/SSC was used to gate on single cells, followed by gating on Syto61 DNA positive cells (*P. falciparum* infected erythrocytes). A fluoresce intensity ratio of the green (fluorescence channel 1, FL1) and red (fluorescence channel 2, FL2) channels was used to assess the altered mitochondrial membrane potential following the manufacturers recommendation. CCCP (carbonyl cyanide 3-chlorophenylhydrazone), provided by the manufacturer was used as a positive control.

### Immunohistochemistry

Parasitized RBC were gassed (5% CO_2_/5% O_2_, balance N_2_) and incubated at 37 °C. For drug treatments, highly synchronized mid stage trophozoites were treated as described^50^ using drug concentrations noted in the text. Resultant cell pellets were resuspended in PBS and treated as below. Cells were washed 3 times with PBS, fixed with 4% formaldehyde/0.0075% glutaraldehyde in PBS for 30 minutes, permeabilized with 0.1% Triton X-100 for 10 minutes, reduced with 0.3 mg mL^-1^ sodium triacetoxyborohydride for 10 minutes, blocked with 5% goat serum for 1 hour, and sequentially treated with antibodies diluted in 5% goat serum/PBS Tween-20 with PBS Tween-20 washes in between. The primary ATG8 antibody was raised in rabbit^30^; the secondary antibody was raised in goat and conjugated to DyLight 649 fluorophores (Jackson Immunoresearch). Cells were attached to #1.5 coverslips and mounted using “Fluorogel” mounting media. Samples were imaged using a customized Nikon Eclipse TE 2000-U spinning disk confocal microscope with either 405, 491, 561, or 642 nm laser lines (depending on probe) and with 200 ms exposure and 35% laser power. For primary antibodies raised in rabbit, primary solutions were prepared at 1:250 dilution and secondary solutions (goat anti rabbit DyLight649) at 1:500.

### Cell fluorescence data analysis

Cell fluorescence images were iteratively deconvolved using an experimental point spread function obtained under identical imaging conditions (via doping one sample with fluorescent beads) and running multiple iterations in AutoQuantX2^51^. Images were further processed and overlayed using Imaris 7.5.2 software. Using the “spots” routine in Imaris 7.5.2, puncta were defined and distances were measured from each spot to a single point within the DV as defined by the center of hemozoin optical density^30^,^51^. These distances were exported to excel and the data were plotted as number of puncta vs distance from hemozoin^30^.

### Manual cytostatic (IC_50_) and cytocidal (LD_50_) determinations

Manual antiplasmodial cytostatic (growth inhibitory, or IC_50_) and cytocidal (cell killing, or LD_50_) activity was determined for the strains discussed above essentially as previously described^40^,^50^, with minor modifications. The cytocidal assay utilizes a 6 h bolus dose with high concentrations of drug followed by washing drug away and growth in the absence of drug for 48 h, while the cytostatic assay utilizes continuous growth for 72 h in the constant presence of low concentrations of drug. For both assays, test compounds were dissolved in either deionized water, 50% EtOH, or DMSO, depending on solubility.

In the cytostatic assay, serial drug dilutions were made using complete media and 100 μL aliquots were transferred to 96-well clear-bottom black plates. Culture is prepared by first generating a Giemsa smear to determine parasitemia, and then set at 4% hematocrit 1% parasitemia by “diluting” with fresh uninfected red blood cells. Following addition of 100 μL of the culture to the plated drug concentrations, plates were transferred to an airtight chamber gassed with 5% CO_2_/5% O_2_/90% N_2_ and incubated at 37 °C.

For the cytocidal assay, drug/parasite mixture was incubated with bolus drug dose for 6 h followed by centrifugation with an Eppendorf 5415 D microcentrifuge (Hauppauge, NY) at 1800 rpm for 1 min. Drug-containing media was removed and cell pellets washed three times with drug-free complete media to remove drug^50^. Washed cytocidal assay plates and cytostatic assay plates were incubated at 37 °C for 48 h. After 48 h, 50 μL of 50X SYBR Green I dye (diluted using complete media from a 10,000X DMSO stock) was added and plates incubated for an additional 1 hr at 37 °C to allow DNA intercalation. Fluorescence was measured at 538 nm emission (485 nm excitation) using a Spectra GeminiEM plate reader (Molecular Devices; Sunnyvale, CA) fitted with a 530 nm long-pass filter. Linear standard curves of measured fluorescence vs. known parasitemia were prepared to calibrate fluorescence data^40^. Reported values are the average of three independent assays, with each assay conducted in triplicate (nine determinations total) and reported ± standard error of the mean (S.E.M.), unless otherwise noted.

### Modified *in vivo* suppression test

A modified Peter’s Suppressive Test was used to initially assess drug-drug interactions in the mouse malaria model^52^. *In vivo* tests were performed under NIH approved animal protocol LMVR 11E and in accordance with all stated guidelines. Female CD1 or BALB/c mice (BALB/c mice were used for the NVP-BGT226 experiments; Charles River, Wilmington, MA) were infected *i.p*. with 10^6^ *P. berghei* N parasites. Between two and three hours post infection drug treatment was initiated by oral gavage of compound resuspended in standard suspension vehicle (0.5% hydroxyethyl cellulose and 0.1% Tween-80; Sigma, St. Louis, MO). Compound administration was continued for an additional two days (3 consecutive days of compound administration in total). Compound dosage was adjusted by weight. Parasitemias were determined by microscopic examination of Giemsa-stained blood films taken daily, beginning on day two.

### Thompson’s modified *in vivo* test

A modified Thompson’s 30-day Test was used to assess the curative activity of drug combinations in the mouse malaria model^52^. *In vivo* tests were performed under NIH approved animal protocol LMVR 11E and in accordance with all stated guidelines. Female BALB/c mice, five per group (Charles River, Wilmington, MA), were infected *i.p*. with the rodent malaria line *P. berghei* N. Parasitemia was allowed to increase to between 3–4%, higher than the normal Thompson’s Test of 0.5–2%, to allow assessment of drug rate-of-action. Once the desired parasitemias were reached, compounds were administered for three days by oral gavage, as described above. Blood was drawn daily to assess parasitemia by microscopic examination of Giemsa-stained blood films, and hematocrit was determined by hemocytometer allowing absolute calculation of the number of infected RBCs per μl. Rate-of-action was determined by transforming the number of infected RBCs per μl. Smears in which no parasites were detected in 1,000 RBCs were assigned the lowest value calculated (0.01% parasitemia, 1,835,300 RBCs/μl; 183.53 iRBCs/μl; 5.2 LN transformed iRBCs/μl).

## Additional Information

**How to cite this article**: Mott, B. T. *et al.* High-throughput matrix screening identifies synergistic and antagonistic antimalarial drug combinations. *Sci. Rep.* **5**, 13891; doi: 10.1038/srep13891 (2015).

## Supplementary Material

Supplementary Information

## Figures and Tables

**Table 1:** Noteworthy combination results from >4,000 discreet combinations tested^a^

Drug A^b^	Mechanistic Class^c^	Drug B^b^	Mechanistic Class^c^	Comb. Effect (DBSumNeg)^d^	*In vivo* validation?^e^
ATM	Endoperoxide	Alvespimycin	HSP90 inhib.	−6.08	N.T.
ATM	Endoperoxide	M.B.	oxid. stress inducer	−5.97	N.T.
ATM	Endoperoxide	NVP-AUY992	HSP90 inhib.	−6.84	N.T.
ATM	Endoperoxide	NVP-BGT226	PI3K inhib.	−8.29	Yes
ATM	Endoperoxide	Reserpine	Ca^2+^ channel inhib.	−9.71	N.T.
ATM	Endoperoxide	Quisinostat	HDAC inhib.	−5.77	N.T.
ATM	Endoperoxide	KN-62	CaM kinase II inhib.	−10.92	N.T.
ATM	Endoperoxide	LUM	Heme conversion inhib.	−4.05	Yes
AS	Endoperoxide	MFQ	Heme conversion inhib.	−5.72	N.T.
AS	Endoperoxide	Propafenone	Na^+^ channel inhib.	−9.51	N.T.
DHA	Endoperoxide	LUM	Heme conversion inhib.	−4.94	N.T.
DHA	Endoperoxide	M.B.	oxid. stress inducer	−3.49	N.T.
LUM	Heme conversion inhib.	NVP-BGT226	PI3K inhib.	−2.33	Yes
LUM	Heme conversion inhib.	Manidipine	Ca^2+^ channel inhib.	−7.69	N.T.
LUM	Heme conversion inhib.	Rifampin	Protein synthesis inhib.	−4.99	N.T.
LUM	Heme conversion inhib.	Midostaurin	multikinase inhib.	−4.7	N.T.
MFQ	Heme conversion inhib.	Nicardipine	Ca^2+^ channel inhib.	−7.45	N.T.
MFQ	Heme conversion inhib.	Midostaurin	multikinase inhib.	−4.76	N.T.
AQ	Heme conversion inhib.	BIX-01294	methylation inhib.	−4.38	N.T.
Atovaquone	e^−^ transport chain inhib.	ML238	e^−^ transport chain inhib.	−7.78	N.T.
Atovaquone	e^−^ transport chain inhib.	Decoquinate	e^−^ transport chain inhib.	−3.28	N.T.
Atovaquone	e^−^ transport chain inhib.	Genz-669178	PfDHODH inhib.	−3.18	N.T.
ML238	e^−^ transport chain inhib.	Genz-669178	PfDHODH inhib.	−5.4	N.T.
NITD-609	Protein synthesis/PfATPase4 inhib.	Trichostatin A	HDAC inhib.	−2.43	N.T.
Leptomycin B	Nuclear export inhib.	Nanchangmycin	Antibiotic	−3.57	N.T.

^a^Data is against *P. falciparum* 3D7 and represents the lowest values from duplicate screens. Complete data sets can be found at https://tripod.nih.gov/matrix-client/rest/matrix/blocks/1761/table.

^b^Artemether (ATM), artesunate (AS), dihydroartemisinin (DHA), lumefantrine (LUM), mefloquine (MFQ), amodiaquine (AQ), methylene blue (M.B.).

^c^Mechanistic class reflects general terms and is not intended to cover all putatively contributing pharmacology’s of the drugs listed (may reflect hypothesized mechanism based on mammalian target).

^d^Combination effect reflects the DBSumNeg value.

^e^Yes indicates *in vivo* study presented in this study only. N.T. (not tested).

**Table 2:** Comparative analysis of combination responses in Dd2 as judged by single point Chou-Talalay analysis for selected drug combinations at IC_50_ and LD_50_ concentrations (n = 3, +/− SEM values provided in SI) and at the anticipated *in vivo* concentration (AIVC). Values for HB3 are provided in table S8.

Drug Combination	Avg. FIC		Avg. FIC_Index_	Avg. FLD		Avg. FLD_Index_	Avg. FAIVC^a^		Avg. FAIVC_Index_
	Drug A	Drug B		Drug A	Drug B		Drug A	Drug B	
ATM + LUM	0.67	0.47	1.1 (Add.)	0.42	0.58	1.0 (Syn.)	ND	ND	ND
ATM + GSK-2126458	0.54	0.52	1.1 (Add.)	0.18	0.21	0.39 (Syn.)	ND	ND	ND
ATM + NVP-BGT226	0.97	1.05	2.0 (Add.)	0.27	0.46	0.93 (Syn.)	0.85	0.14	0.99 (Syn.)
ATM + Torin 2	0.75	0.59	1.3 (Add.)	0.54	0.60	0.94 (Syn.)	ND	ND	ND
LUM + GSK-2126458	0.27	0.40	0.67 (Syn.)	0.20	0.15	0.35 (Syn.)	ND	ND	ND
LUM + NVP-BGT226	0.60	0.93	1.5 (Add.)	1.10	1.17	2.0 (Ant.)	0.45	0.05	0.49 (Syn.)
LUM + Torin 2	0.44	0.48	0.9 (Syn.)	0.50	0.19	0.5 (Syn.)	ND	ND	ND

^a^Data indicates a ratio of ATM:NVP-BGT226 of 100:1 and a ratio of LUM:NVP-BGT226 of 500:1. Additional ratios for ATM:NVP-BGT226 include 50:1, 200:1, and 400:1 and for LUM:NVP-BGT226 include 250:1, 1250:1, and 25000:1. These data are presented in table S8. FIC (fractional inhibitory concentration); FLD (fractional lethal dose); FAIVC (fractional anticipated *in vivo* concentration); ATM (artemether); LUM (lumefantrine); Add. (additive); Syn. (synergistic); Ant. (antagonistic).
