# Supplementary material for: High-throughput matrix screening identifies synergistic and antagonistic antimalarial drug combinations
Source: Sci Rep. 2015 Sep 25;5:13891. doi: 10.1038/srep13891 (PMC4585899; doi:10.1038/srep13891)
Supplement: Supplementary Information [file srep13891-s1.pdf]

Supplementary Materials for

High-throughput matrix screening identifies synergistic and antagonistic antimalarial drug combinations

Bryan T. Mott, Richard T. Eastman, Rajarshi Guha, Katy S. Sherlach, Amila Siriwardana, Paul Shinn, Crystal McKnight, Sam Michael, Norinne Lacerda-Queiroz, Paresma R. Patel, Pwint Khine, Hongmao Sun, Monica Kasbekar, Nima Aghdam, Shaun D. Fontaine, Dongbo Liu, Tim Mierzwa, Lesley A. Mathews-Griner, Marc Ferrer, Adam R. Renslo, James Inglese, Jing Yuan, Paul D. Roepe, Xin-zhuan Su and Craig J. Thomas

Correspondence to: roepep@georgetown.edu (P.D.R.); xsu@niaid.nih.gov (X.-z.S.); craigt@mail.nih.gov (C.J.T.)

This PDF file includes:

Materials and Methods
Figs. S1-S28
Tables S4-S5; S7-13; S40-S41

Other supporting materials for this manuscript can be found online at http://tripod.nih.gov/pub/malaria-matrix/ and include the following:

Excel tables S1-S3; S6; S14-S39

Table S1. Single agent data for compounds screened against 3d7, Hb3 and Dd2 *P. falciparum* isolates.
Table S2. Summary of matrix combination screening data against *P. falciparum*
Table S3. Complete analysis of each combination screened against *P. falciparum*
Table S6. Combinations for minimum spanning tree analysis
Table S14. *P. falciparum* combination high-throughput screen data quality control filtered
Table S15. *P. falciparum* self-cross interactions
Table S16. *P. falciparum* 3D7 cHTS data quality control filtered
Table S17. *P. falciparum* Dd2 cHTS data quality control filtered
Table S18. *P. falciparum* HB3 cHTS data quality control filtered
Table S19. *P. falciparum* 3D7 interactions with a DBSum negative value less than 3
Table S20. *P. falciparum* Dd2 interactions with a DBSum negative value less than 3
Table S21. *P. falciparum* HB3 interactions with a DBSum negative value less than 3
Table S22. *P. falciparum* 3D7 interactions with a DBSum positive value greater than 3
Table S23. *P. falciparum* Dd2 interactions with a DBSum positive value greater than 3
Table S24. *P. falciparum* HB3 interactions with a DBSum positive value greater than 3

Table S25. Aggregated *P. falciparum* interactions with artemether with a DBSum negative value less than 3
Table S26. Aggregated *P. falciparum* interactions with artesunate with a DBSum negative value less than 3
Table S27. Aggregated *P. falciparum* interactions with dihydroartemisinin with a DBSum negative value less than 3
Table S28. Aggregated *P. falciparum* interactions with lumefantrine with a DBSum negative value less than 3
Table S29. Aggregated *P. falciparum* interactions with halofantrine with a DBSum negative value less than 3
Table S30. Aggregated *P. falciparum* interactions with mefloquine with a DBSum negative value less than 3
Table S31. Aggregated *P. falciparum* interactions with chloroquine with a DBSum negative value less than 3
Table S32. Aggregated *P. falciparum* interactions with atovaquone with a DBSum negative value less than 3
Table S33. Aggregated *P. falciparum* interactions with pyronaridine with a DBSum negative value less than 3
Table S34. Aggregated *P. falciparum* interactions with piperaquine with a DBSum negative value less than 3
Table S35. Aggregated *P. falciparum* interactions with tafenoquine with a DBSum negative value less than 3
Table S36. Aggregated *P. falciparum* interactions with amodiaquine with a DBSum negative value less than 3
Table S37. Aggregated *P. falciparum* interactions with sulfadoxine with a DBSum negative value less than 3
Table S38. Aggregated *P. falciparum* interactions with NITD609 with a DBSum negative value less than 3
Table S39. Aggregated *P. falciparum* interactions with NITD609 with a DBSum negative value less than 3

QC criteria

The quality control score is a numerical characterization of the quality of a combination that attempts to take into account single agent performance and the presence of noise in the dose combination region of the matrix. It is composed of a number of heuristics, developed by examination of a series of matrix screening runs. The calculation is performed on the bounded (0-100) response matrix and considers the following conditions:

1. DMSO response lies between 80 and 100.
2. Both single agents should display a valid dose response and provide a valid IC_{50} value.
3. If single agents have a valid curve fit, the responses (across the whole concentration range as well when the top concentration is excluded) should have a relative standard deviation greater than 20 (*35*)
4. The relative standard deviation of the dose combination sub-matrix should be greater than 25
5. The responses in the dose combination sub-matrix should exhibit spatial autocorrelation (i.e., combination responses should not be randomly distributed within the dose matrix). This is measured by Morans I (*39*). We consider a combination to exhibit spatial autocorrelation if the p-value is less than 0.05

Each combination is tested against the five conditions and the result is expressed as a vector, defined as

$$C = \{C_i\}, i = 1 \dots 5$$

where $C_i = 1$ if the i'th condition is true, 0 otherwise.

The final score is obtained by computing the dot product of the binary vector and a weight vector, W of the same length as C. The latter allows us to assign an importance to each of the five conditions noted above. The final score is given by

$$QCScore = \sum_{i=1}^{5} C_i W_i$$

Currently, the weight vector is defined as $W = \{2,5,3,5,3\}$, allowing for a minimum score of 0 (the combination passes all QC criterion) and maximum score of 18 (combination fails all QC criterion).

A combination with a score of 0 is deemed as good quality and suitable for further consideration. Increasing values of the QCScore indicate poorer quality combination responses (Fig S22).

It should be noted that the score can identify false positive – combinations that are scored well, but on visual inspection turn out to be poor quality. The score is meant to provide an initial ranking and should not preclude manual inspection. Each matrix block was ultimately manually classified as synergistic, additive, antagonistic, or inconclusive.

Additional matrix metrics

In addition to the previously described metrics (*15*) we expanded our analysis to include two new quantifiers of synergy – DBSumNeg and DBSumPos. Considering all dose combinations tested, these are defined as the sum of positive deviations from the Bliss model and the sum of the negative deviations from the Bliss model, respectively. In contrast to simply summing all deviations from the Bliss model, these two variables characterize the extent of synergy and antagonism, respectively, within a set of dose combinations.

Expanded isobologram analyses

Many studies evaluating drug synergies rely upon static values such as isobolographic analyses or combination indices (CI) derived from the multiple drug effect equation developed by Chou and Talalay (*40-42*). The CI method is based upon discreet concentration responses and reports generally rely upon using data thought to reflect the overall combination response of the two agents. We chose not to list CI values within our web-based ranking options as many combinations revealed overlapping combination effects (synergy and additivity – or – synergy and antagonism). Although isobolograms act an additional means to visualize synergy between two agents we were not able to automate the drawing of isobolograms for each data set. Further, our dosing was reliant on standard dilutions from a starting value and do not necessarily provide large numbers of fixed ratio dosing. Isobolograms for selected combinations were generated using Compusyn (Combosyn, Inc, version 1.0) and are presented in Fig 2A (panel 4). The 10×10 data sets used to derive them are shown in Fig S9.5 D-F (for ATM + KN-62) and Fig S9.1 D-F (for ATM + reserpine). From these, we derived Fa-CI plots that demonstrate these combinations are synergistic at relevant doses (Fa>0.5 or Fa>0.75) at all ratios tested.

Clustering analyses

Based on the QCScore values we selected a subset of 1760 combinations that were deemed to be of good to medium quality (QCScore < 5). We then characterized each combination in terms of three parameters

1. Potency class of each single agent in the combination. Possible values are high potency (*HP*) and medium potency (*MP*)
2. Synergy class for the combination. Possible values are *additive, antagonistic, inconclusive and synergistic*.
3. A mechanism of action (MOA) class for each single agent in the combination. Possible values are *antimalarial, growth inh, ion channel, mito, PI3K/mTOR, signaling/transport*

We then converted these labels to a binary fingerprint using the following scheme. The potency class for each single agent is coded as a 2-bit vector where the first bit is set to 1 if the single agent is labeled as MP and the second bit is 1 if it is labeled as HP. Similarly, the synergy class is encoded as a 4-bit vector, where a single bit is set to 1 depending on

the synergy class label assigned to the combination. Finally, the MOA class for each single agent is represented as a 6-bit vector.

Thus for a given combination, we end up with five bit vectors and the final fingerprint for each combination is simply the concatenation of the individual bit vectors. Thus the fingerprint is a 20-bit vector, whose bit positions are labeled as shown in Fig. S23. We compute the fingerprint for all 1760 combinations and then evaluated a pairwise similarity matrix using the Tanimoto metric. We investigated the use other similarity metrics (cosine and Tversky) and observed no differences in the resultant analyses.

The similarity matrix was converted to a dissimilarity matrix (by subtracting each element from 1.0), which was then used to compute a hierarchical clustering. While there is much literature on identifying clusters, there is no consensus on how one might identify the optimal number of clusters.

In this study we were interested in identifying clusters that were statistically enriched (see below for details) in one or more MOA class pairs (i.e., considering the MOA of both single agents, table S40). As a result we identified the largest number of clusters such that every cluster was enriched in at least one MOA class pair. This procedure identified 6 clusters. The complete dendrogram, color coded according to arbitrary cluster number is shown in Fig. 1D.

It is useful to summarize the combinations belonging to a given cluster. We considered two approaches. First, we examined the occurrence of specific fingerprint bits within clusters. Second, we computed enrichments of MOA combinations within each clusters.

1. Parameter distributions

To examine how individual bits (say, the bit corresponding to synergy class of *synergistic*) are distributed within a cluster we use the concept of a bit spectrum (*44*). Given m combinations each represented using an n-bit fingerprint, we evaluate the fraction of combinations for which the i'th bit is set to one. This is repeated for all bit positions, resulting in a floating-point vector that is termed the bit spectrum for that set of combinations. Formally, the i'th value of the bit spectrum is defined as

$$BS_i = \frac{1}{m}\sum_{j=1}^{m} f_{i,j}$$

where $f_{i,j}$ represents the i'th bit in the fingerprint for the j'th combination and $1 < i < n$.

Since the values range from 0 to 1, this allows us to compare distributions of bit positions between different sets of combinations. We evaluated bit spectra for the 6 clusters obtained above and these are summarized in Fig. S24.

For example, the 886 combinations comprising cluster 1 (red) have single agents that are primarily anti-malarial and ion channel inhibitors. In contrast the 14 combinations comprising cluster 6 have single agents that are nearly all signaling/transport modulators.

At the same time, the combinations in cluster 6 were primarily labeled as exhibiting inconclusive combination responses.

2. MOA enrichment

We next evaluated the enrichment of MOA class pairs – i.e., the pair of MOA classes represented by the single agents in a combination. Given the 6 possible MOA classes, there are 21 possible MOA pairs (which includes pairs where both members are from the same MOA class). We performed a Fishers exact test (*45*) to evaluate whether a cluster was enriched in a given MOA class pair, compared to the dataset as a whole. The 21 pairs of MOA classes were coded as shown in the adjoining table. Fig. S25 summarizes the enrichment analysis by plotting the negative $\log_{10}$ of the Benjamini-Hochberg (*45*) corrected p-values for each MOA class pair. The red and blue lines correspond to $p = 0.01$ and $p = 0.05$ respectively. All calculations were performed using R 3.0.1 (*47*) on a Macbook Pro (OS X 10.9.1, 16GB RAM).

Single cell photometry

Live parasites within iRBC were imaged under constant perfusion using a custom single – cell photometry apparatus described previously (*48*). Coverslips were prepared by adding 500 µL of 0.1% (w/v) poly-L-lysine and incubating for 10 min to coat the coverslip. They were then dried and stored at 4º C until use. Trophozoite-infected RBCs were resuspended at 0.5% hematocrit in incomplete media with 25 mM HEPES, pH 7.4, and 200 µL was placed on a poly-L-lysine coated coverslip. The cells were incubated for 3 min under standard cell culture atmosphere. Non-adherent cells were washed off and the coverslip was mounted on a custom-designed perfusion chamber for microscopy work. For Ca^{2+} imaging, cells were incubated with Fura-2 AM at a final concentration of 5 µM. 0.1% v/v Pluronic F-127 was added to improve dye loading into iRBCs. For perfusion experiments, the parasites were maintained under constant perfusion at 1 mL/min of physiologic buffer (HBSS balanced with 5% O_2 / 5%CO_2 / 90% N_2 at 37 ºC). Changes in cytosolic Ca^{2+} upon drug addition were monitored by switching to identical perfusate containing drug at the indicated concentrations. Fura-2 ratiometric values vs time were collected for individual cells, data were converted to [Ca^{2+}] as described below, and Ca^{2+} - transients for $\geq$ 15 parasites were then averaged at each drug concentration.

The single cell photometry (SCP) apparatus includes a custom-built Nikon Diaphot epifluorescence microscope equipped with a 100X oil immersion objective capable of UV transmission (Fluoro, N.A. 1.25, 160/017), and a 16-bit Sensys CCD camera (Tucson, AR) attached to the side port of the microscope. Excitation light was provided by a computer controlled xenon arc lamp (LAMBDA LS, Novato, CA). 2 band-pass filters at 340 nm and 380 nm filtered UV light for ratiometric illumination of Fura-2 (Asahi Spectra Co., Ltd., San Jose, CA), and were housed in a Lambda-10 filter wheel controlled via acquisition software (Imaging Workbench). The excitation light was transported by a liquid light guide (Novato, CA), collimated, and passed through the microscope optics. A filter cube housing a 400 nm dichroic mirror combined with a 410 nm long-pass filter separated excitation from emission. Light power before the objective was monitored with a near UV power meter (Metrologic Model No. 45-545). Exposure

time was typically set at 500 ms followed by a 10 sec recovery in total darkness. For ratiometric measurements, data at alternate excitation wavelengths (340 and 380 nm) were collected from a cytosolic region of interest (ROI) within the parasite, while a second region of interest of the same dimensions was drawn outside the iRBC and imaged simultaneously background subtraction.

In situ calcium calibration of Fura-2

For calibration, parasites were loaded with Ca^{2+} dye as above, and perfusates were prepared from 2 stocks: 1) HBSS + 25 mM HEPES + 10 mM EGTA (pH 7.4); 2) HBSS + 10 mM EGTA +25 mM HEPES + 10 mM $CaCl_2$ (pH 7.4). Solutions with specific concentrations of free Ca^{2+} were prepared by mixing the stocks at different ratios. The free Ca^{2+} concentration was calculated using the "MAXCHELATOR" program (provided by Professor Chris Patton from Stanford University http://maxchelator.stanford.edu/index.html); parameters were set at pH 7.4, 37 ºC, EGTA, ionic strength 150 mM. Before calibration experiments, 10 µM ionomycin was added in order to clamp the internal free calcium concentration to the concentration in the perfusate. Intact trophozoite-stage parasites were first perfused with Ca^{2+}-free buffer at a rate of 1 mL/min, and data collected for ≥ 15 parasites. This process was repeated for each perfusate containing ionomycin and specific [Ca^{2+}] (54, 127, 217, 488, 1,000 and 23,700 nM); ratiometric data were collected for ≥ 15 cells at each concentration and averaged. Data were processed and analyzed with Microsoft Excel and SigmaPlot 11.0. Calibration curves (e.g. Fig S26) were fitted to sigmoidal curves and Fura-2 ratiometric data obtained under perfusion with physiological perfusate +/- drug were extrapolated to determine Ca^{2+} concentrations (shown on the Y axis for plots of Ca^{2+} vs time, see example Fig S27).

Reactive oxidative stress assays

Measurement of reactive oxidative species (ROS) production was assessed in synchronized trophozoite parasites as previously described, with minor modifications (*50*). Briefly, parasites were incubated at room temperature with 10 µM H_2DCFDA (2',7'-dichlorodihydrofluorescein diacetate; Invitrogen, Carlsbad, CA) in the dark. The sample was then centrifuged at 713 × *g*, washed once with complete media and resuspended at 4% hematocrit. 100 µl was pipetted to each well of a 96-well plate that contained the pre-serially diluted compound of interest and wells without drug as negative controls assays (*49*). Plates were then incubated in the dark in 5% O_2/5% CO_2/90% N_2 gassed chambers for 3 hrs at 37 °C. After incubation, wells were resuspended by pipetting, and 30 µl was transferred to a replicate 96-well plate and 100 µl of 0.9% NaCl/0.2% Dextrose (Baxter Healthcare, Deerfield, IL) with 500 nM Syto61 (Invitrogen) was added. The sample was incubated again in the gassed chamber at 37 °C for an additional 30 min. Subsequently, the plate was centrifuged at 500 × *g* for 1 min. The supernatant was removed and the cells were washed once with 0.9% NaCl/0.2% Dextrose solution. The plate was centrifuged again, supernatant removed, and the pellet was resuspended in 100 µl of 0.9% NaCl/0.2% Dextrose solution. The plate was then analyzed by flow cytometry on an Accuri C6 flow cytometer. With an initial gate for events containing DNA (Syto61) and then for modulation of H_2DCFDA fluorescence by drug induced ROS, normalized to the no drug control (media only) wells, as previously described (*50*).

Homology model

The homology model of PfVps34 was constructed by using X-ray crystal structure of Drosophila melanogaster Vps34 (PDB ID: 2X6H)* as the template. PfVps34 shares over 36% identical residues with its template in the kinase domain. The homology modeling was carried out using the molecular modeling software MOE (v. 2012.10)(Chemical Computing Group, Montreal, Canada). The force field of Amber12:EHT was employed in homology modeling and energy minimization.

Manual drug combination assays using the Chou-Talalay method

The effect of combining two drugs together was assayed through use of the Chou-Talalay method of fixed-ratio analysis (*40*, *53*). Briefly, the two compounds were initially screened for their individual cytostatic and cytocidal activities, as described above. Combination analyses were initially run at fixed ratios of these determined values (1:1 unless noted). This results in a set of concentrations all at a multiple or fraction of the compounds' individual IC_{50}s or LD_{50}s. A combination stock, set at four times the IC50 or LD50, is serially diluted seven times to yield a range of concentrations that can generate a combination growth or survival curve, see Table S41 for a diagram of the plate layout.

These samples are processed as described above, using conditions of either the cytostatic or cytocidal assay. These data are then plotted versus each drug's individual concentrations. For each drug in each assay, there is a 50% point, termed the "pseudo"-IC_{50} or "pseudo"-LD_{50}, that represents the activity of one drug in the combination. This value is then used to generate a fractional inhibitory concentration (FIC) or fractional lethal dose (FLD) using the following equations for compounds A and B (*40, 53*):

$$FIC_A = \frac{Pseudo - IC_{50A}}{IC_{50A}} \quad (1)$$
$$FIC_B = \frac{Pseudo - IC_{50B}}{IC_{50B}}$$

By summing these two values, a combination index (or FICindex, FLDindex) can be generated (*40,53*):

$$FIC_{Index} = FIC_A + FIC_B \quad (2)$$

The FIC index of a combination is then used to assign synergy, additivity, or antagonism. The cut-off values for these designations vary (*54*), but those selected for this work were (*15*):

Synergy- FICindex ≤1.0
Additivity- 1.0< FICindex ≤2.0
Antagonism- FICindex >2.0

FICindex, FLDindex and FAIVCindex values were averaged from at least 2 independent trials, each trial done in triplicate and are shown +/- standard error of the mean.

In vivo manual drug combination assays using the Chou-Talalay method

In vivo ratios were estimated based on previously determined in vivo levels for ATM (*55*), LUM (*56*) and NVP-BGT226 (*57*).

NITD-609 Synthesis

NITD-609 was synthesized in house according to literature procedure[17], enantiomers were separated using Chiralpac AD 4.6x250 column, 60% EtOH in hexanes, 1 mL/min – yield: 255 mg (48 % of pure enantiomer); ^{1}H NMR (400 MHz, DMSO-d_6) δ 10.69 (s, 1 H), 10.51 (s, 1 H), 7.42 (d, *J* = 10.1 Hz, 1 H), 7.31 (ddd, *J* = 8.3, 2.2, 0.6 Hz, 1 H), 7.25 (d, *J* = 6.3 Hz, 1 H), 7.03 (d, *J* = 2.2 Hz, 1 H), 6.91 (d, *J* = 8.2 Hz, 1 H), 3.89 (d, *J* = 5.4 Hz, 1 H), 3.14 – 3.03 (m, 1 H), 2.75 (dd, *J* = 15.1, 3.7 Hz, 1 H), 2.36 (dd, *J* = 15.1, 10.5 Hz, 1 H), 1.15 (d, *J* = 6.4 Hz, 3 H); ^{13}C NMR (101 MHz, DMSO-d_6) δ 178.54, 153.17, 150.84, 142.06, 134.38, 133.18, 129.54, 126.08, 125.32, 112.42, 112.13, 111.70, 62.33, 55.36, 44.59, 41.81, 29.75, 22.04, 11.56; chiral rt = 4.28 min (enantiomer with positive α).

Supporting Figures:

A

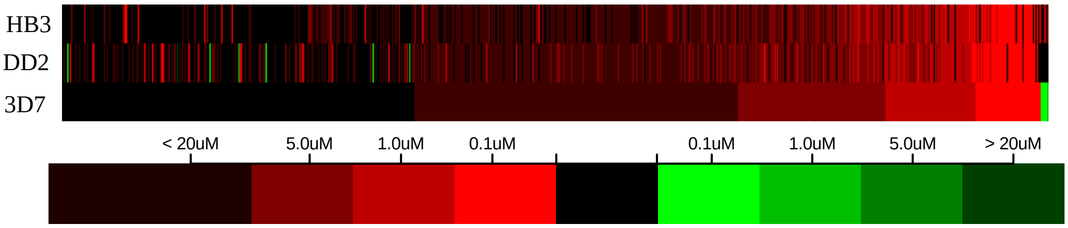

B

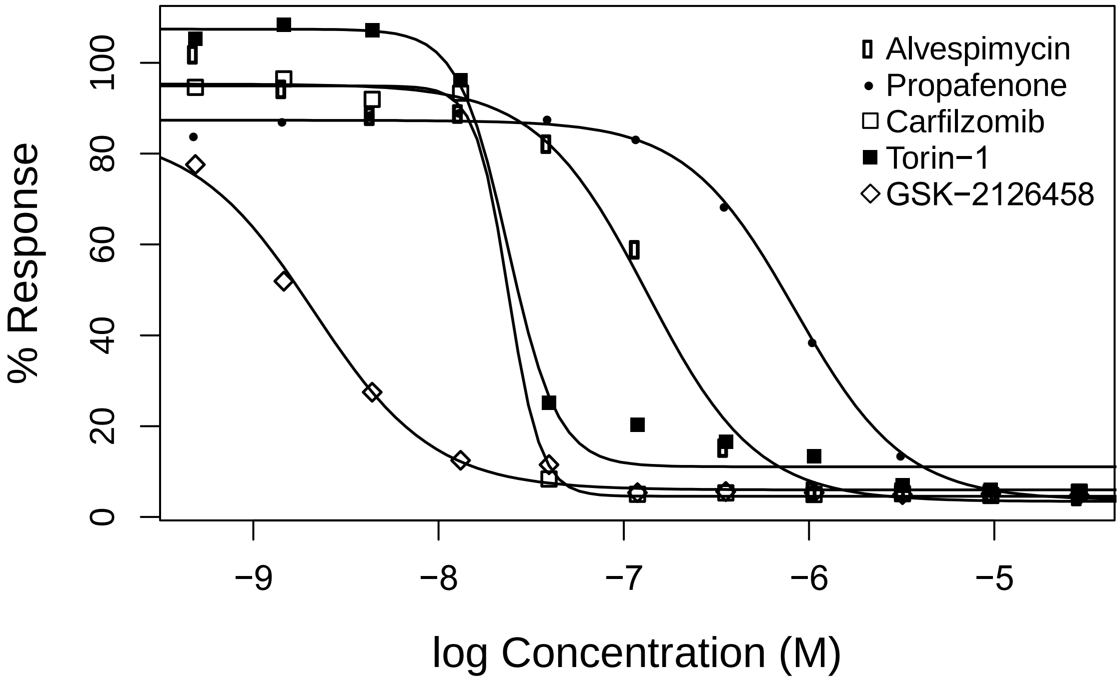

Fig. S1

(**A**) Heat map representation of 2317 approved and investigational drugs (data available at http://pubchem.ncbi.nlm.nih.gov/; summary AID: 743367). (**B**) Complete response curves for several selected agents against 3D7 parasite isolate (alvespimycin IC_{50} = 133 nM; propafenone IC_{50} = 840 nM; carfilzomib IC_{50} = 21 nM; torin 1 IC_{50} = 24 nM; GSK-2126458 IC_{50} = 2 nM).

***Note**: representative heatmaps shown below will be given the notation of assay ID and block ID in the format of "(AID/BID)" which corresponds to the representative line in Table S3.

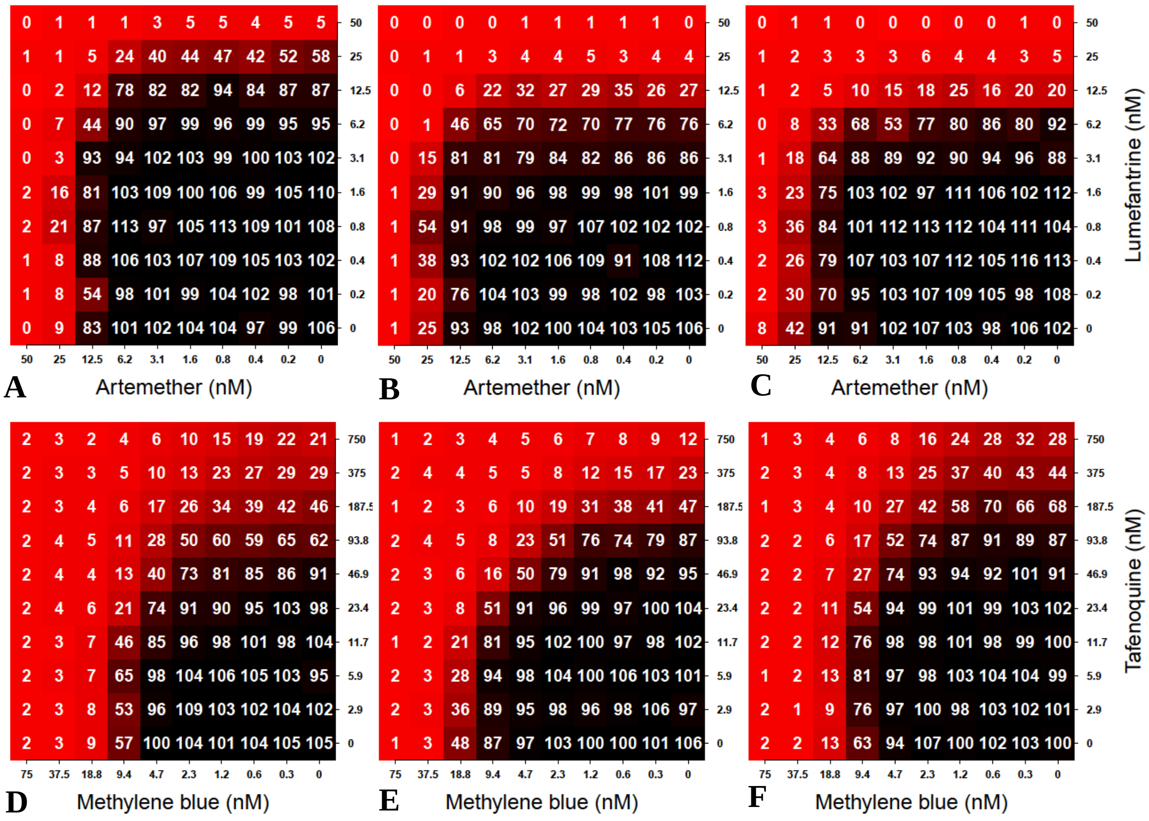

Fig. S2

Interaction heatmaps for artemether + lumefantrine against 3D7 (**A**, 1761/40), Dd2 (**B**, 1763/40) and Hb3 (**C**, 1764/40); interaction heatmaps for tafenoquine + methylene blue against 3D7 (**D**, 1761/190), Dd2 (**E**, 1763/190) and Hb3 (**F**, 1764/190). Numbers reflect the assay ID/serial for each heatmap as listed in the malaria folder at https://tripod.nih.gov/matrix-client/.

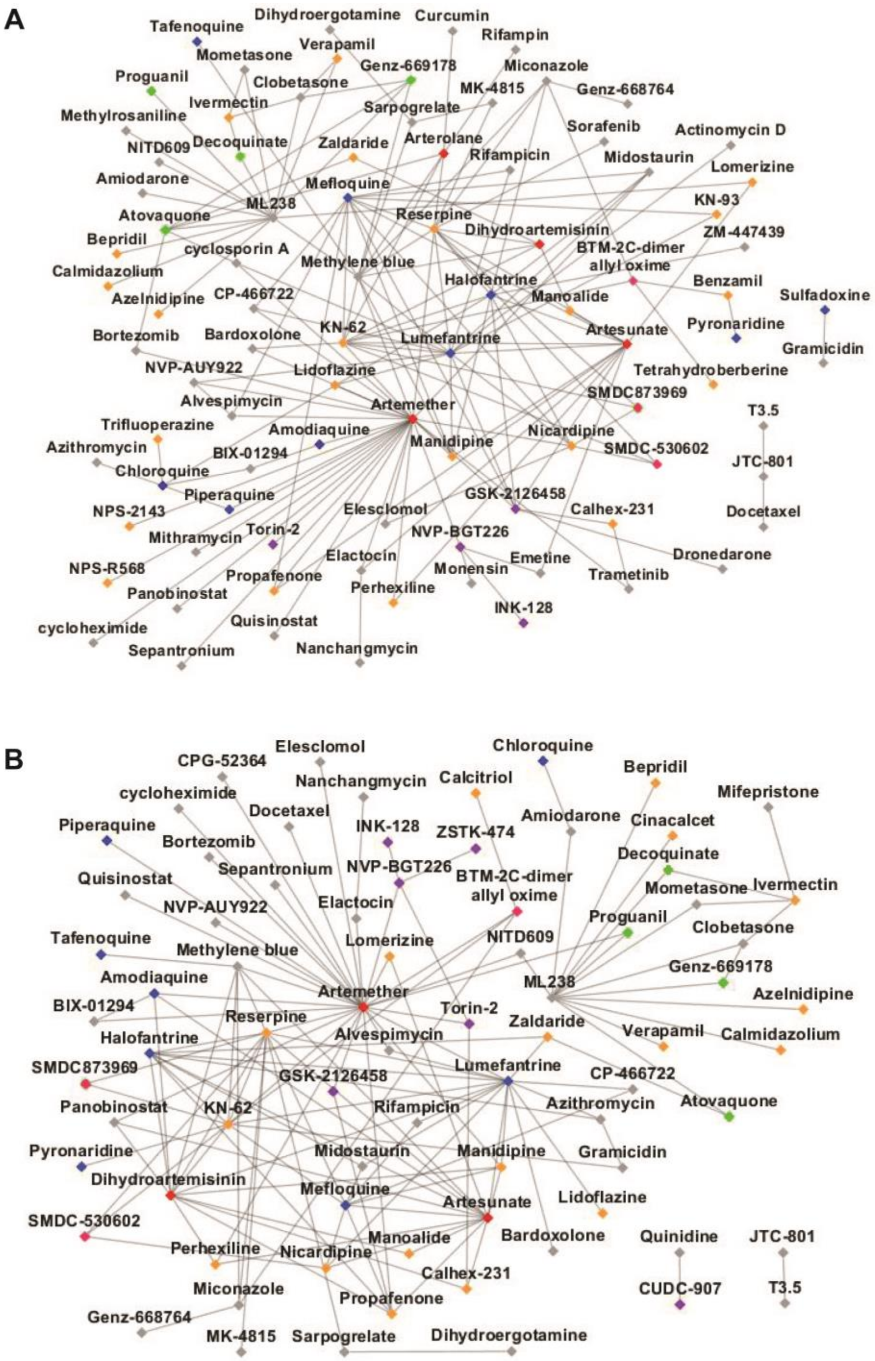

Fig. S3
Interaction Network plot of combinations passing a defined threshold (DBSumNeg < -3) for *Plasmodium falciparum* strains Dd2 (**A**) and HB3 (**B**). (■ = Endoperoxides; ■ = Halofantrine, Lumefantrine, Mefloquine, Chloroquine, Amodiaquine, Piperaquine, Pyronaridine or Tafenoquine (non-endoperoxide or mitochondrial pathway targeting antimalarials); ■ = hPI3K/mTOR; ■ = mitochondrial/DHODH; ■ = ion channel modulator; ■ = hybrid mechanism; ■ = other).

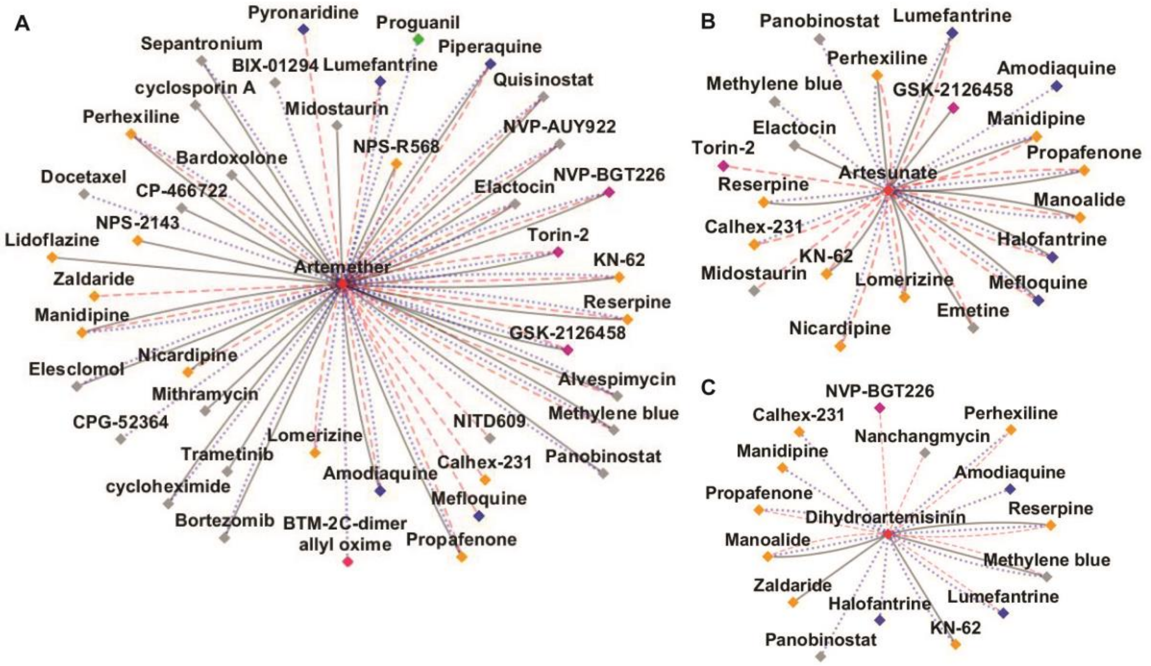

Fig. S4

Interaction Network plot of combinations passing a defined threshold (DBSumNeg < -3) interacting with Artemether (**A**), Artesunate (**B**) or Dihydroartemisinin (**C**). *Plasmodium falciparum* strains 3D7 (dashed red line) Dd2 (solid black line) and HB3 (dotted blue line). (■ = Endoperoxides; ■ = Halofantrine, Lumefantrine, Mefloquine, Chloroquine, Amodiaquine, Piperaquine, Pyronaridine or Tafenoquine (non-endoperoxide or mitochondrial pathway targeting antimalarials); ■ = hPI3K/mTOR; ■ = mitochondrial/DHODH; ■ = ion channel modulator; ■ = hybrid mechanism; ■ = other).

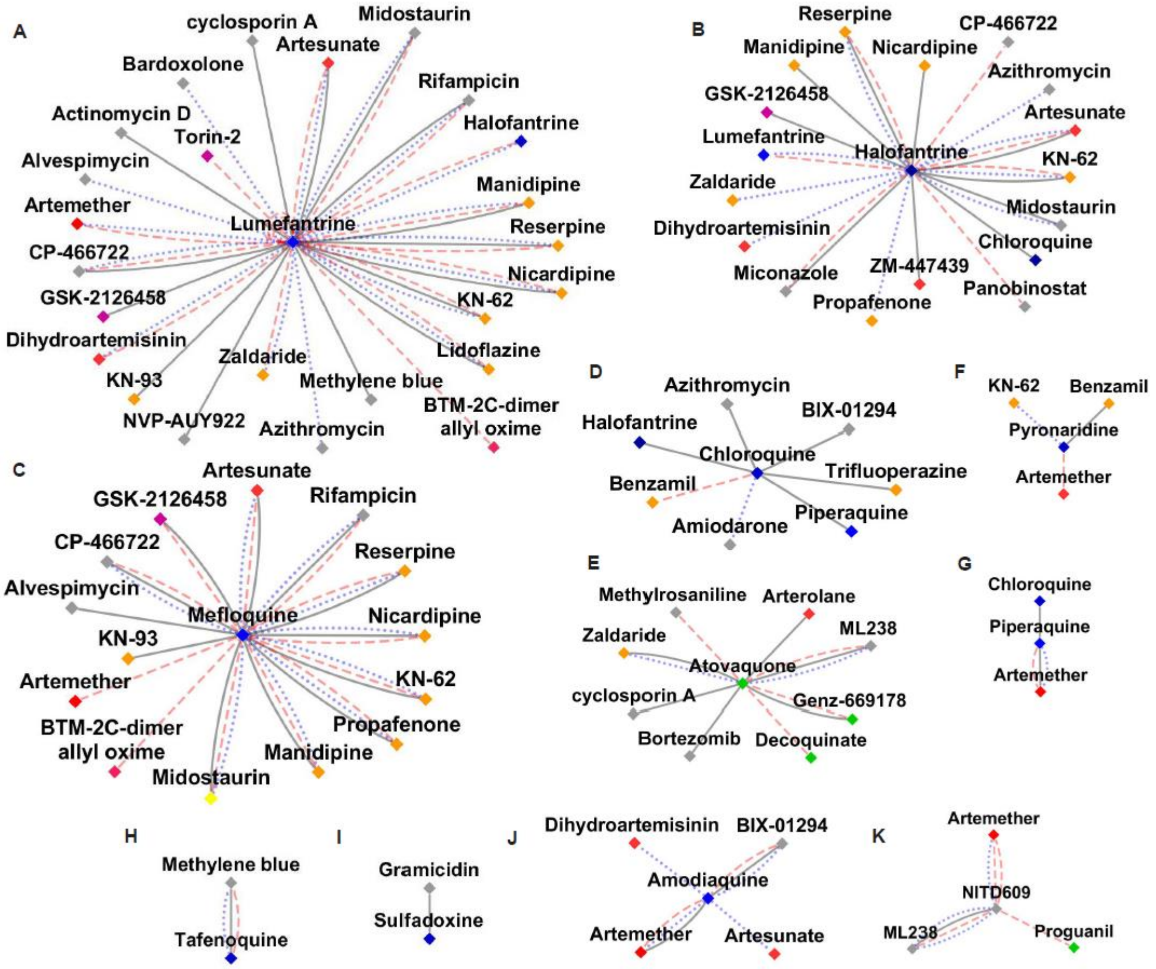

Fig. S5

Interaction Network plot of combinations passing a defined threshold (DBSumNeg < -3) interacting with current or former antimalarial agents, Lumefantrine (A), Halofantrine (B) Mefloquine (C), Chloroquine (D), Atovaquone (E), Pyronaridine (F), Piperaquine (G), Tafenoquine (H), Sulfadoxine (I) Amodiaquine (J) and NITD609 (K). Primaquine and Pyrimethamine did not have any assays with interactions meeting the criteria. *Plasmodium falciparum* strains 3D7 (dashed red line) Dd2 (solid black line) and HB3 (dotted blue line). (■ = Endoperoxides; ■ = Halofantrine, Lumefantrine, Mefloquine, Chloroquine, Amodiaquine, Piperaquine, Pyronaridine or Tafenoquine (non-endoperoxide or mitochondrial pathway targeting antimalarials); ■ = hPI3K/mTOR; ■ = mitochondrial/DHODH; ■ = ion channel modulator; ■ = hybrid mechanism; ■ = other).

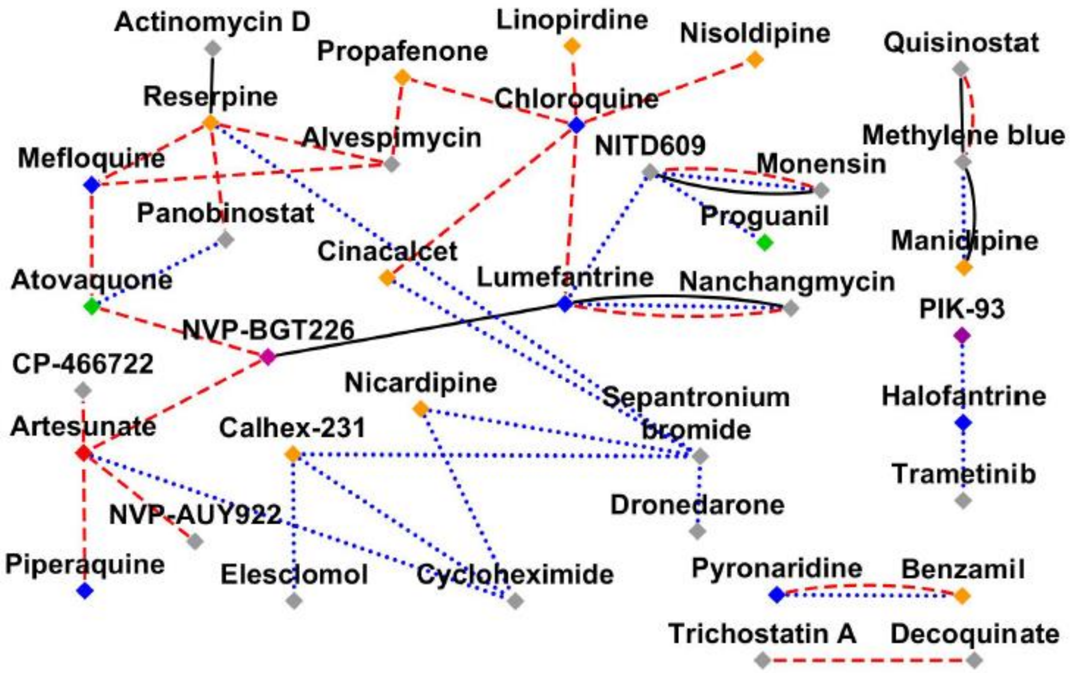

Fig. S6

Interaction Network plot of combinations passing a defined threshold (DBSumPos >3) for *Plasmodium falciparum* strains 3D7 (dashed red line) Dd2 (solid black line) and HB3 (dotted blue line). (■ = Endoperoxides; ■ = Halofantrine, Lumefantrine, Mefloquine, Chloroquine, Amodiaquine, Piperaquine, Pyronaridine or Tafenoquine (non-endoperoxide or mitochondrial pathway targeting antimalarials); ■ = hPI3K/mTOR; ■ = mitochondrial/DHODH; ■ = ion channel modulator; ■ = hybrid mechanism; ■ = other).

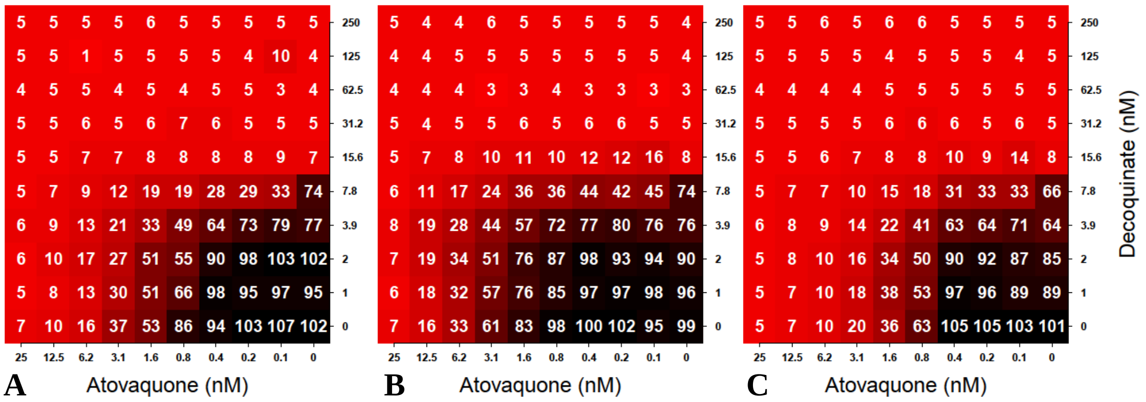

Fig. S7
Interaction heatmaps for atovaquone + decoquinate against 3D7 (**A**, 1761/190), Dd2 (**B**, 1763/190) and Hb3 (**C**, 1764/190). Numbers reflect the assay ID/serial for each heatmap as listed in the malaria folder at https://tripod.nih.gov/matrix-client/.

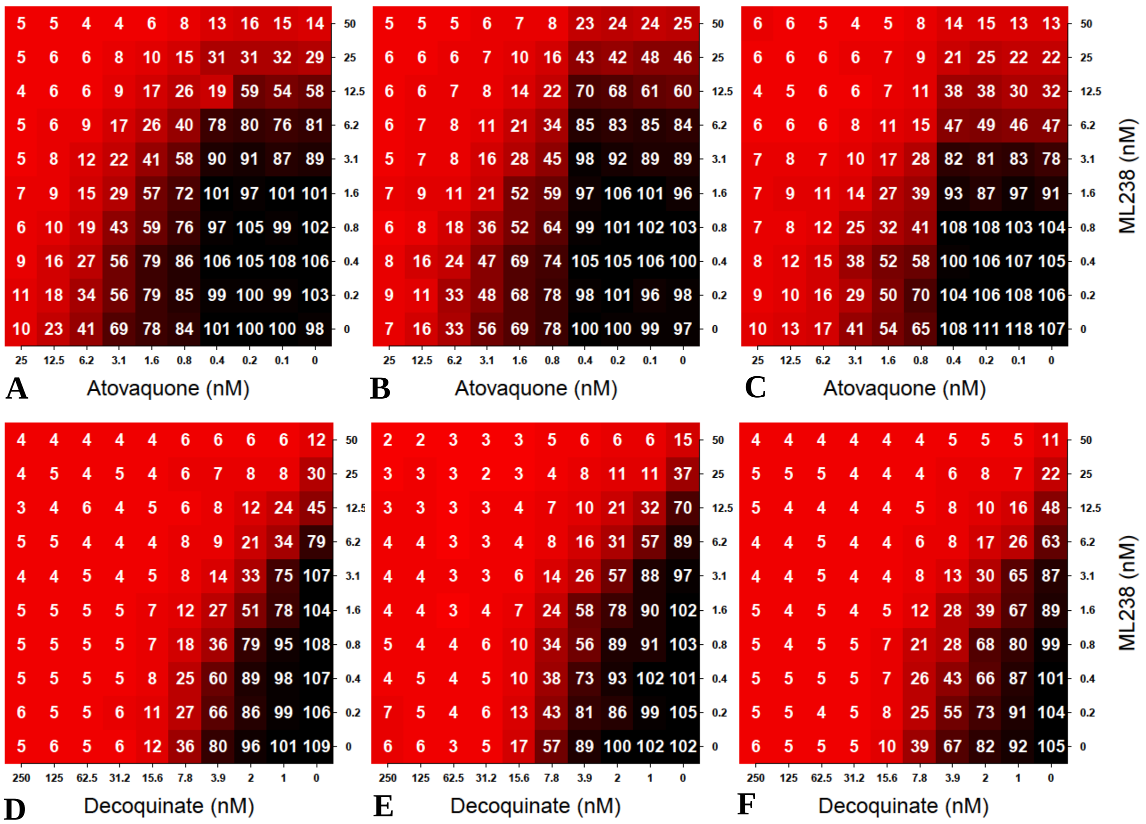

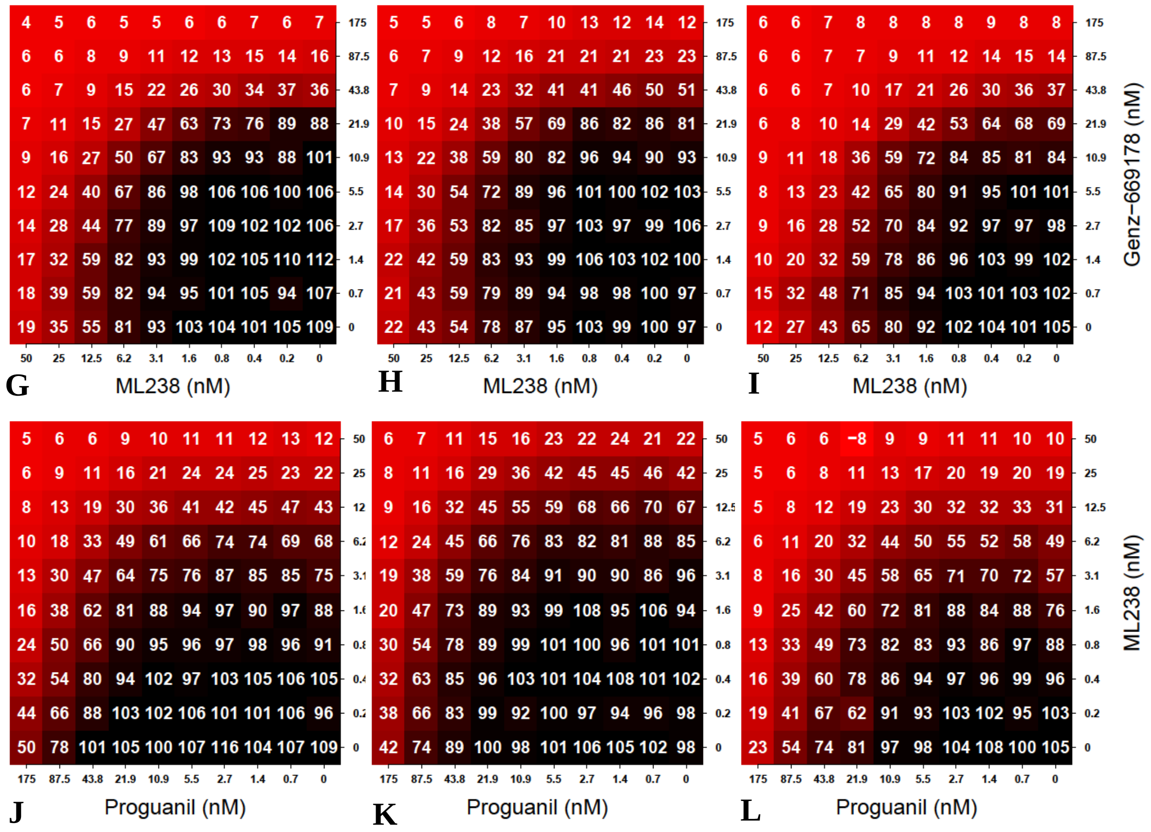

Fig. S8

Interaction heatmaps for ML238 + atovaquone against 3D7 (**A**, 1761/213), Dd2 (**B**, 1763/213) and Hb3 (**C**, 1764/213); ML238 + decoquinate against 3D7 (**D**, 1761/192), Dd2 (**E**, 1763/192) and Hb3 (**F**, 1764/192); ML238 + Genz-669178 against 3D7 (**G**, 1761/174), Dd2 (**H**, 1763/174) and Hb3 (**I**, 1764/174); ML238 + proguanil against 3D7 (**J**, 1761/210), Dd2 (**K**, 1763/210) and Hb3 (**L**, 1764/210). Numbers reflect the assay ID/serial for each heatmap as listed in the malaria folder at https://tripod.nih.gov/matrix-client/.

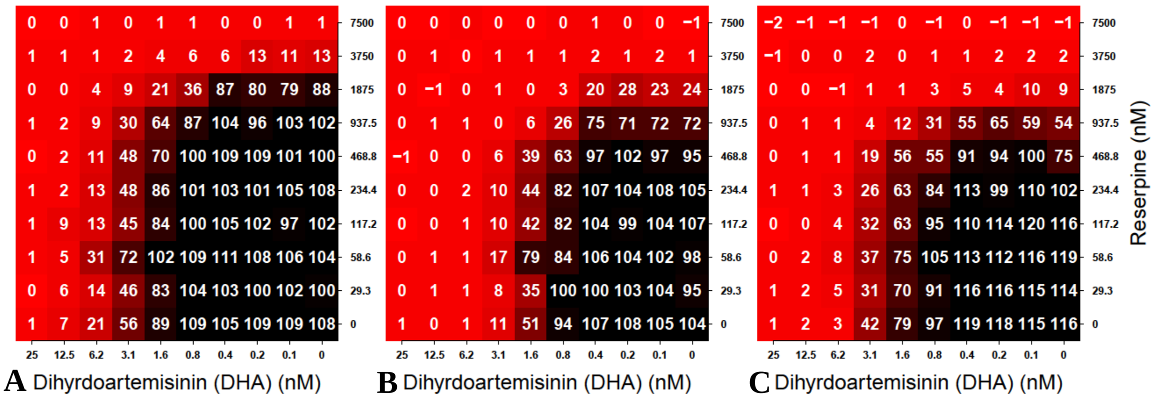

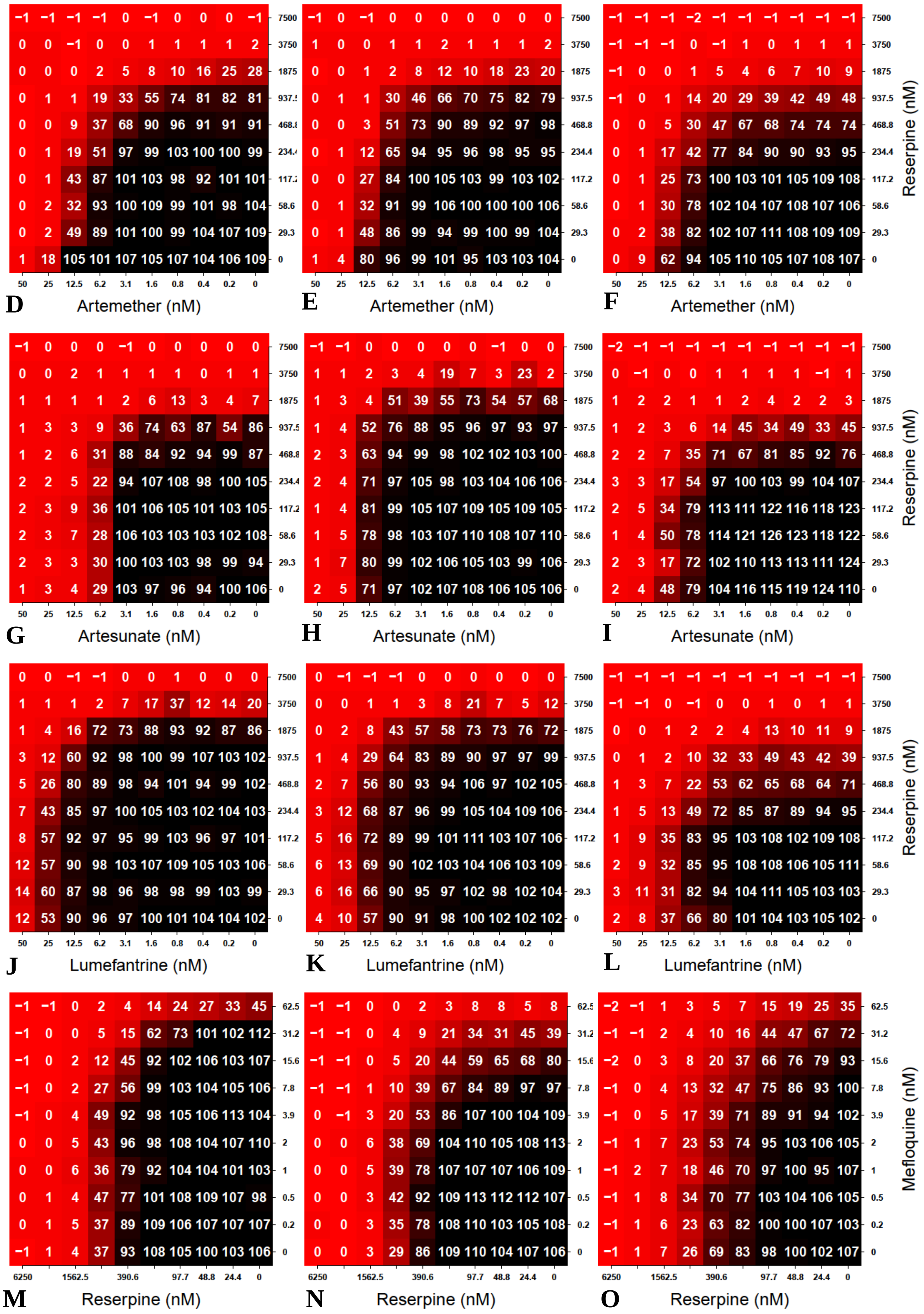
D
Artemether (nM)
E
Artemether (nM)
F
Artemether (nM)
Reserpine (nM)
G
Artesunate (nM)
H
Artesunate (nM)
I
Artesunate (nM)
Reserpine (nM)
J
Lumefantrine (nM)
K
Lumefantrine (nM)
L
Lumefantrine (nM)
Reserpine (nM)
M
Reserpine (nM)
N
Reserpine (nM)
O
Reserpine (nM)
Mefloquine (nM)

Fig. S9.1

Interaction heatmaps for reserpine + DHA against 3D7 (**A**, 1761/137), Dd2 (**B**, 1763/137) and Hb3 (**C**, 1764/137); reserpine + ATM against 3D7 (**D**, 1761/135), Dd2 (**E**, 1763/135) and Hb3 (**F**, 1764/135); reserpine + AS against 3D7 (**G**, 1761/136), Dd2 (**H**, 1763/136) and Hb3 (**I**, 1764/136); reserpine + LMF against 3D7 (**J**, 1761/138), Dd2 (**K**, 1763/138) and Hb3 (**L**, 1764/138); reserpine + MFQ against 3D7 (**M**, 502/69), Dd2 (**N**, 503/69) and Hb3 (**O**, 506/69). Numbers reflect the assay ID/serial for each heatmap as listed in the malaria folder at https://tripod.nih.gov/matrix-client/.

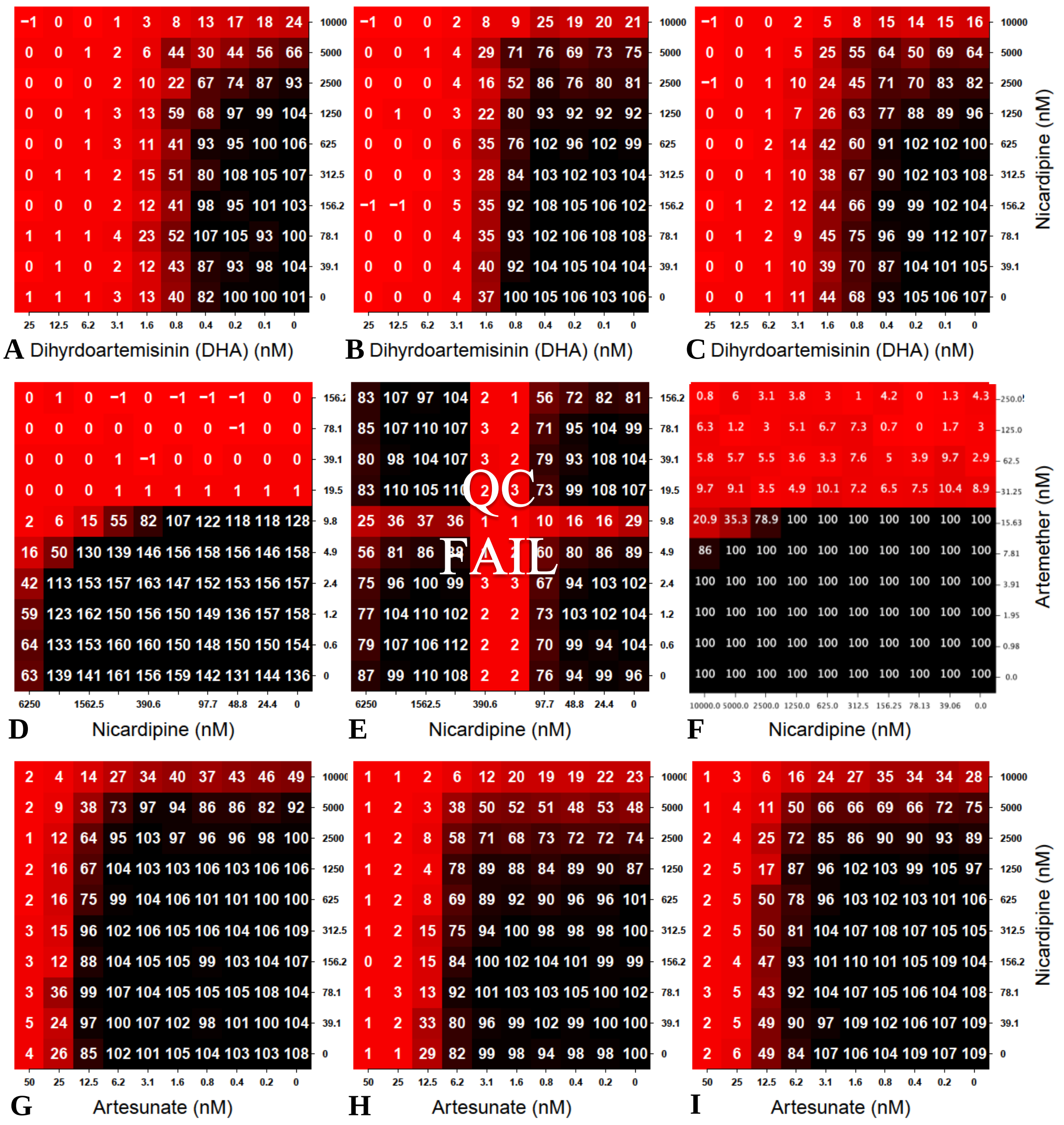

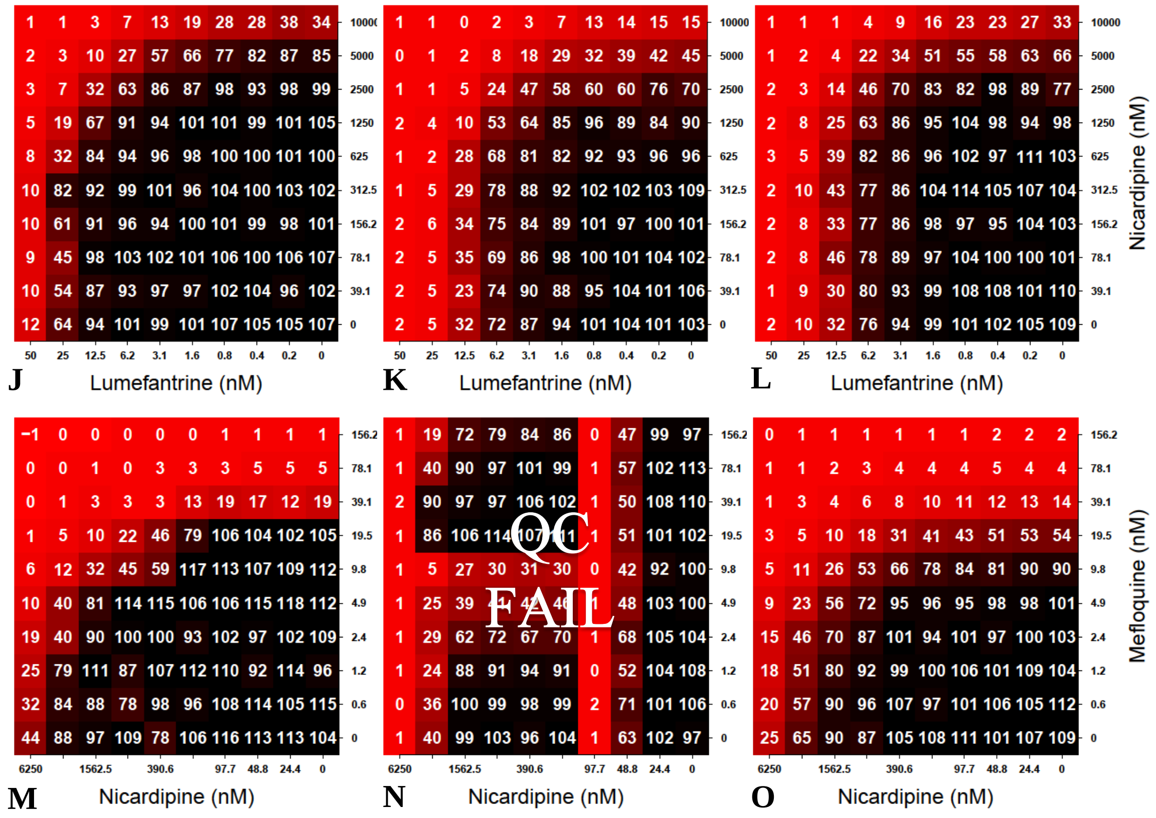

Fig. S9.2
Interaction heatmaps for nicardipine + DHA against 3D7 (**A**, 1761/88), Dd2 (**B**, 1763/88) and Hb3 (**C**, 1764/88); nicardipine + ATM against 3D7 (**D**, 723/45), Dd2 (**E**, 725/45 – in this case, the assay failed. The assay was repeated and can be found at 824/175) and Hb3 (**F**, 724/45); nicardipine + AS against 3D7 (**G**, 1761/87), Dd2 (**H**, 1763/87) and Hb3 (**I**, 1764/87); nicardipine + LMF against 3D7 (**J**, 1761/89), Dd2 (**K**, 1763/89) and Hb3 (**L**, 1764/89); nicardipine + MFQ against 3D7 (**M**, 723/63), Dd2 (**N**, 725/63) and Hb3 (**O**, 724/63 – in this case, the assay failed. The assay was repeated and can be found at 824/178). Numbers reflect the assay ID/serial for each heatmap as listed in the malaria folder at https://tripod.nih.gov/matrix-client/.

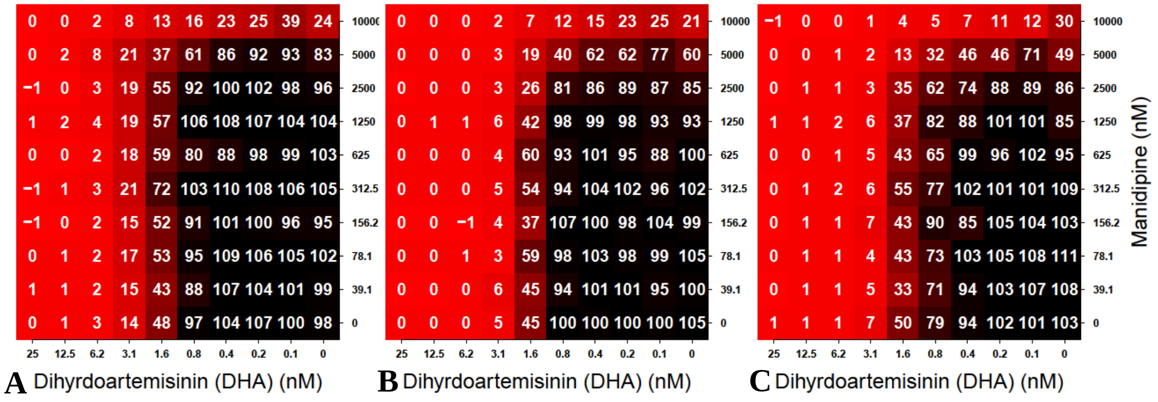

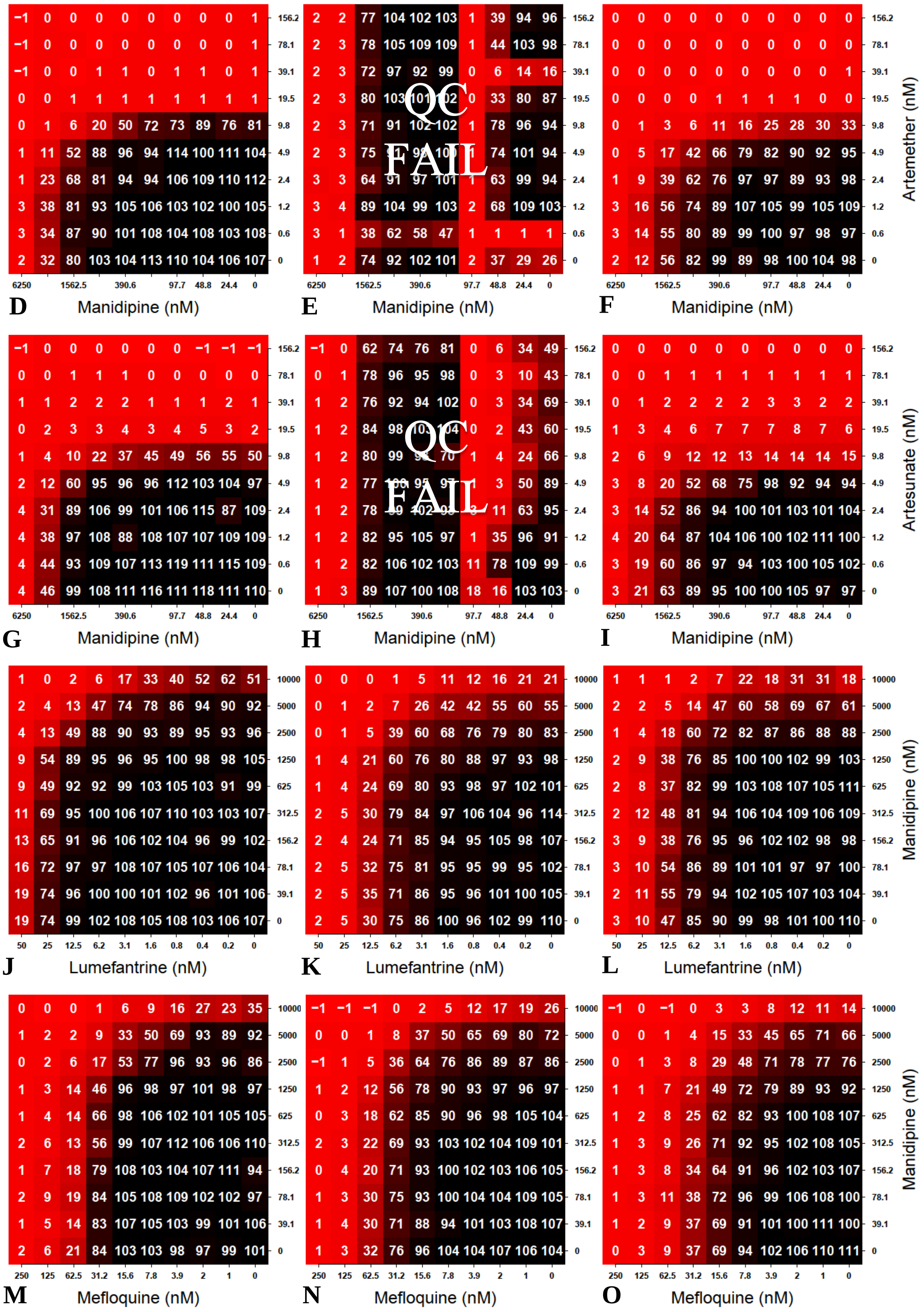
D
Manidipine (nM)
E
QC FAIL
Manidipine (nM)
F
Manidipine (nM)
Artemether (nM)
G
Manidipine (nM)
H
QC FAIL
Manidipine (nM)
I
Manidipine (nM)
Artesunate (nM)
J
Lumefantrine (nM)
K
Lumefantrine (nM)
L
Lumefantrine (nM)
Manidipine (nM)
M
Mefloquine (nM)
N
Mefloquine (nM)
O
Mefloquine (nM)
Manidipine (nM)

Fig. S9.3

Interaction heatmaps for manidipine + DHA against 3D7 (**A**, 1761/82), Dd2 (**B**, 1763/82) and Hb3 (**C**, 1764/82); manidipine + ATM against 3D7 (**D**, 723/50), Dd2 (**E**, 725/50 – in this case, the assay failed. The assay was repeated and can be found at 824/180) and Hb3 (**F**, 724/50); manidipine + AS against 3D7 (**G**, 723/82), Dd2 (**H**, 725/82 – in this case, the assay failed. The assay was repeated and can be found at 824/182) and Hb3 (**I**, 724/82); manidipine + LMF against 3D7 (**J**, 1761/83), Dd2 (**K**, 1763/83) and Hb3 (**L**, 1764/83) ; manidipine + MFQ against 3D7 (**M**, 1761/85), Dd2 (**N**, 1763/85) and Hb3 (**O**, 1764/85). Numbers reflect the assay ID/serial for each heatmap as listed in the malaria folder at https://tripod.nih.gov/matrix-client/.

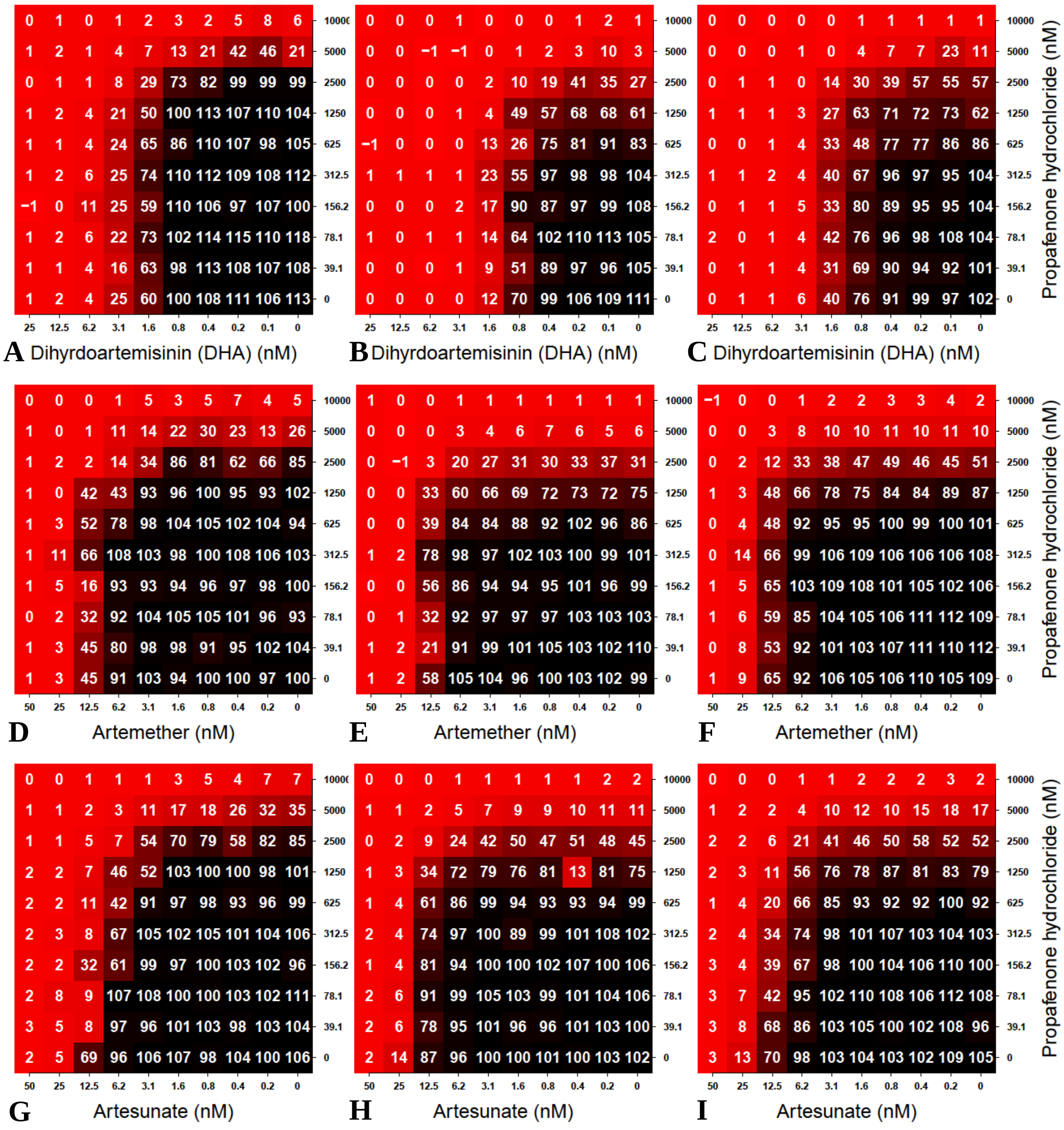

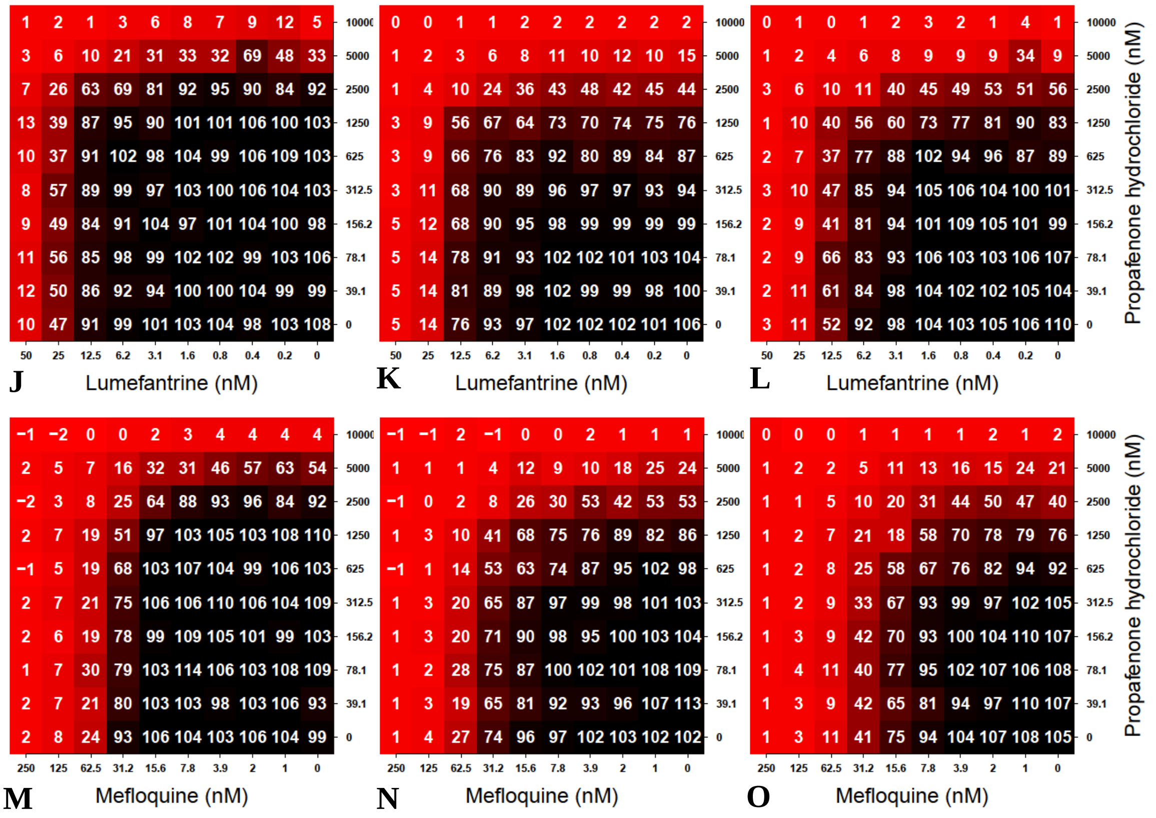

Fig. S9.4
Interaction heatmaps for propafenone + DHA against 3D7 (**A**, 1761/106), Dd2 (**B**, 1763/106) and Hb3 (**C**, 1764/106); propafenone + ATM against 3D7 (**D**, 1761/104), Dd2 (**E**, 1763/104) and Hb3 (**F**, 1764/104); propafenone + AS against 3D7 (**G**, 1761/105), Dd2 (**H**, 1763/105) and Hb3 (**I**, 1764/105); propafenone + LMF against 3D7 (**J**, 1761/107), Dd2 (**K**, 1763/107) and Hb3 (**L**, 1764/107); propafenone + MFQ against 3D7 (**M**, 1761/109), Dd2 (**N**, 1763/109) and Hb3 (**O**, 1764/109). Numbers reflect the assay ID/serial for each heatmap as listed in the malaria folder at https://tripod.nih.gov/matrix-client/.

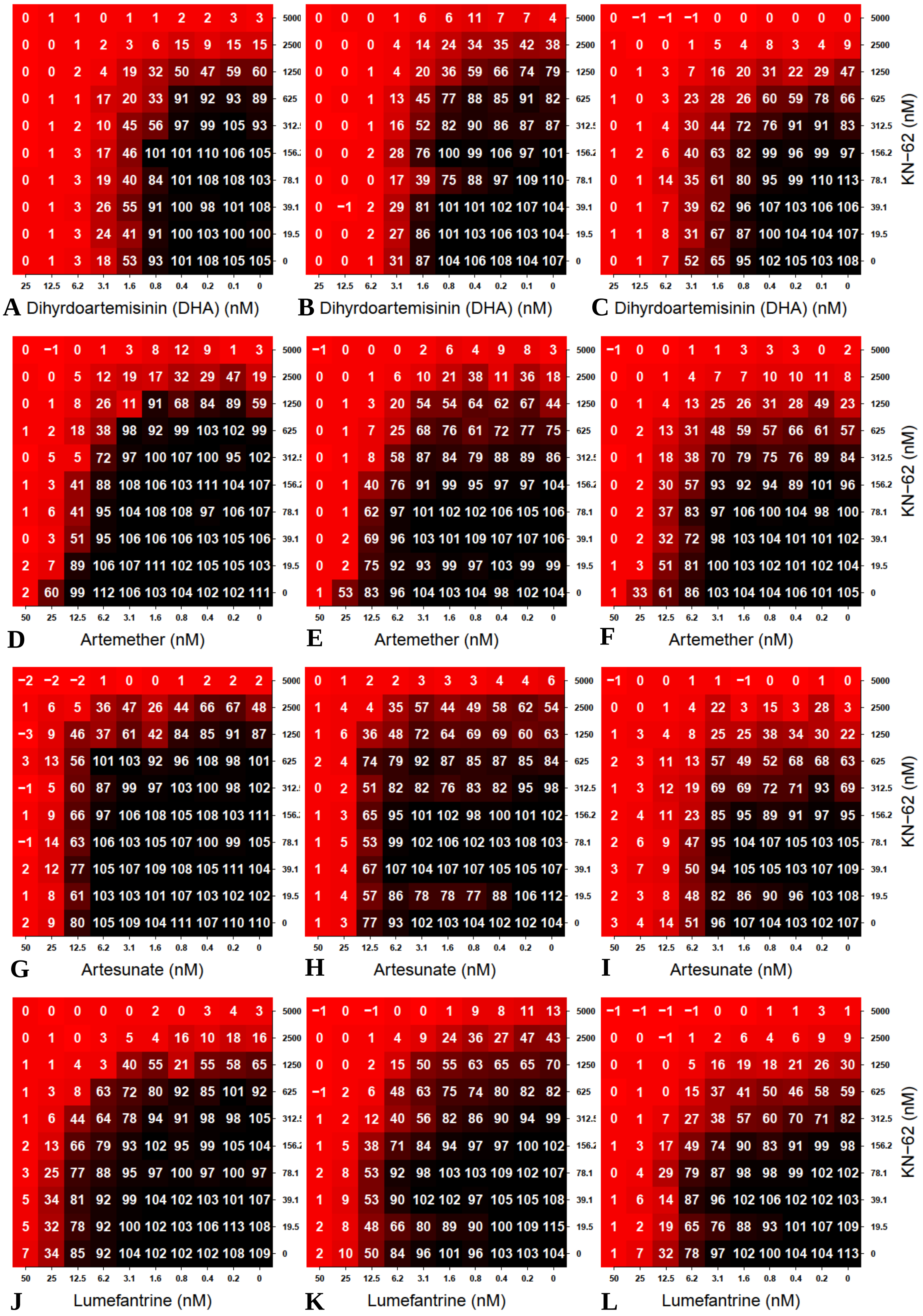
A Dihyrdoartemisinin (DHA) (nM)
B Dihyrdoartemisinin (DHA) (nM)
C Dihyrdoartemisinin (DHA) (nM)
D Artemether (nM)
E Artemether (nM)
F Artemether (nM)
G Artesunate (nM)
H Artesunate (nM)
I Artesunate (nM)
J Lumefantrine (nM)
K Lumefantrine (nM)
L Lumefantrine (nM)
KN−62 (nM)
KN−62 (nM)
KN−62 (nM)
KN−62 (nM)

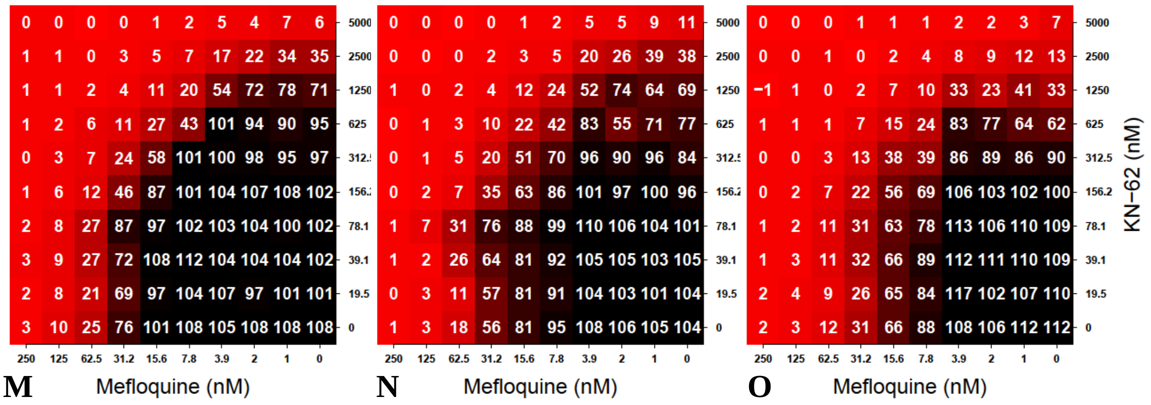

Fig. S9.5
Interaction heatmaps for KN-62 + DHA against 3D7 (**A**, 1761/182), Dd2 (**B**, 1763/182) and Hb3 (**C**, 1764/182); KN-62 + ATM against 3D7 (**D**, 1761/180), Dd2 (**E**, 1763/180) and Hb3 (**F**, 1764/180); KN-62 + AS against 3D7 (**G**, 1761/181), Dd2 (**H**, 1763/181) and Hb3 (**I**, 1764/181); KN-62 + LMF against 3D7 (**J**, 1761/183), Dd2 (**K**, 1763/183) and Hb3 (**L**, 1764/183); KN-62 + MFQ against 3D7 (**M**, 1761/185), Dd2 (**N**, 1763/185) and Hb3 (**O**, 1764/185). Numbers reflect the assay ID/serial for each heatmap as listed in the malaria folder at https://tripod.nih.gov/matrix-client/.

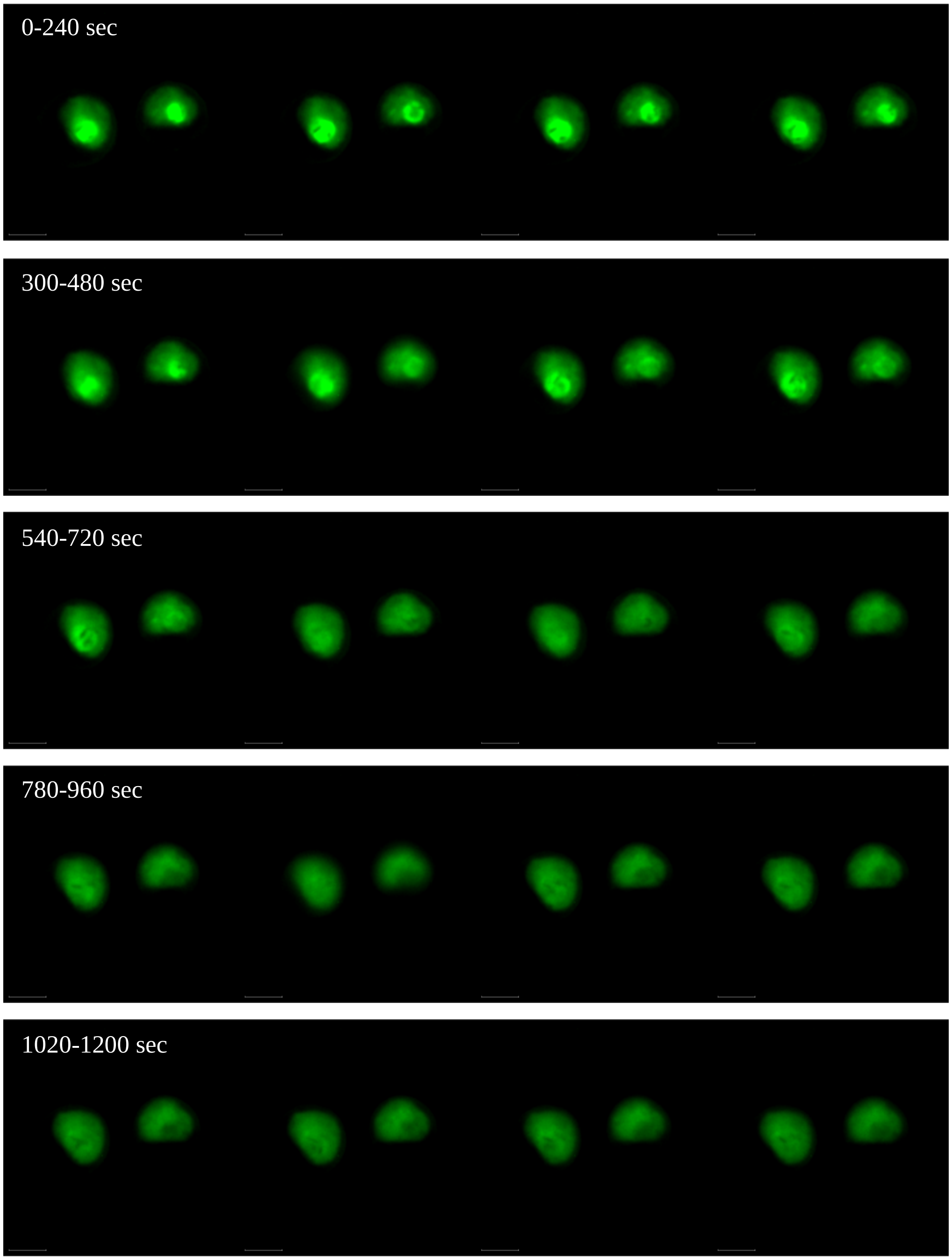

Fig. S10
Time lapsed capture of calcium dependent Fura - 2 fluorescence for two live side - by - side intraerythrocytic strain Dd2 parasites showing rapid loss of DV Ca^{2+} (bright green inner circle, top panel 0 - 240 sec) upon perfusion with cytocidal ($2 \times LD_{50}$) dose of CQ (see methods).

3D7 Summary JC1 Assays

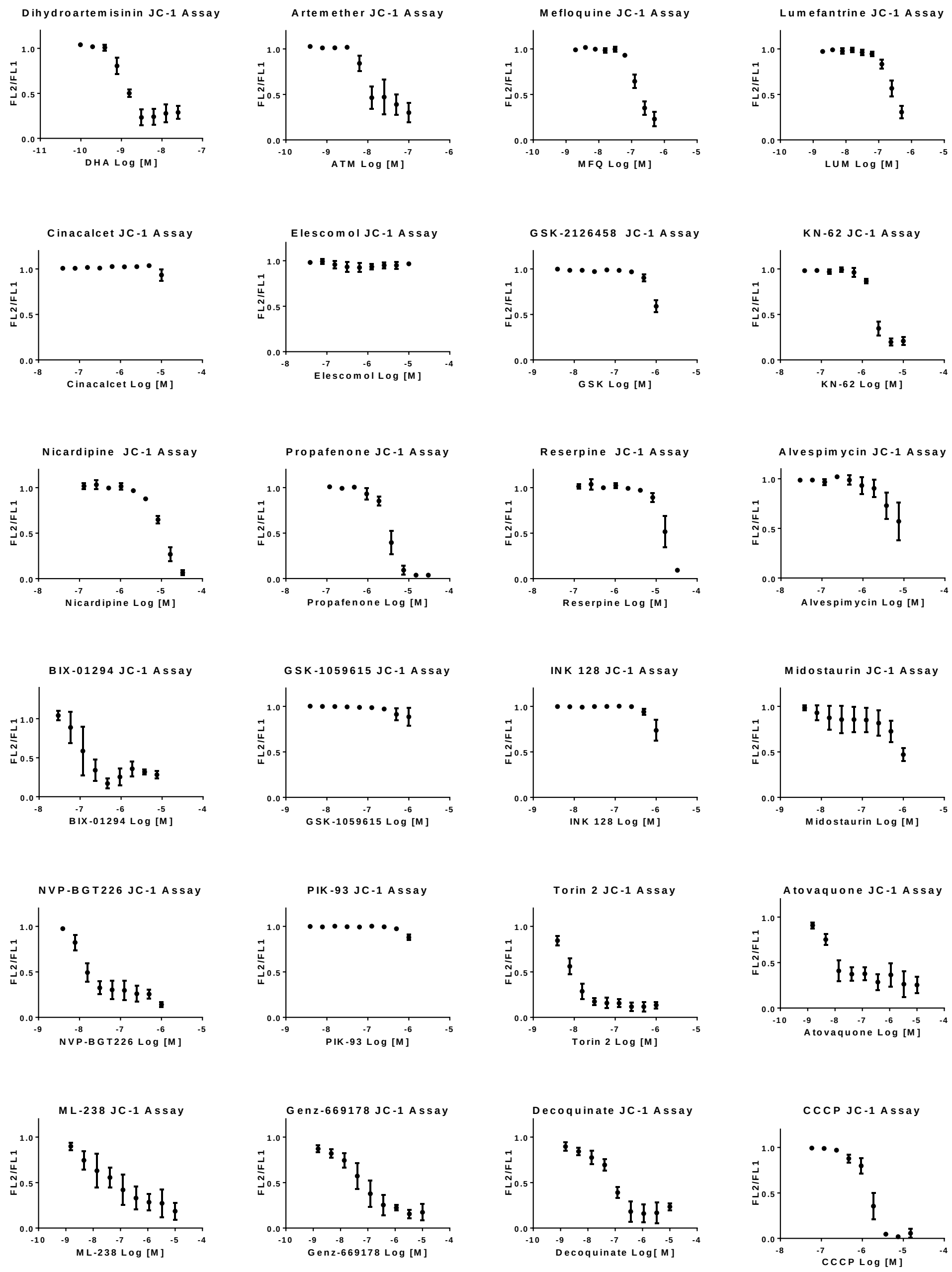

Fig S11.1

Compound effect on *P. falciparum* 3D7 mitochondrial transmembrane potential as assessed by JC1 staining. Evaluation of each compounds ability to perturb the mitochondrial membrane potential in a dose dependent manner. Graphs indicate the dose dependent $\Delta\Psi$ dissipation expressed as FL2/FL1 (Red/green fluorescence ratio). Each value has been normalized versus the FL2/FL1 ratio of the vehicle treated control to which an arbitrary value of 1 has been assigned. Each point is the mean of three independent experiments performed in duplicate, error bars represent SEM.

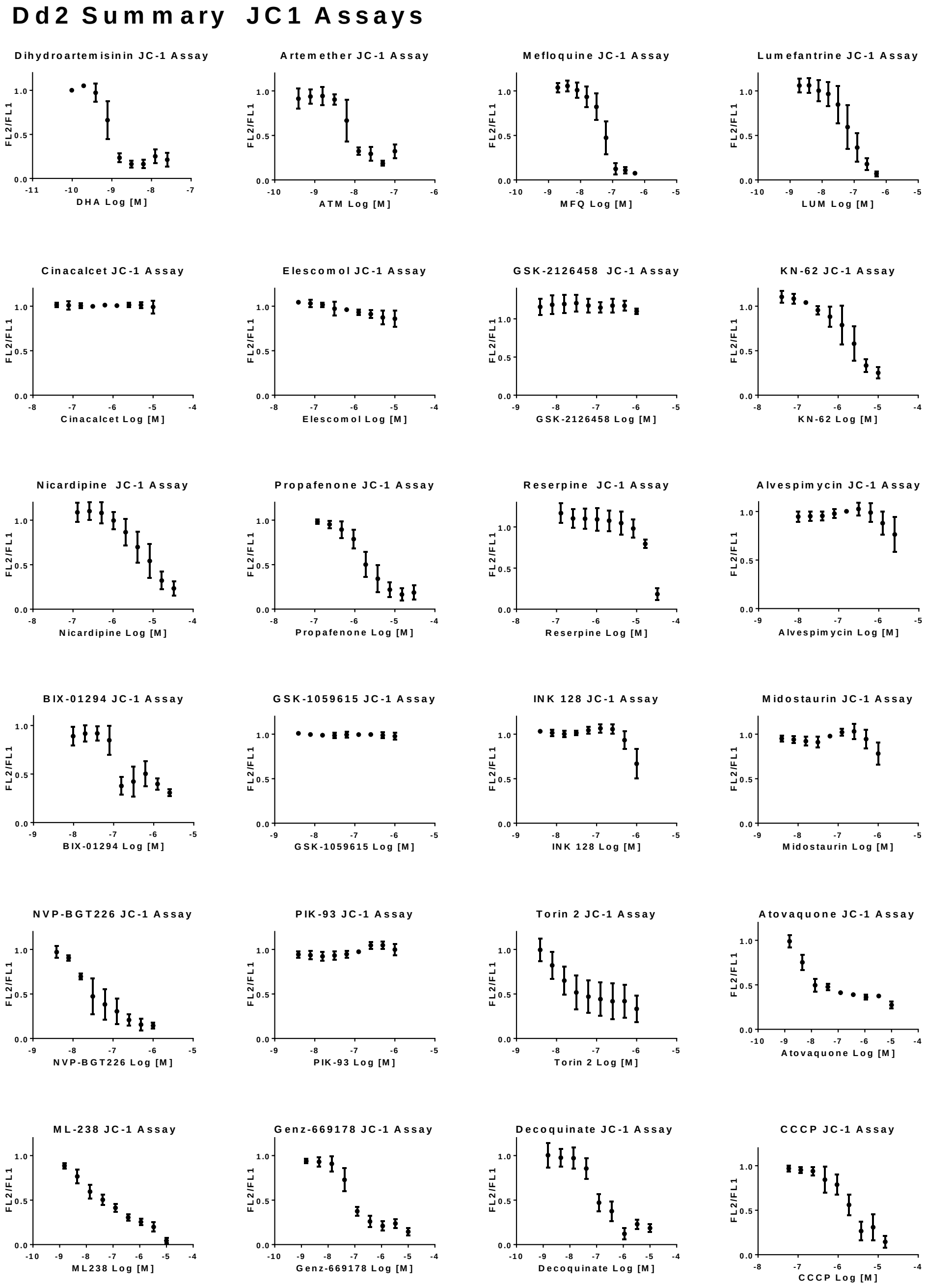

Fig S11.2

Compound effect on *P. falciparum* Dd2 mitochondrial transmembrane potential as assessed by JC1 staining. Evaluation of each compounds ability to perturb the mitochondrial membrane potential in a dose dependent manner. Graphs indicate the dose dependent ΔΨ dissipation expressed as FL2/FL1 (Red/green fluorescence ratio). Each value has been normalized versus the FL2/FL1 ratio of the vehicle treated control to which an arbitrary value of 1 has been assigned. Each point is the mean of three independent experiments performed in duplicate, error bars represent SEM.

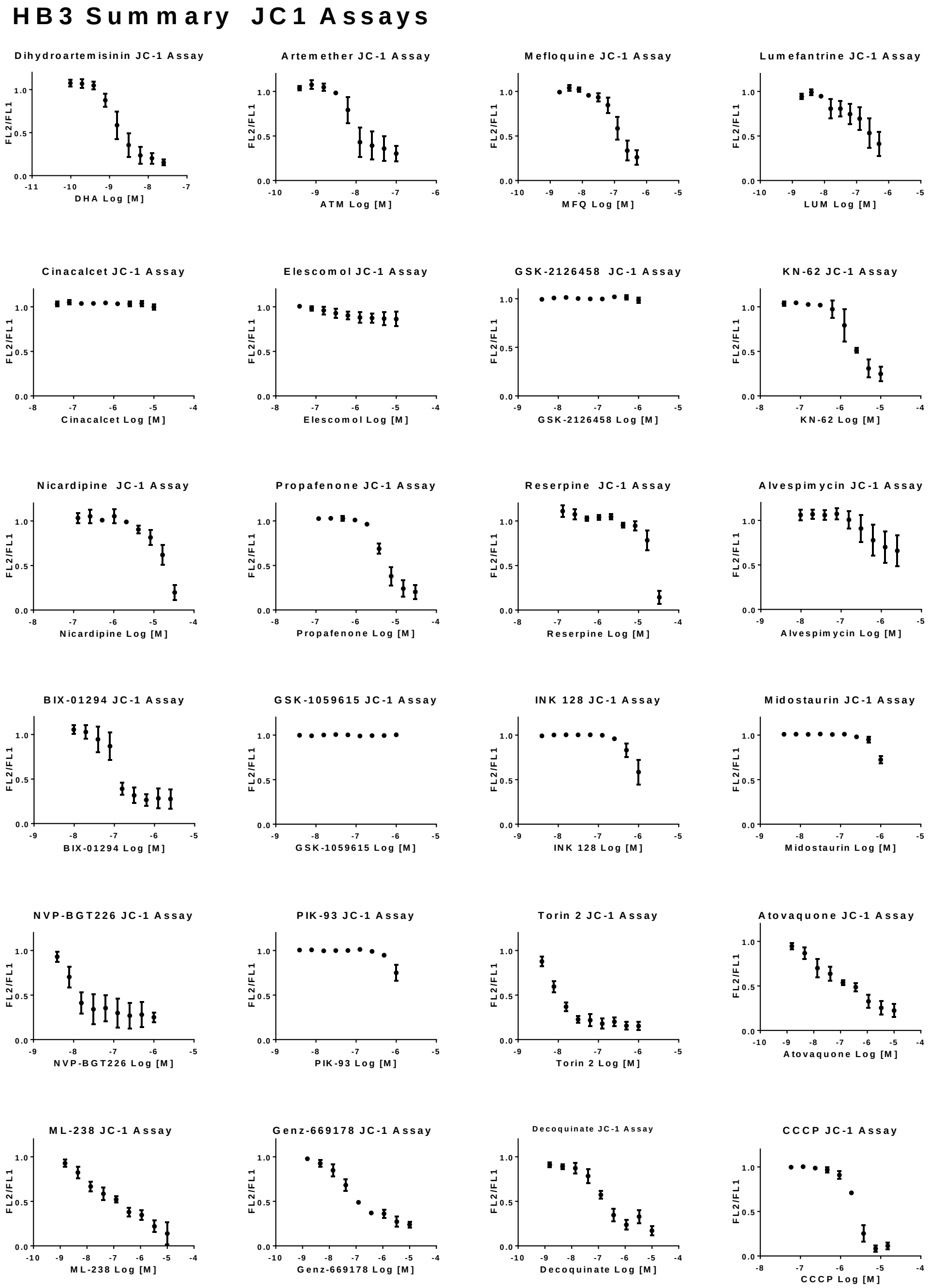

Fig S11.3

Compound effect on *P. falciparum* HB3 mitochondrial transmembrane potential as assessed by JC1 staining. Evaluation of each compounds ability to perturb the mitochondrial membrane potential in a dose dependent manner. Graphs indicate the dose dependent $\Delta\Psi$ dissipation expressed as FL2/FL1 (Red/green fluorescence ratio). Each value has been normalized versus the FL2/FL1 ratio of the vehicle treated control to which an arbitrary value of 1 has been assigned. Each point is the mean of three independent experiments performed in duplicate, error bars represent SEM.

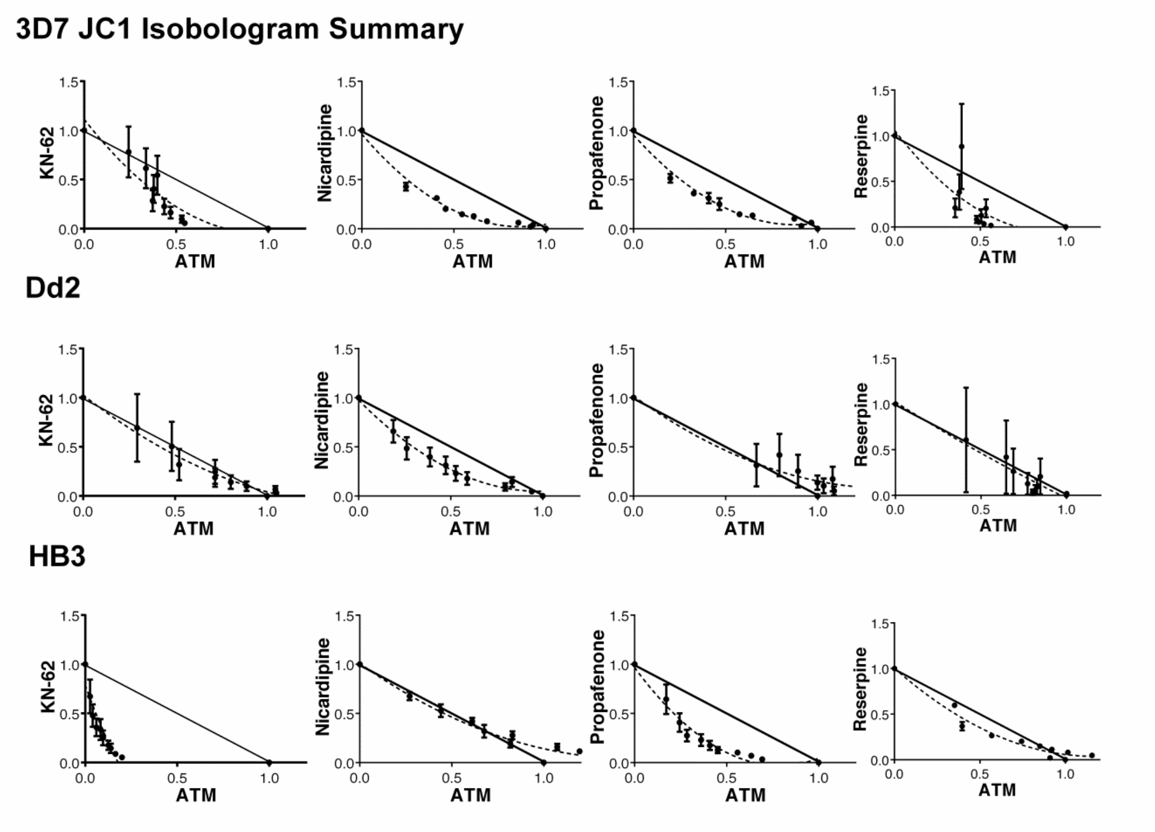

Fig. S11.4

Compound Interaction with Artemether on *P. falciparum* mitochondrial transmembrane potential as assessed by JC1 staining. Evaluation of each compounds interaction with Artemether in modulating the mitochondrial membrane potential in a fixed ratio assay. Graphs indicate the fixed ratio dependent $\Delta\Psi$ dissipation expressed as FL2/FL1 (Red/green fluorescence ratio). Each value has been normalized versus the FL2/FL1 ratio of the vehicle treated control to which an arbitrary value of 1 has been assigned. Each point is the mean of three independent experiments performed, error bars represent SEM.

3D7 Summary ROS Assays

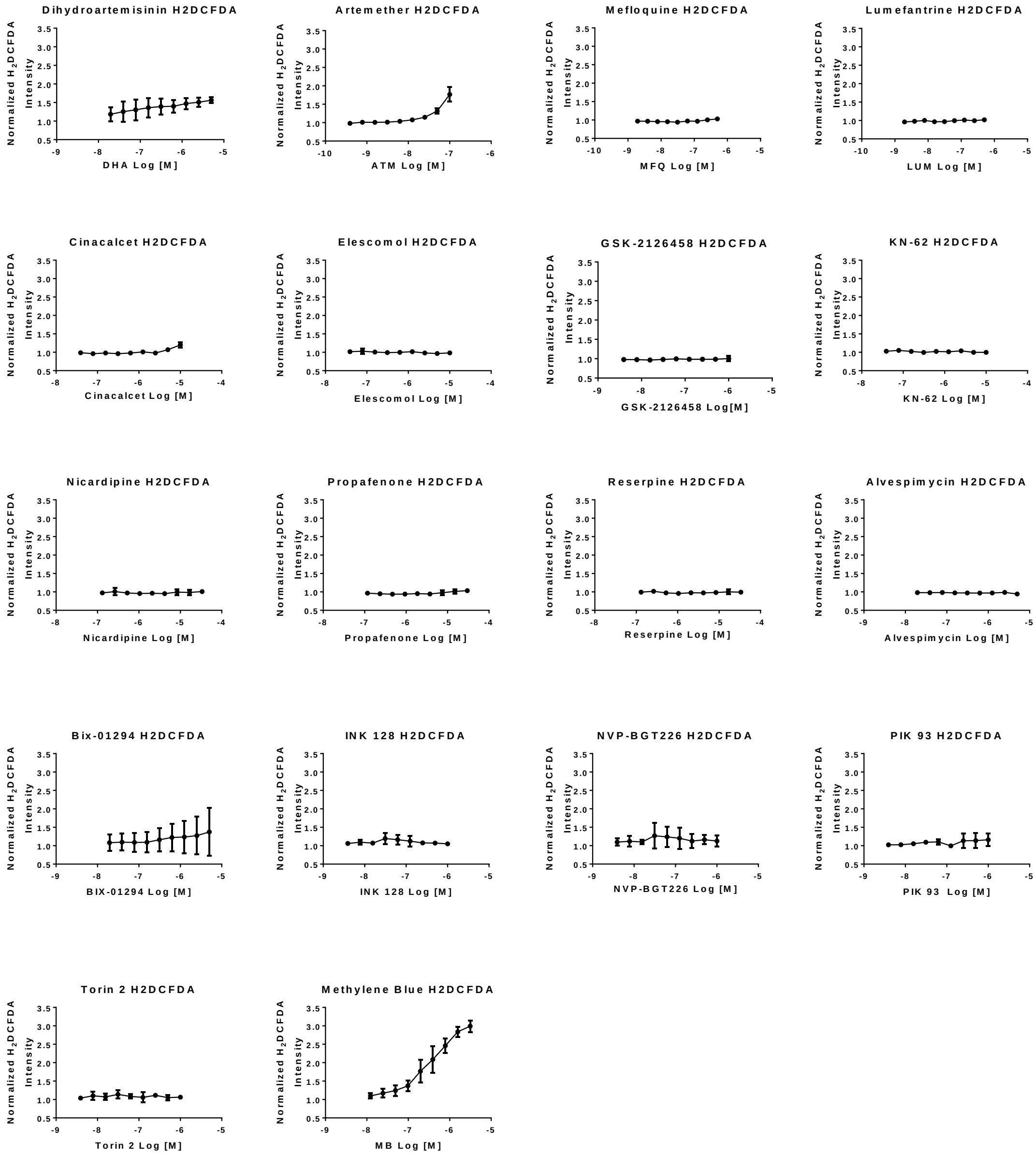

Fig S12.1

Assessment of compound pro-oxidant activity in *Plasmodium falciparum* 3D7. Synchronized trophozoite *P. falciparum* 3D7 parasites labeled with H_2DCFDA were incubated for 3 h in the presence of serially diluted compound. Cells were then washed and the mean DCF signal of the parasite infected erythrocyte population was analyzed by flow cytometry. DCF signal was normalized to untreated controls; error bars represent SEM of duplicate measurements from at least three independent assays.

Dd2 Summary ROS Assays

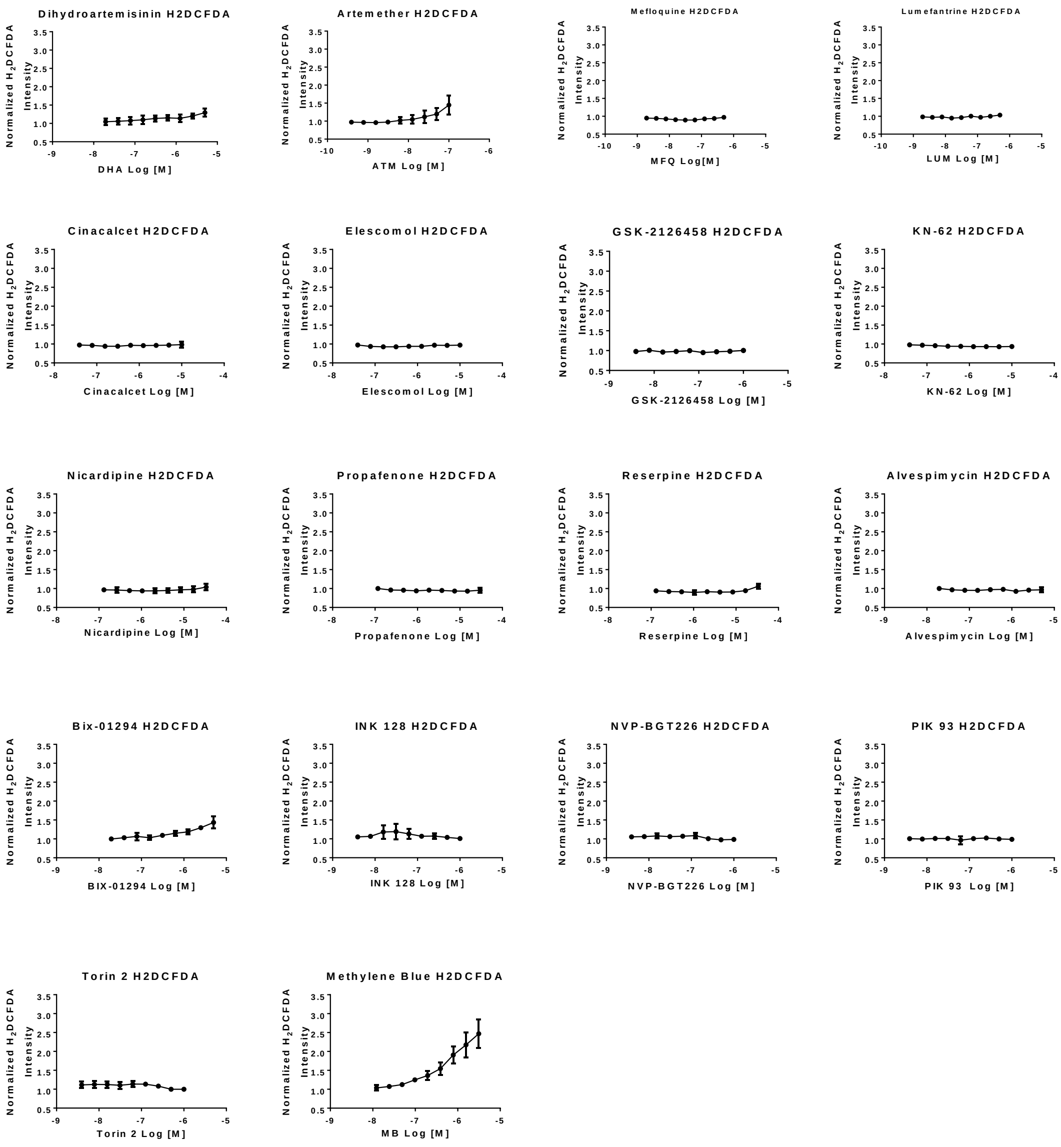

Fig S12.2

Assessment of compound pro-oxidant activity in *Plasmodium falciparum* Dd2. Synchronized trophozoite *P. falciparum* Dd2 parasites labeled with H_2DCFDA were incubated for 3 h in the presence of serially diluted compound. Cells were then washed and the mean DCF signal of the parasite infected erythrocyte population was analyzed by flow cytometry. DCF signal was normalized to untreated controls; error bars represent SEM of duplicate measurements from at least three independent assays.

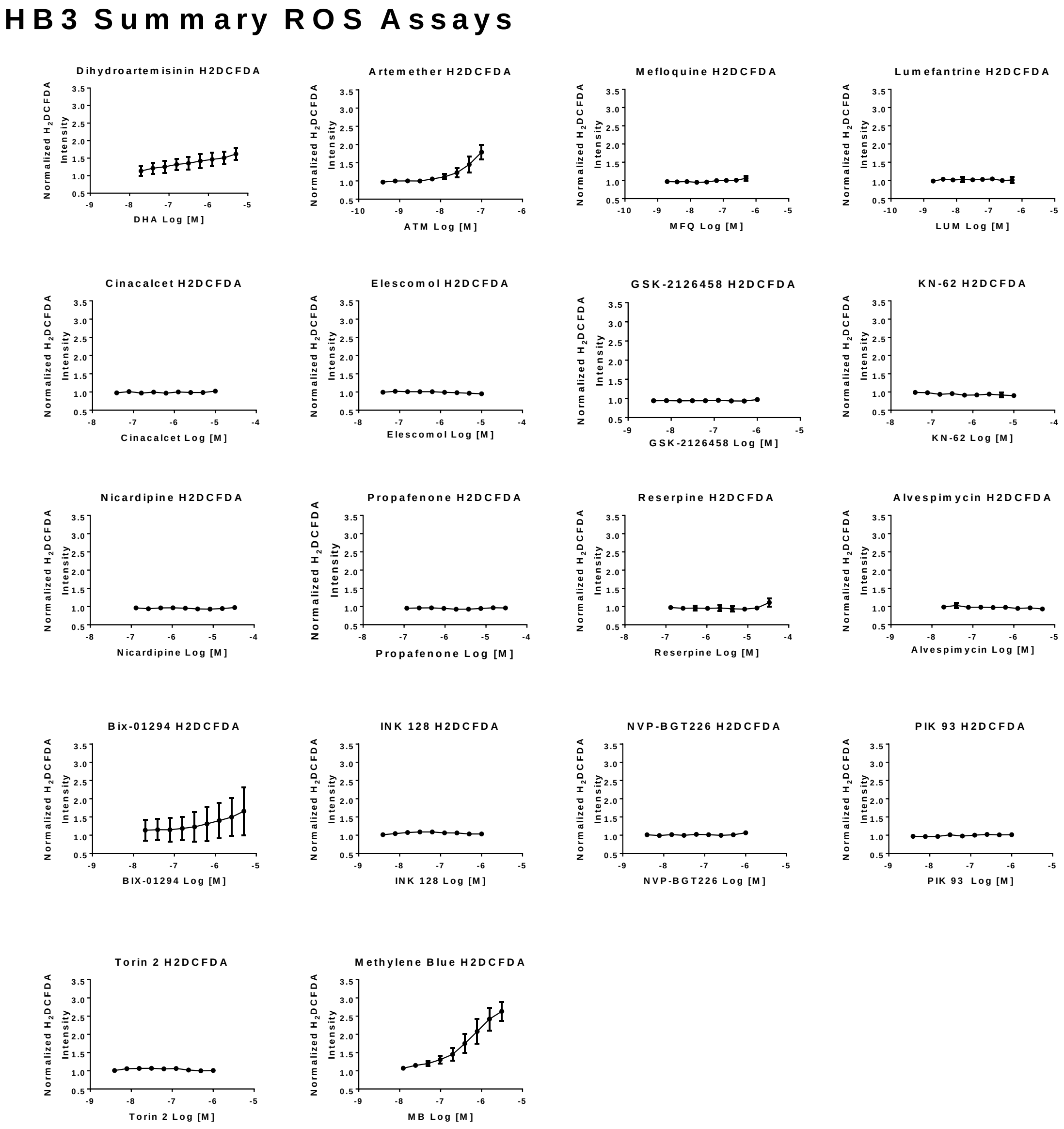

Fig S12.3

Assessment of compound pro-oxidant activity in *Plasmodium falciparum* HB3. Synchronized trophozoite *P. falciparum* HB3 parasites labeled with H_2DCFDA were incubated for 3 h in the presence of serially diluted compound. Cells were then washed and the mean DCF signal of the parasite infected erythrocyte population was analyzed by flow cytometry. DCF signal was normalized to untreated controls; error bars represent SEM of duplicate measurements from at least three independent assays.

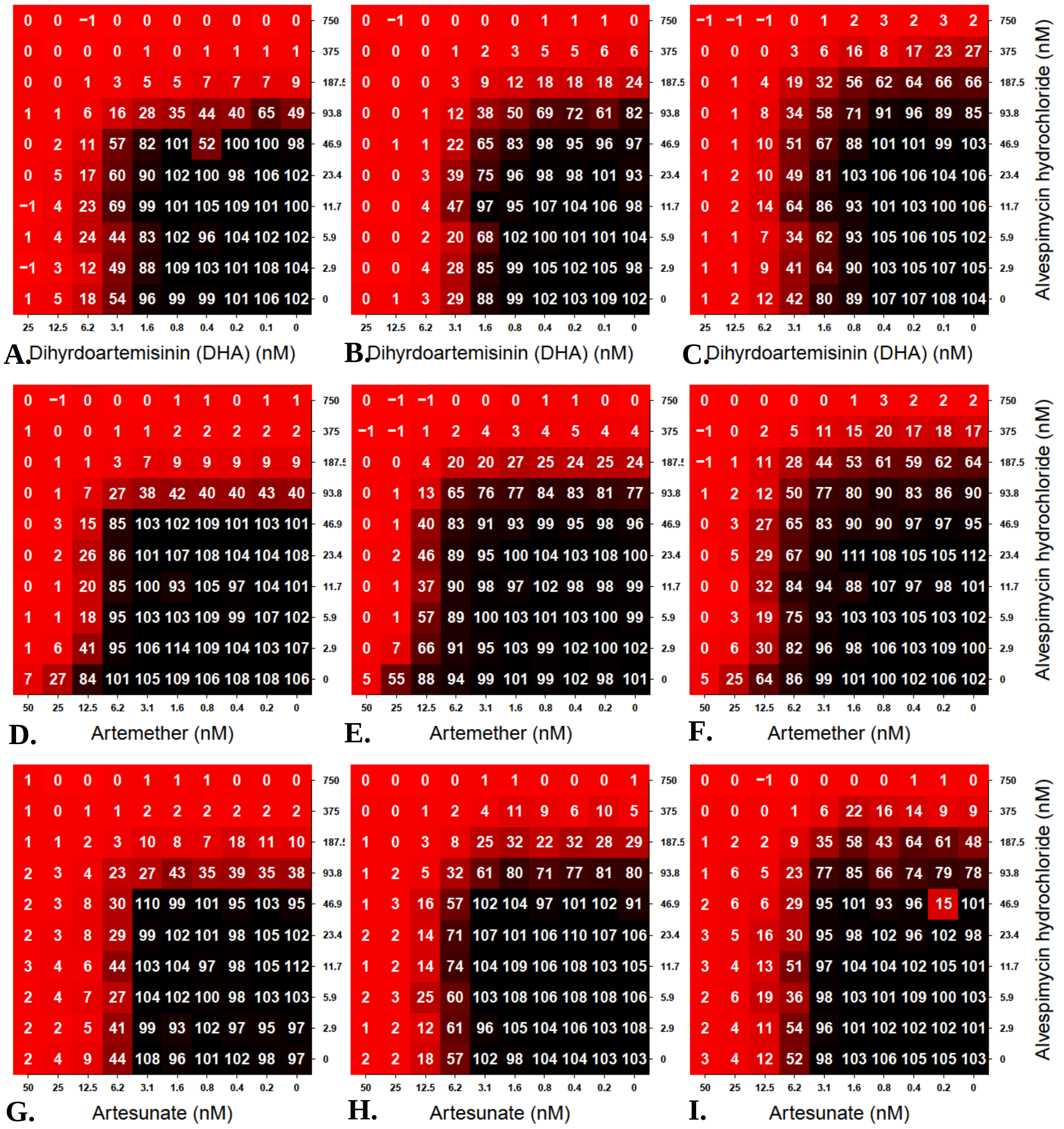

Fig. S13.1
Interaction heatmaps for alvespimycin + DHA against 3D7 (**A**, 1761/152), Dd2 (**B**, 1763/152) and Hb3 (**C**, 1764/152); alvespimycin + ATM against 3D7 (**D**, 1761/150), Dd2 (**E**, 1763/150) and Hb3 (**F**, 1764/150); alvespimycin + AS against 3D7 (**G**, 1761/151), Dd2 (**H**, 1763/151) and Hb3 (**I**, 1764/151). Numbers reflect the assay ID/serial for each heatmap as listed in the malaria folder at https://tripod.nih.gov/matrix-client/.

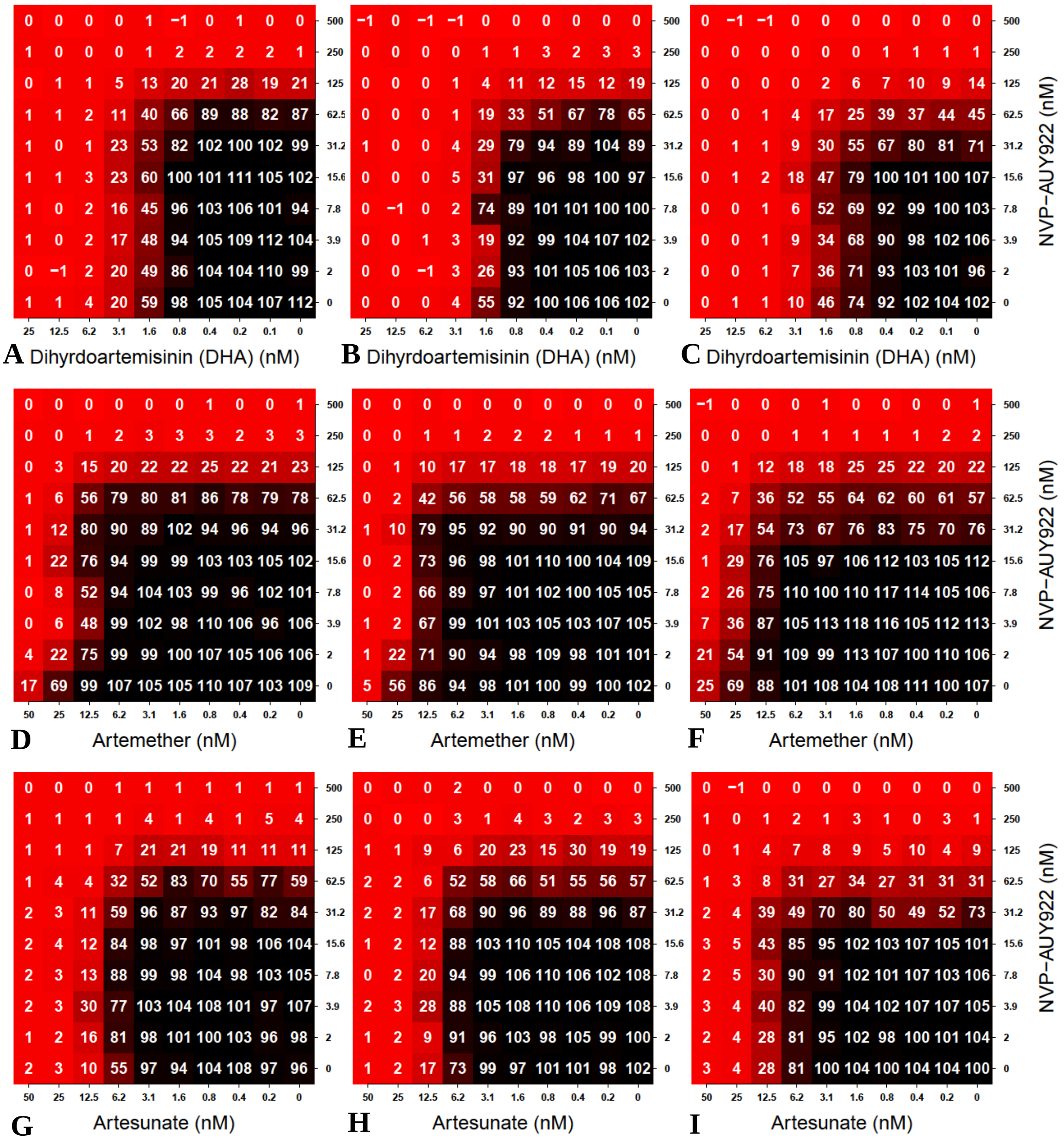

Fig. S13.2

Interaction heatmaps for NVP-AUY922 + DHA against 3D7 (**A**, 1761/68), Dd2 (**B**, 1763/68) and Hb3 (**C**, 1764/68); NVP-AUY922 + ATM against 3D7 (**D**, 1761/66), Dd2 (**E**, 1763/66) and Hb3 (**F**, 1764/66); NVP-AUY922 + AS against 3D7 (**G**, 1761/67), Dd2 (**H**, 1763/67) and Hb3 (**I**, 1764/67). Numbers reflect the assay ID/serial for each heatmap as listed in the malaria folder at https://tripod.nih.gov/matrix-client/.

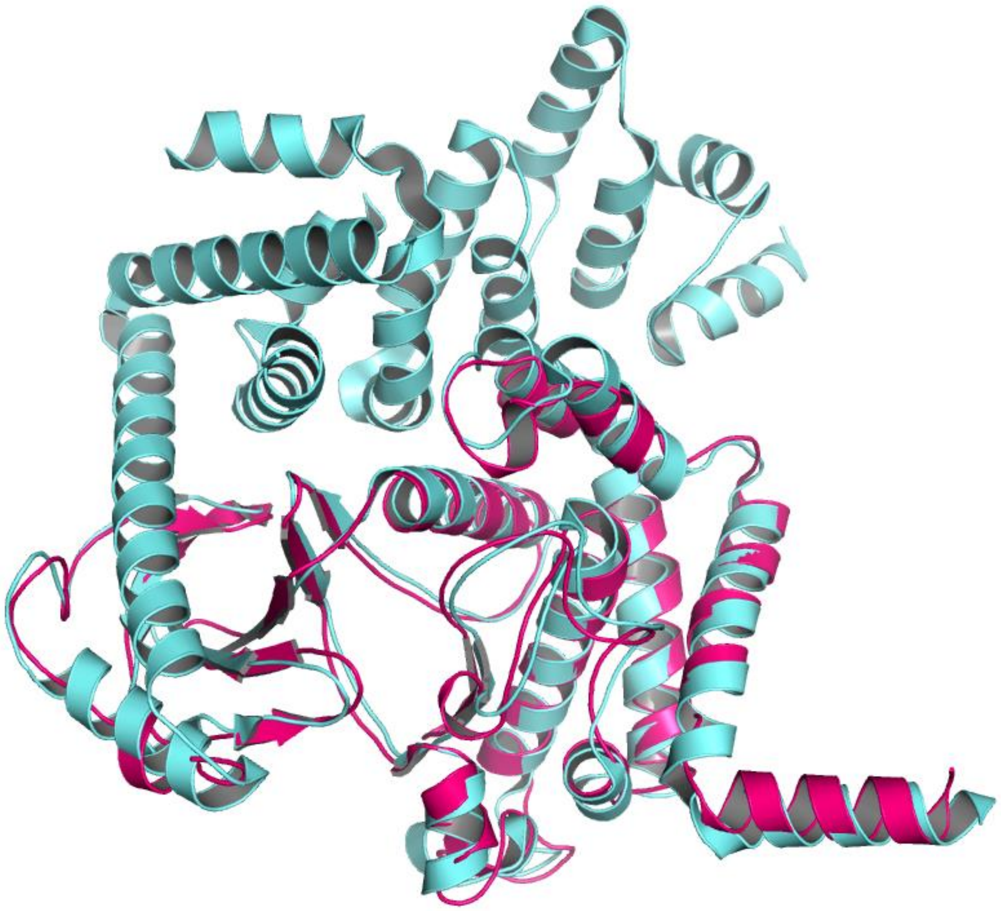

A

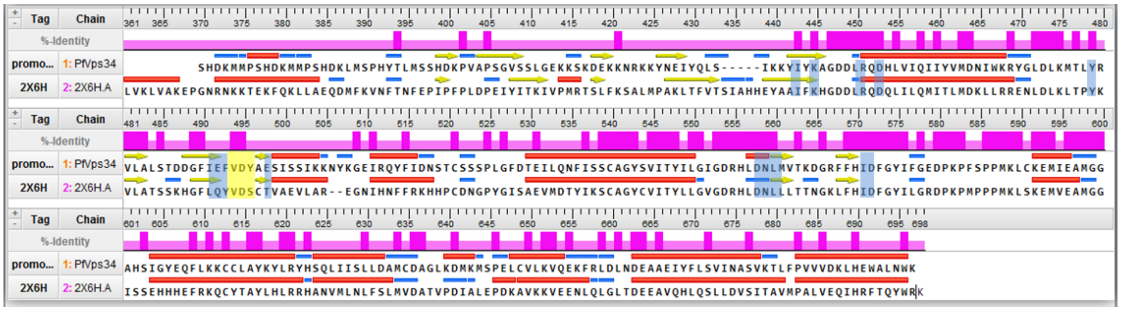

B

Fig. S14

Homology model of PfVps34 (colored in purple) overlaid with dmPI3K template (PDB ID: 2X6H) (**A**). Sequence alignment of PfVps34 and DmVps34, showing 12 of 18 conserved amino acids (highlighted in yellow for hinge region residues and light blue for the rest) in the ligand binding region (**B**).

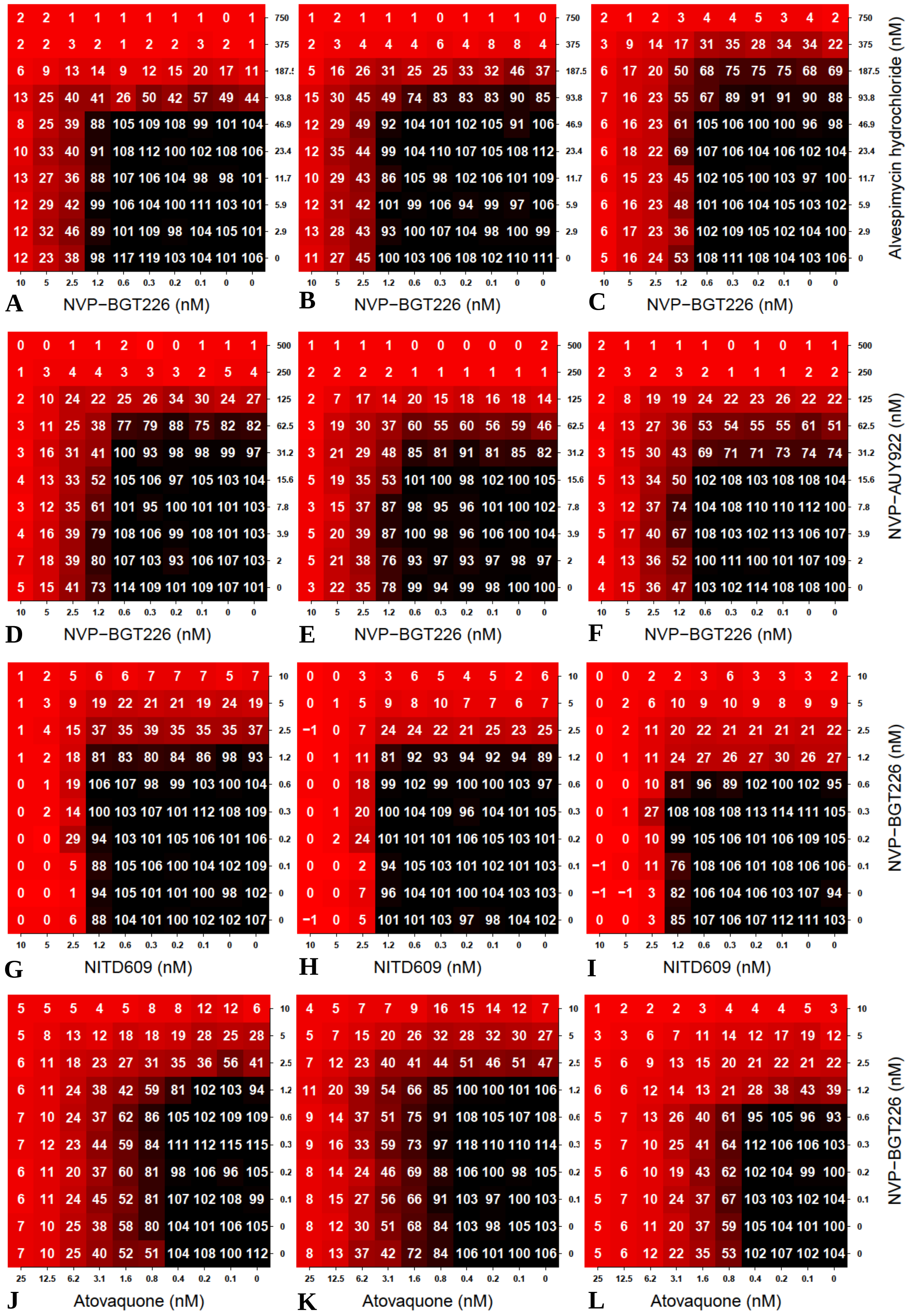
A
2 2 1 1 1 1 1 1 0 1 750
2 2 3 2 1 2 2 3 2 1 375
6 9 13 14 9 12 15 20 17 11 187.5
13 25 40 41 26 50 42 57 49 44 93.8
8 25 39 88 105 109 108 99 101 104 46.9
10 33 40 91 108 112 100 102 108 106 23.4
13 27 36 88 107 106 104 98 98 101 11.7
12 29 42 99 106 104 100 111 103 101 5.9
12 32 46 89 101 109 98 104 105 101 2.9
12 23 38 98 117 119 103 104 101 106 0
10 5 2.5 1.2 0.6 0.3 0.2 0.1 0 0
NVP−BGT226 (nM)
B
1 2 1 1 1 0 1 1 1 0 750
2 3 4 4 4 6 4 8 8 4 375
5 16 26 31 25 25 33 32 46 37 187.5
15 30 45 49 74 83 83 83 90 85 93.8
12 29 49 92 104 101 102 105 91 106 46.9
12 35 44 99 104 110 107 105 108 112 23.4
10 29 43 86 105 98 102 106 101 109 11.7
12 31 42 101 99 106 94 99 97 106 5.9
13 28 43 93 100 107 104 98 100 99 2.9
11 27 45 100 103 106 108 102 110 111 0
10 5 2.5 1.2 0.6 0.3 0.2 0.1 0 0
NVP−BGT226 (nM)
C
2 1 2 3 4 4 5 3 4 2 750
3 9 14 17 31 35 28 34 34 22 375
6 17 20 50 68 75 75 75 68 69 187.5
7 16 23 55 67 89 91 91 90 88 93.8
6 16 23 61 105 106 100 100 96 98 46.9
6 18 22 69 107 106 104 106 102 104 23.4
6 15 23 45 102 105 100 103 97 100 11.7
6 16 23 48 101 106 104 105 103 102 5.9
6 17 23 36 102 109 105 102 104 100 2.9
5 16 24 53 108 111 108 104 103 106 0
10 5 2.5 1.2 0.6 0.3 0.2 0.1 0 0
NVP−BGT226 (nM)
Alvespimycin hydrochloride (nM)
D
0 0 1 1 2 0 0 1 1 1 500
1 3 4 4 3 3 3 2 5 4 250
2 10 24 22 25 26 34 30 24 27 125
3 11 25 38 77 79 88 75 82 82 62.5
3 16 31 41 100 93 98 98 99 97 31.2
4 13 33 52 105 106 97 105 103 104 15.6
3 12 35 61 101 95 100 101 101 103 7.8
4 16 39 79 108 106 99 108 101 103 3.9
7 18 39 80 107 103 93 106 107 103 2
5 15 41 73 114 109 101 109 107 101 0
10 5 2.5 1.2 0.6 0.3 0.2 0.1 0 0
NVP−BGT226 (nM)
E
1 1 1 1 0 0 0 0 0 2 500
2 2 2 2 1 1 1 1 1 1 250
2 7 17 14 20 15 18 16 18 14 125
3 19 30 37 60 55 60 56 59 46 62.5
3 21 29 48 85 81 91 81 85 82 31.2
5 19 35 53 101 100 98 102 100 105 15.6
3 15 37 87 98 95 96 101 100 102 7.8
5 20 39 87 100 98 96 106 100 104 3.9
5 21 38 76 93 97 93 97 98 97 2
3 22 35 78 99 94 99 98 100 100 0
10 5 2.5 1.2 0.6 0.3 0.2 0.1 0 0
NVP−BGT226 (nM)
F
2 1 1 1 1 0 1 0 1 1 500
2 3 2 3 2 1 1 1 2 2 250
2 8 19 19 24 22 23 26 22 22 125
4 13 27 36 53 54 55 55 61 51 62.5
3 15 30 43 69 71 71 73 74 74 31.2
5 13 34 50 102 108 103 108 108 104 15.6
3 12 37 74 104 108 110 110 112 100 7.8
5 17 40 67 108 103 102 113 106 107 3.9
4 13 36 52 100 111 100 101 107 109 2
4 15 36 47 103 102 114 108 108 100 0
10 5 2.5 1.2 0.6 0.3 0.2 0.1 0 0
NVP−BGT226 (nM)
NVP−AUY922 (nM)
G
1 2 5 6 6 7 7 7 5 7 10
1 3 9 19 22 21 21 19 24 19 5
1 4 15 37 35 39 35 35 35 37 2.5
1 2 18 81 83 80 84 86 98 93 1.2
0 1 19 106 107 98 99 103 100 104 0.6
0 2 14 100 103 107 101 112 108 109 0.3
0 0 29 94 103 101 105 106 101 106 0.2
0 0 5 88 105 106 100 104 102 109 0.1
0 0 1 94 105 101 101 100 98 102 0
0 0 6 88 104 101 100 102 102 107 0
10 5 2.5 1.2 0.6 0.3 0.2 0.1 0 0
NITD609 (nM)
H
0 0 3 3 6 5 4 5 2 6 10
0 1 5 9 8 10 7 7 6 7 5
−1 0 7 24 24 22 21 25 23 25 2.5
0 1 11 81 92 93 94 92 94 89 1.2
0 0 18 99 102 99 100 100 103 97 0.6
0 1 20 100 104 109 96 104 101 105 0.3
0 2 24 101 101 101 106 105 103 101 0.2
0 0 2 94 105 103 101 102 101 103 0.1
0 0 7 96 104 101 100 104 103 103 0
−1 0 5 101 101 103 97 98 104 102 0
10 5 2.5 1.2 0.6 0.3 0.2 0.1 0 0
NITD609 (nM)
I
0 0 2 2 3 6 3 3 3 2 10
0 2 6 10 9 10 9 8 9 9 5
0 2 11 20 22 21 21 21 21 22 2.5
0 1 11 24 27 26 27 30 26 27 1.2
0 0 10 81 96 89 102 100 102 95 0.6
0 1 27 108 108 108 113 114 111 105 0.3
0 0 10 99 105 106 101 106 109 105 0.2
−1 0 11 76 108 106 101 108 106 106 0.1
−1 −1 3 82 106 104 106 103 107 94 0
0 0 3 85 107 106 107 112 111 103 0
10 5 2.5 1.2 0.6 0.3 0.2 0.1 0 0
NITD609 (nM)
NVP−BGT226 (nM)
J
5 5 5 4 5 8 8 12 12 6 10
5 8 13 12 18 18 19 28 25 28 5
6 11 18 23 27 31 35 36 56 41 2.5
6 11 24 38 42 59 81 102 103 94 1.2
7 10 24 37 62 86 105 102 109 109 0.6
7 12 23 44 59 84 111 112 115 115 0.3
6 11 20 37 60 81 98 106 96 105 0.2
6 11 24 45 52 81 107 102 108 99 0.1
7 10 25 38 58 80 104 101 106 105 0
7 10 25 40 52 51 104 108 100 112 0
25 12.5 6.2 3.1 1.6 0.8 0.4 0.2 0.1 0
Atovaquone (nM)
K
4 5 7 7 9 16 15 14 12 7 10
5 7 15 20 26 32 28 32 30 27 5
7 12 23 40 41 44 51 46 51 47 2.5
11 20 39 54 66 85 100 100 101 106 1.2
9 14 37 51 75 91 108 105 107 108 0.6
9 16 33 59 73 97 118 110 110 114 0.3
8 14 24 46 69 88 106 100 98 105 0.2
8 15 27 56 66 91 103 97 100 103 0.1
8 12 30 51 68 84 103 98 105 103 0
8 13 37 42 72 84 106 101 100 106 0
25 12.5 6.2 3.1 1.6 0.8 0.4 0.2 0.1 0
Atovaquone (nM)
L
1 2 2 2 3 4 4 4 5 3 10
3 3 6 7 11 14 12 17 19 12 5
5 6 9 13 15 20 21 22 21 22 2.5
6 6 12 14 13 21 28 38 43 39 1.2
5 7 13 26 40 61 95 105 96 93 0.6
5 7 10 25 41 64 112 106 106 103 0.3
5 6 10 19 43 62 102 104 99 100 0.2
5 7 10 24 37 67 103 103 102 104 0.1
5 6 11 20 37 59 105 104 101 100 0
5 6 12 22 35 53 102 107 102 104 0
25 12.5 6.2 3.1 1.6 0.8 0.4 0.2 0.1 0
Atovaquone (nM)
NVP−BGT226 (nM)

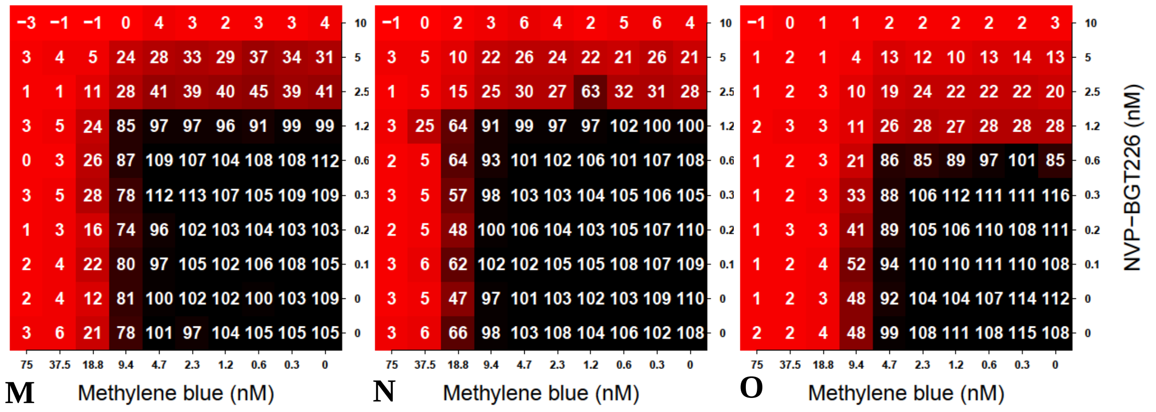

Fig. S15.1
Interaction heatmaps for NVP-BGT226 + alvespimycin against 3D7 (**A**, 1761/155), Dd2 (**B**, 1763/155) and Hb3 (**C**, 1764/155); NVP-BGT226 + NVP-AUY922 against 3D7 (**D**, 1761/69), Dd2 (**E**, 1763/69) and Hb3 (**F**, 1764/69); NVP-BGT226 + NITD609 against 3D7 (**G**, 1761/224), Dd2 (**H**, 1763/224) and Hb3 (**I**, 1764/224); NVP-BGT226 + atovaquone against 3D7 (**J**, 1761/215), Dd2 (**K**, 1763/215) and Hb3 (**L**, 1764/215); NVP-BGT226 + methylene blue against 3D7 (**M**, 1761/229), Dd2 (**N**, 1763/229) and Hb3 (**O**, 1764/229). Numbers reflect the assay ID/serial for each heatmap as listed in the malaria folder at https://tripod.nih.gov/matrix-client/.

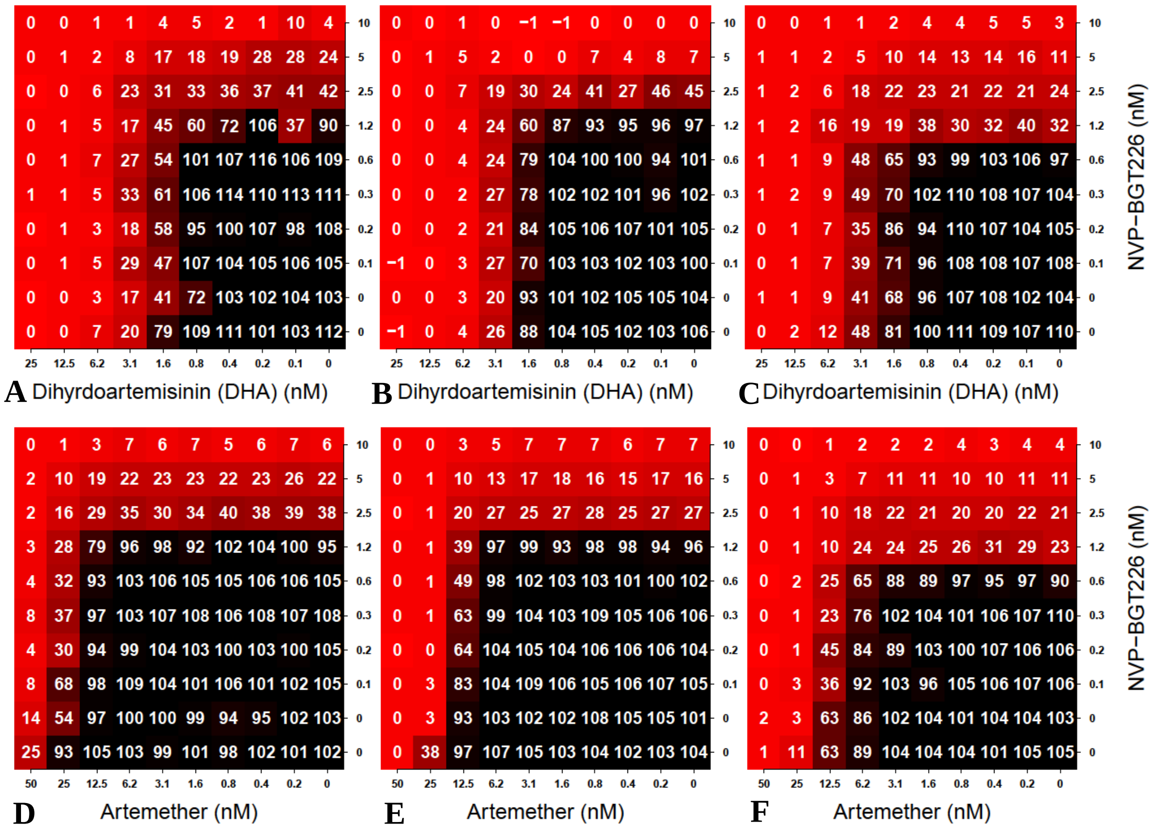

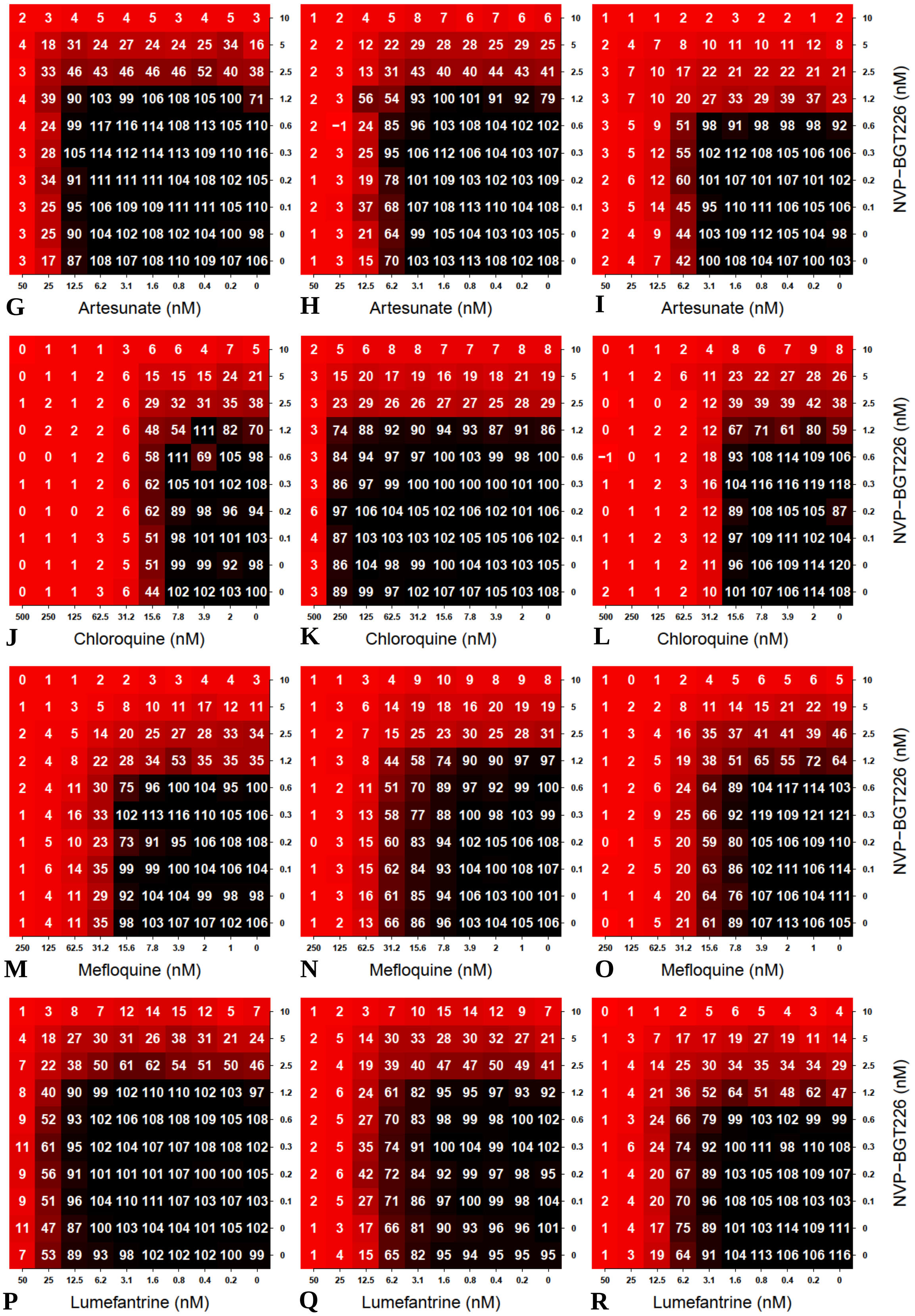
G
Artesunate (nM)
H
Artesunate (nM)
I
Artesunate (nM)
J
Chloroquine (nM)
K
Chloroquine (nM)
L
Chloroquine (nM)
M
Mefloquine (nM)
N
Mefloquine (nM)
O
Mefloquine (nM)
P
Lumefantrine (nM)
Q
Lumefantrine (nM)
R
Lumefantrine (nM)
NVP-BGT226 (nM)

Fig. S15.2

Interaction heatmaps for NVP-BGT226 + DHA against 3D7 (**A**, 1761/227), Dd2 (**B**, 1763/227) and Hb3 (**C**, 1764/227); NVP-BGT226 + ATM against 3D7 (**D**, 1761/225), Dd2 (**E**, 1763/225) and Hb3 (**F**, 1764/225); NVP-BGT226 + AS against 3D7 (**G**, 1761/226), Dd2 (**H**, 1763/226) and Hb3 (**I**, 1764/226); NVP-BGT226 + CQ against 3D7 (**J**, 1761/125), Dd2 (**K**, 1763/125) and Hb3 (**L**, 1764/125); NVP-BGT226 + MFQ against 3D7 (**M**, 1761/230), Dd2 (**N**, 1763/230) and Hb3 (**O**, 1764/230); NVP-BGT226 + LMF against 3D7 (**P**, 1761/228), Dd2 (**Q**, 1763/228) and Hb3 (**R**, 1764/228). Numbers reflect the assay ID/serial for each heatmap as listed in the malaria folder at https://tripod.nih.gov/matrix-client/.

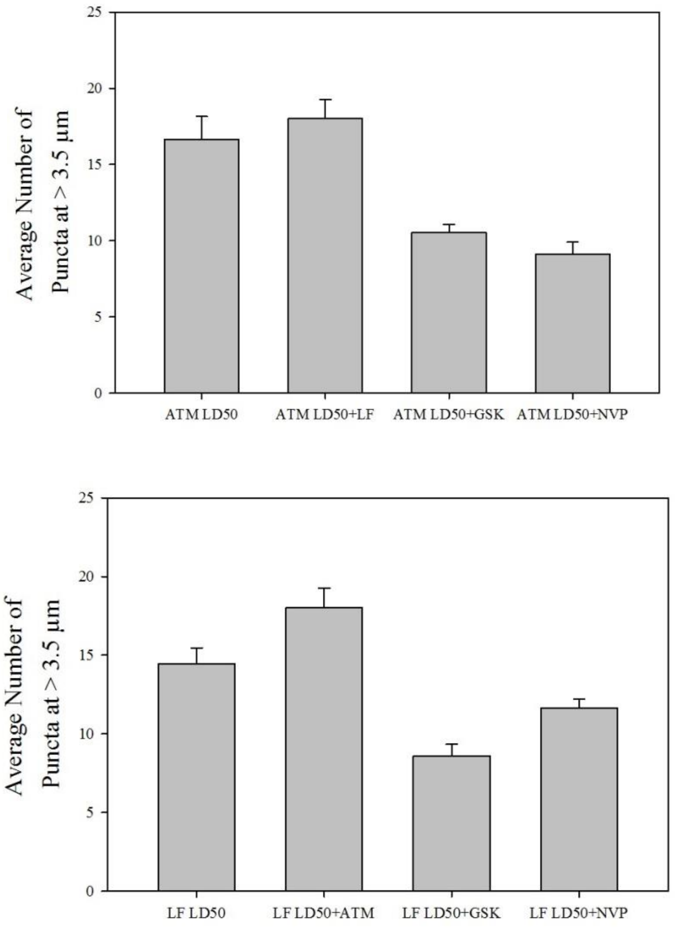

Fig. S16

PfATG8 positive puncta, quantified as described previously (*25*) were measured upon treatment with LD_{50} – doses of ATM, LF, ATM+LF (CoArtem) as well as either ATM or LF in the presence of two PI3K inhibitors (GSK2126458 or NVP226). The first two bars (left) and the last two bars (right) in each plot are not statistically different (P value > 0.05), but either of the first two bars are statistically different from either of the second two bars in both plots (P value < 0.05).

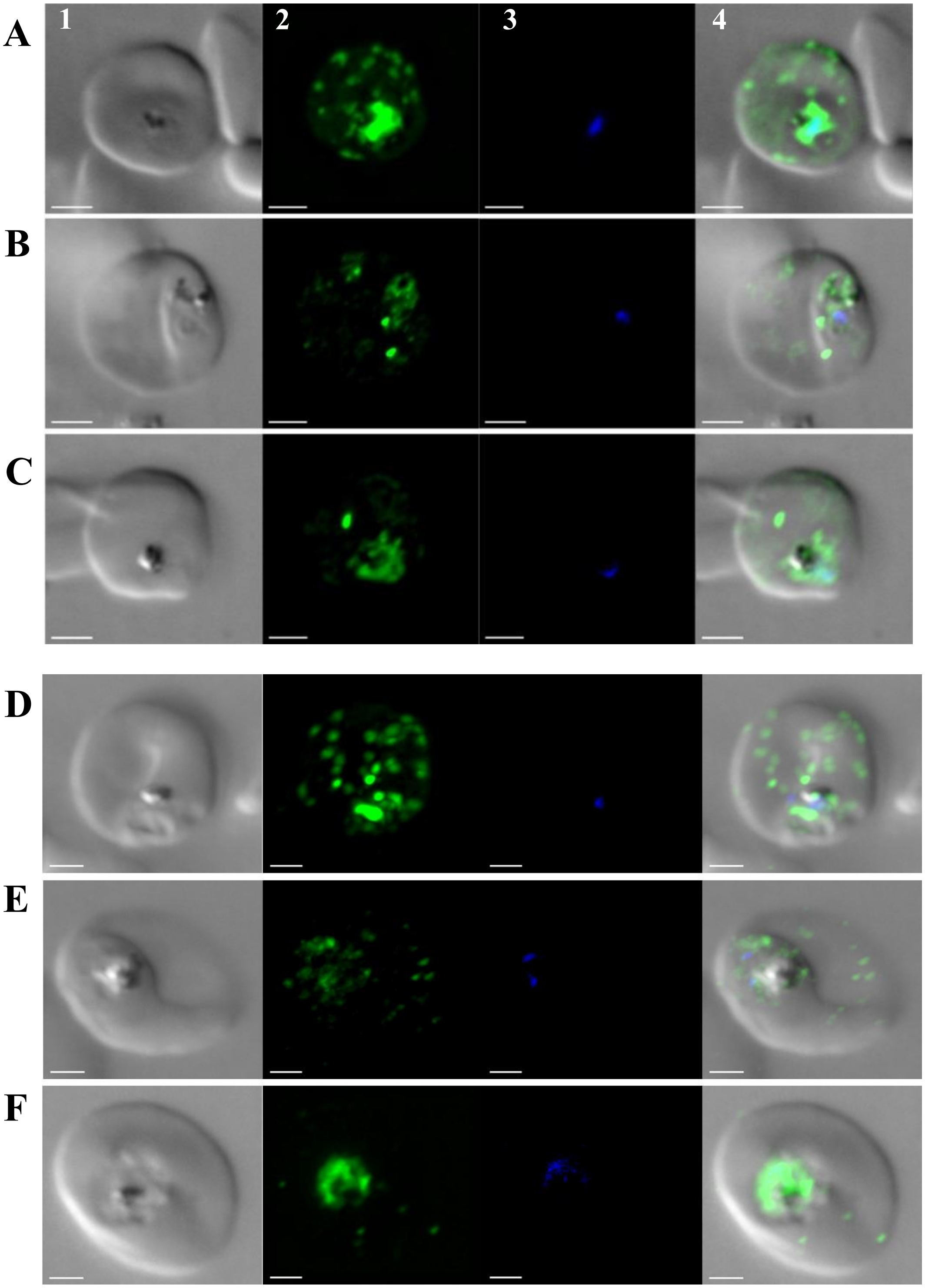
A
1
2
3
4
B
C
D
E
F

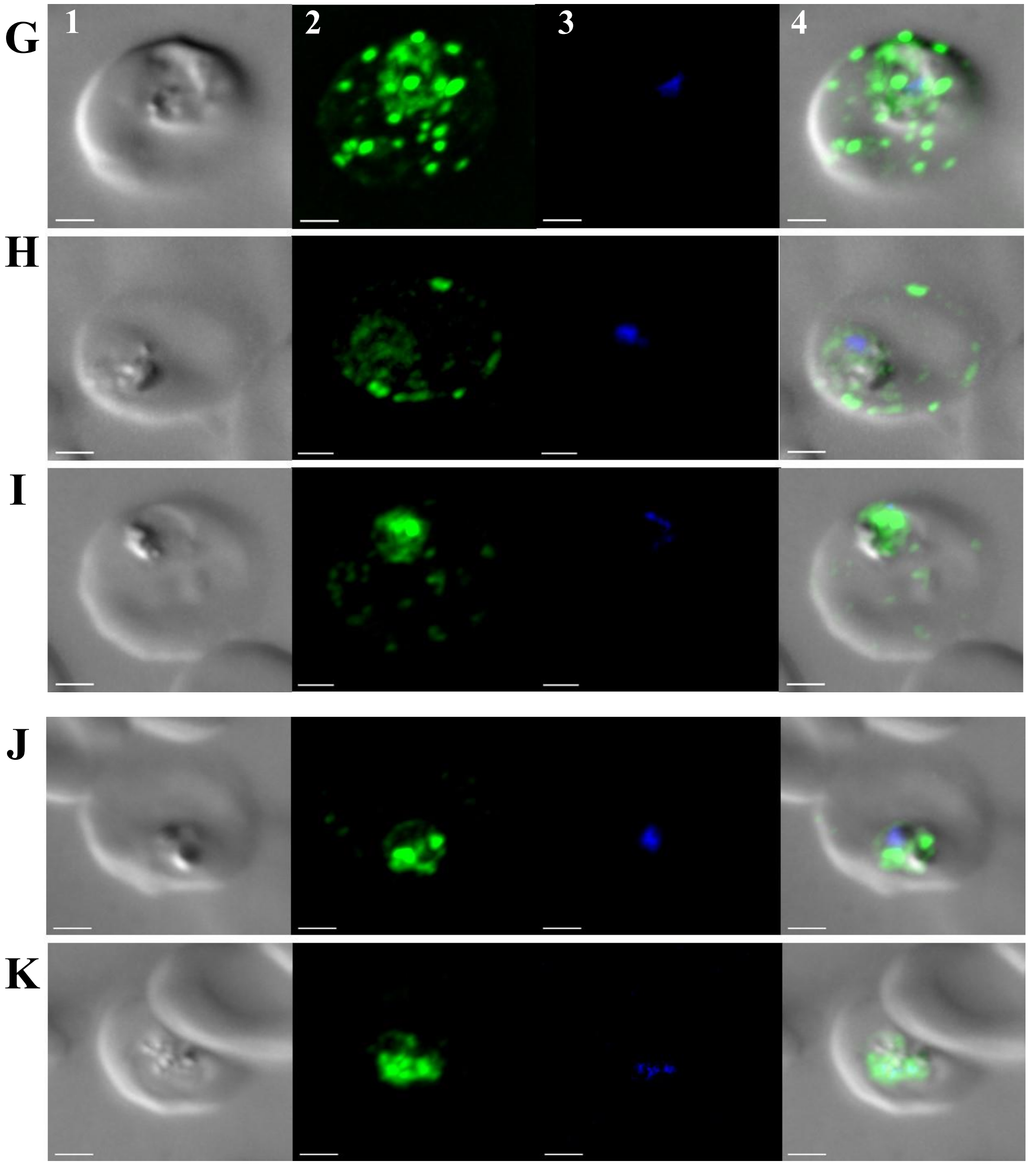

Fig. S17
Imaging and quantitation of PfAtg8 containing puncta within parasite infected RBCs as judged by a static view of RBC and parasite (panel 1), anti-PfAtg8 peptide antibody imaging (ex: 450-490, em: 500 to 550)(panel 2), DAPI nuclei staining (ex: 340-380, em: 450 to 490)(panel 3), a merged image of all three views (panel 4) of (CQS) parasites following a 6 hour exposure to (**A**) CQ at 2X the defined LD_{50} value. (**B**) CQ at 2X the defined LD_{50} value and GSK-2126458 at 102 μM. (**C**) CQ at 2X the defined LD_{50} value and NVP-BGT226 at 24 nM. (**D**) AQ at the defined LD_{50} value. (**E**) AQ at the defined LD_{50} value and GSK-2126458 at 102 μM. (**F**) LMF at the defined LD_{50} value and NVP-BGT226 at 24 nM. (**G**) AQ at the defined LD_{50} value. (**H**) LMF at the defined LD_{50} value and GSK-2126458 at 102 μM. (**I**) LMF at the defined LD_{50} value and NVP-BGT226 at 24 nM. (**J**) GSK-2126458 at the defined LD_{50} value. (**K**) NVP-BGT226 at the defined LD_{50} value. Scale bar for each image = 2 microns.

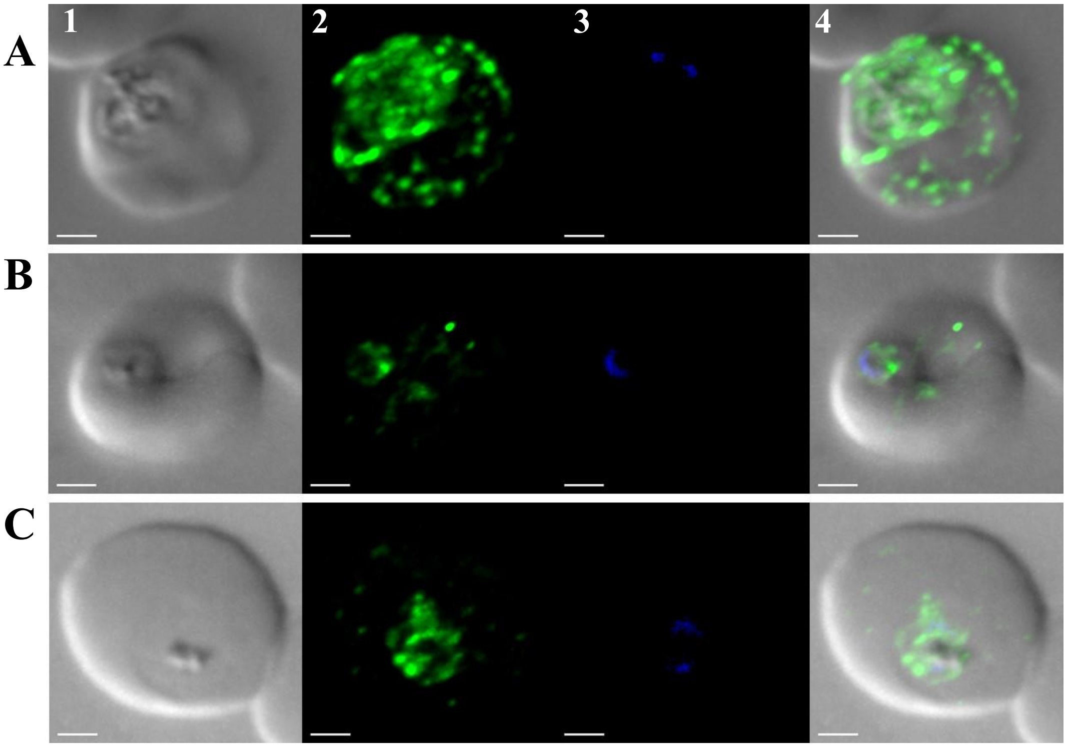

Fig. S18
Imaging and quantitation of PfAtg8 containing puncta within parasite infected RBC as judged by a static view of RBC and parasite (panel 1), anti-PfAtg8 peptide antibody imaging (ex: 450-490, em: 500 to 550)(panel 2), DAPI nuclei staining (ex: 340-380, em: 450 to 490)(panel 3), a merged image of all three views (panel 4) of (CQS) parasites following a 6 hour exposure to starvation media (**A**) with no drug added. (**B**) GSK-2126458 at the defined LD_{50} value. (**C**) NVP-BGT226 at the defined LD_{50} value. Scale bar for each image = 2 microns.

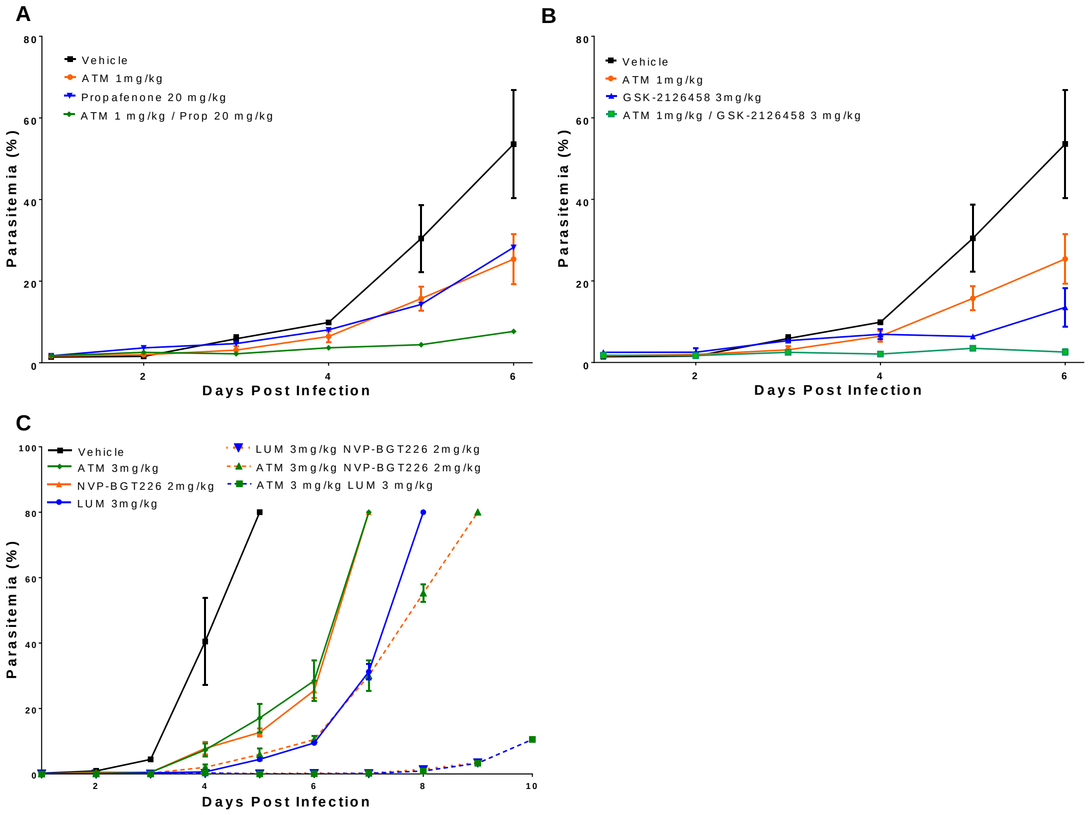

Fig. S19

A modified Peter's Suppressive Test was used to initially assess drug-drug interactions in the mouse malaria model. Female CD1, used in panels A and B, or BALB/c mice were infected i.p. with 10^6 *P. berghei* N parasites. Between two and three hours post infection drug treatment, as indicated above, was initiated by oral gavage of compound resuspended in standard suspension vehicle (0.5% hydroxyethyl cellulose and 0.1% Tween-80; Sigma, St. Louis, MO). Compound administration was continued for an additional two days (3 consecutive days of compound administration in total). Compound dosage was adjusted by weight. Parasitemias were determined by microscopic examination of Giemsa-stained blood films taken daily, beginning on day two. ATM, artemether. Note: The propafenone and GSK-2126458 experiments were performed at the same time, and used the same vehicle and ATM single agent control mice, the graphs have been separated for ease of presentation.

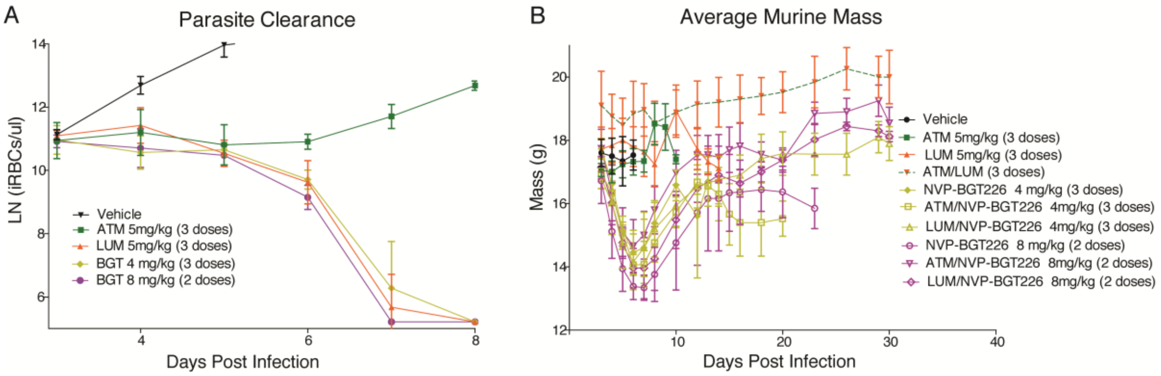

Fig. S20

A modified Thompson's 30-day Test was used to assess the curative activity of NVP-BGT226 (BGT) in combination with artemether (ATM) or lumefantrine (LUM). **A**) Single agent rate of action, compared to vehicle alone, was assessed by comparison of the natural log transformed number of infected erythrocytes (RBCs) per µl. **B**) Alteration in mouse mass associated with NVP-BGT226 treatment in addition to *P. berghei* infection. In addition to the transient drug-induced loss of weight mice also experienced lethargy, hunched posture and decreased grooming. All drug-induced effects were temporary and decreased after cessation of drug treatment.

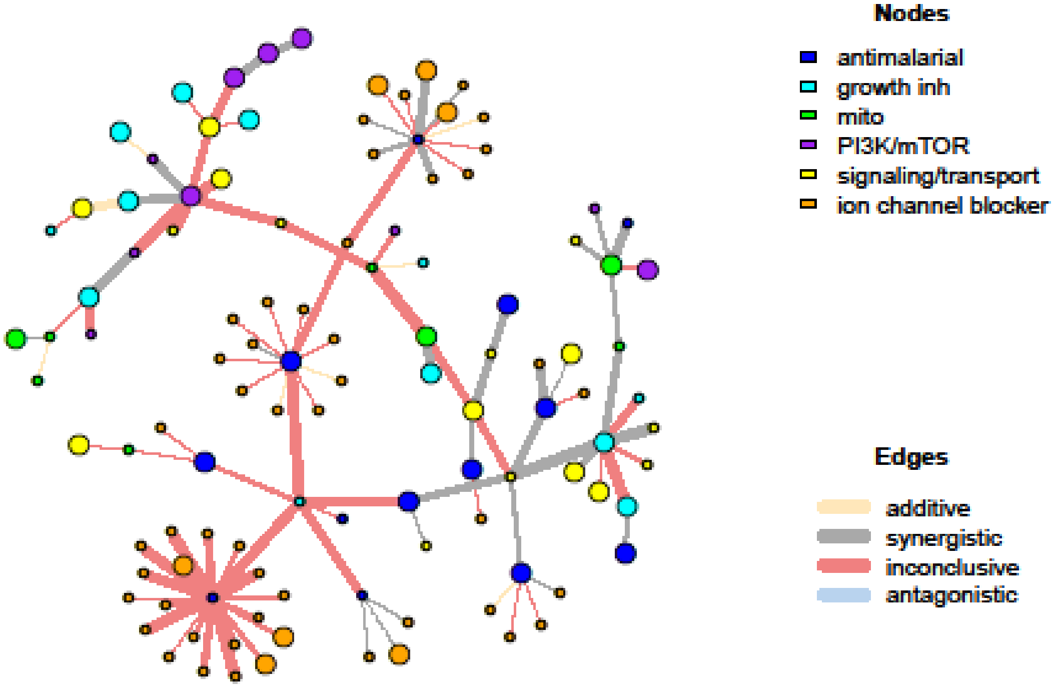

Fig. S21
The minimum spanning tree derived from the full network of 2,134 combinations, with edges colored by manually assigned combination class. This network serves to highlight that manual inspection must be performed due to the lack of robustness in the DBSumNeg metric, which may flag a combination as synergistic whereas visual inspection of the response matrix would suggest otherwise.

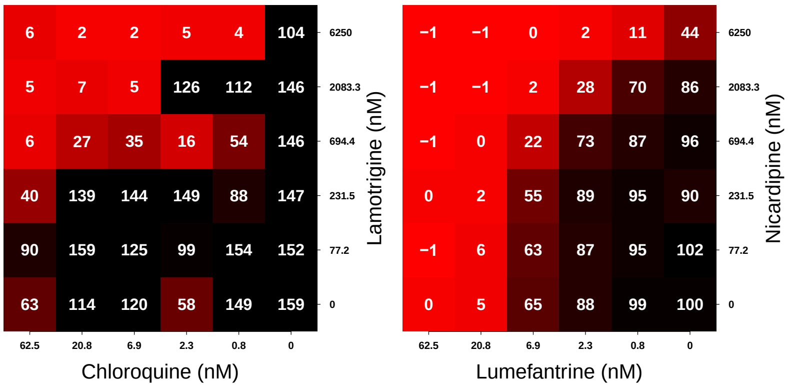

Fig. S22
Examples of combination responses identified as synergistic by DBSumNeg. Visual inspection of the responses identifies the cholorquine + lamotrigine combination (DBSumNeg = -9.66) to be a false positive, whereas the combination of lumefantrine and nicardipine does appear to be synergistic by DBSumNeg (-2.99) and visual inspection.

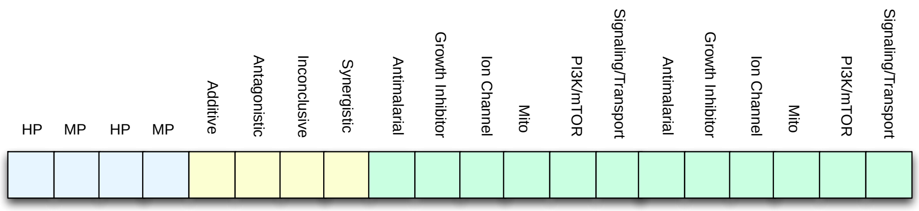

Fig. S23
The layout of the fingerprint representation used to characterize the potency, synergy and MOA of each combination.

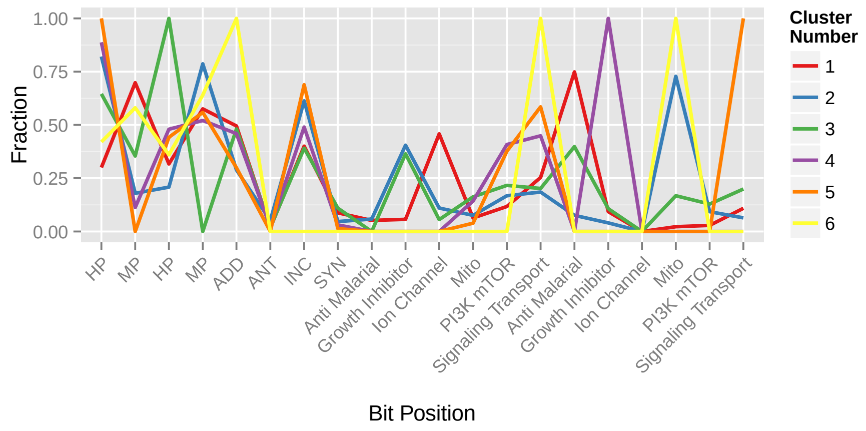

Fig. S24
A bit spectrum for each of the six clusters, highlighting distribution of parameter values within each cluster.

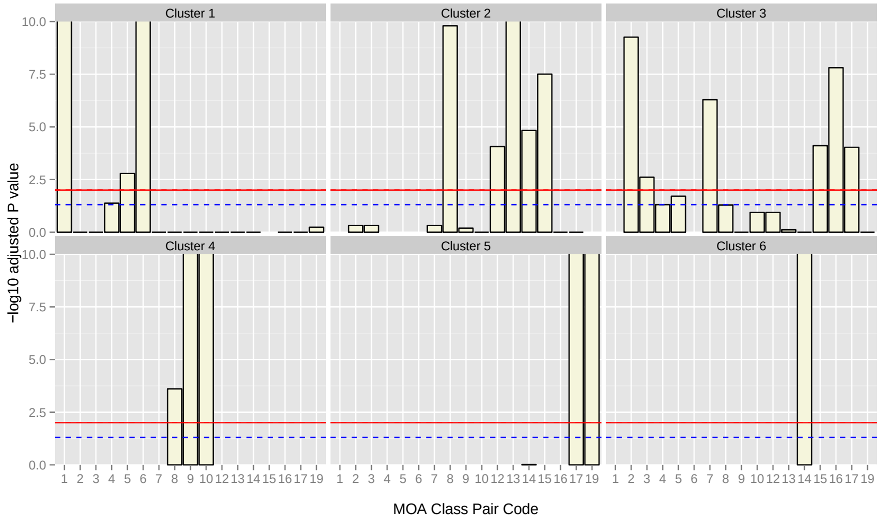

Fig. S25
A summary of the enrichment of individual MOA class pairs in each cluster. The red and blue lines correspond to p = 0.01 and p = 0.05 respectively.

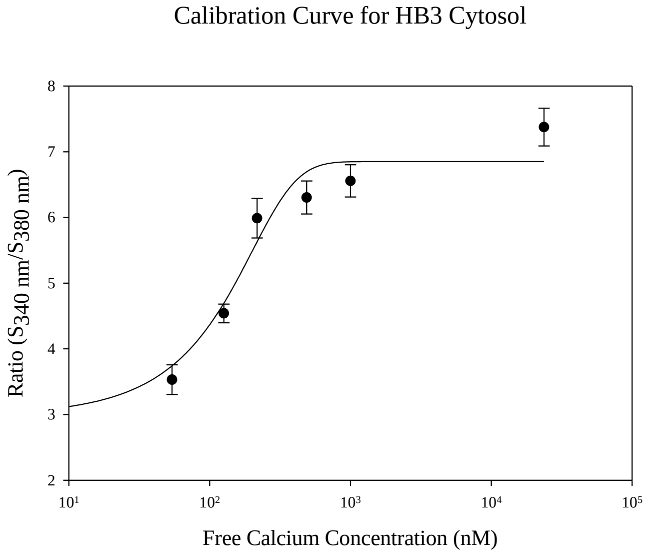

Fig. S26
Sample in situ Ca^{2+} calibration curve for HB3 cytosol loaded with Fura-2 AM. The curve was fit by a sigmoidal function. Synchronized malaria parasites loaded with Fura-2 AM at trophozoite stage were used for ratiometric imaging as described. The error bars represent standard error. For each data point, data from ≥ 15 cells perfused with calibration solution (HBSS containing 10 μM ionomycin and various CaCl2:EGTA ratios) were averaged.

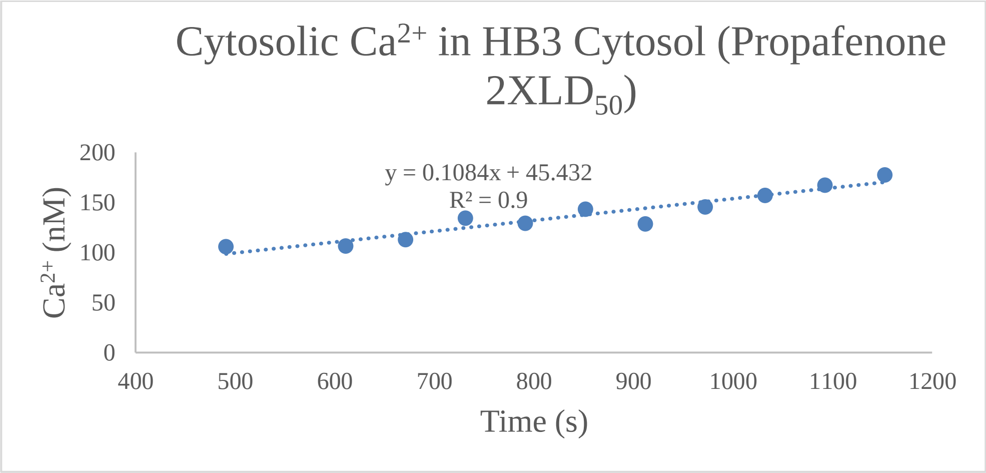

Fig. S27
Example of Ca^{2+} vs time transients for parasites under perfusion with drug. Cytosolic Ca^{2+} vs time trace for HB3 perfused with Propafenone (drug perfusate initiated at 500 seconds). The curve is fit with a linear function and the rate of change of Ca^{2+} concentration was calculated from the fit.

Supporting Tables:

Table S4 – Network Similarity Comparison for *Plasmodium falciparum* 3D7.

Compound 1	Compound 2	P value (hypergeometric test)[a]
**Artemether**	**Artesunate**	**p < 4.344e-06**
**Artemether**	**Dihydroartemisinin**	**p < 4.631e-04**
**Artemether**	**Mefloquine**	**p < 0.001**
Artemether	Halofantrine	p < 0.115
**Artemether**	**Lumefantrine**	**p < 0.007**
**Artesunate**	**Dihydroartemisinin**	**p < 3.131e-04**
**Artesunate**	**Mefloquine**	**p < 0.025**
Artesunate	Halofantrine	p < 0.163
**Artesunate**	**Lumefantrine**	**p < 0.011**
Dihydroartemisinin	Mefloquine	p < 0.158
Dihydroartemisinin	Halofantrine	p < 0.059
**Mefloquine**	**Halofantrine**	**p < 0.001**
**Mefloquine**	**Lumefantrine**	**p < 4.062e-08**
**Halofantrine**	**Lumefantrine**	**p < 0.003**

[a] Network similarity for Plasmodium falciparum 3D7 was determined based on the hypergeometric distribution probability of the overlapping drug interaction partners with a DBSumNeg < -3 from the total number of interacting matrices, based on the total number of matrix for each of these compounds (SAS, Gary, NC).

Table S5. Interacting Compounds with DBSumNeg ≤ -3 against *Plasmodium falciparum* 3D7.

Artemether	Artesunate	Dihydroartemisinin	Mefloquine	Halofantrine	Lumefantrine
Alvespimycin	**Calhex-231**	**Lumefantrine**	**Artemether**	**Artesunate**	**Artesunate**
Amodiaquine	Emetine	**Manoalide**	**Artesunate**	**CP-466722**	**Artemether**
**Calhex-231**	**Halofantrine**	**Methylene blue**	**BTM-2C-dimer allyl oxime**	**KN-62**	**BTM-2C-dimer allyl oxime**
Elactocin	**KN-62**	Nanchangmycin	**CP-466722**	**Lumefantrine**	**CP-466722**
**GSK-2126458**	**Lomerizine**	**NVP-BGT226**	**GSK-2126458**	Miconazole	Dihydroartemisinin
**KN-62**	**Lumefantrine**	**Perhexiline**	**KN-62**	Panobinostat	**Halofantrine**
**Lomerizine**	**Manidipine**	**Propafenone**	**Manidipine**	**Reserpine**	**KN-62**
**Lumefantrine**	Manoalide	**Reserpine**	**Midostaurin**		Lidoflazine
**Manidipine**	**Mefloquine**		**Nicardipine**		**Manidipine**
**Mefloquine**	**Midostaurin**		**Propafenone**		**Midostaurin**
**Methylene blue**	**Nicardipine**		**Reserpine**		**Nicardipine**
**Nicardipine**	**Perhexiline**		**Rifampicin**		**Reserpine**
NITD609	**Propafenone**				**Rifampicin**
NPS-R568	**Torin-2**				**Torin-2**
NVP-AUY922					**Zaldaride**
**NVP-BGT226**					
**Perhexiline**					
Piperaquine					
**Propafenone**					
Pyronaridine					
Quisinostat					
**Reserpine**					
**Rifampicin**					
**Torin-2**					
**Zaldaride**					

Compounds listed passed our quality control analysis (see Methods) with at least one assay producing a DBSumNeg value ≤ -3 with the compound listed in the heading. Compounds producing a DBSumNeg ≤ -3 with more than one of the indicated compounds are shown in bold, those present in more than 3 are also highlighted in yellow.

Table S7. Change in cytosolic [Ca^{2+}] for Dd2.

Drug	Change in [Ca^{2+}] (nM/min)
CQ (250 nM=2XIC$_{50}$)	0.83
CQ (32 µM=2XLD50)	5.15

Table S8. Change in DV [Ca^{2+}] for Dd2.

Drug	Change in [Ca^{2+}] (nM/min)
CQ (250 nM=2XIC$_{50}$)	-0.08
CQ (32 µM=2XLD50)	-3.82

Table S9. Change in cytosolic $[Ca^{2+}]$ for HB3.

Drug	Change in $[Ca^{2+}]$ (nM/min)
Artemether (2XIC_{50})	1.11
Artemether (2XLD_{50})	3.91
Lumefantrine (2XIC_{50})	0.07
Lumefantrine (2XLD_{50})	2.82
Propafenone (2XIC_{50})	0.35
Propafenone (2XLD_{50})	6.50
Nicardipine (2XIC_{50})	0.04
Nicardipine (2XLD_{50})	4.84
KN-62 (2XIC_{50})	0.63
KN-62 (2XLD_{50})	2.20
CQ (2XIC_{50})	0.27
CQ (2XLD_{50})	1.30
Reserpine (0.5 μM)	1.70
Reserpine (1 μM)	1.85
Reserpine (5 μM)	3.40
PIK-93 (2XIC_{50})	0.46
PIK-93 (2XLD_{50})	0.66
Cinacalcet (2XIC_{50})	0.76
Cinacalcet (2XLD_{50})	0.53
Elesclomol (2XIC_{50})	0.47
Elesclomol (2XLD_{50})	0.50
GSK-1059615 (2XIC_{50})	0.53
GSK-1059615 (2XLD_{50})	0.38

Table S10. IC_{50} and LD_{50} values for selected agents versus HB3 and Dd2 strains.

Compound	IC_{50} (nM)				LD_{50} (nM)				Reference
	**HB3**	**SEM**	**Dd2**	**SEM**	**HB3**	**SEM**	**Dd2**	**SEM**	
CQ	22.7	N/A	225.9	N/A	126.3	30	15669	2411	Paguio, M.F.; et al. *Mol Biochem Parasitol*. **2011,** *178,* 1-6.
AQ	10	N/A	27.7	N/A	37	7	51.4	4	Paguio, M.F.; et al. *Mol Biochem Parasitol*. **2011,** *178,* 1-6.
MQ	21	N/A	30.4	N/A	476.9	109	416.6	100	Paguio, M.F.; et al. *Mol Biochem Parasitol*. **2011,** *178,* 1-6.
QD	17.8	N/A	119.1	N/A	423.7	76	27238.2	3389	Paguio, M.F.; et al. *Mol Biochem Parasitol*. **2011,** *178,* 1-6.
QN	129.3	N/A	277.7	N/A	7121.4	1161	27308.1	3798	Paguio, M.F.; et al. *Mol Biochem Parasitol*. **2011,** *178,* 1-6.
eQN	6588.5	358.2	2237.6	76.4	16100	400	36300	900	Gorka, A.P.; et al. *Antimicrob. Agents Chemother*. **2013,** *57,* 365-374.
eQD	7893.6	46.8	3588.3	194.1	17600	1400	38900	900	Gorka, A.P.; et al. *Antimicrob. Agents Chemother*. **2013,** *57,* 365-374.
PQ	1990	16.6	4695	62.1	8640	50	2810	10	Gorka, A.P.; et al. *Malaria J.* **2013**, *12,* 332-?.
TQ	2189.9	18.7	2092.2	20	42700	2042.6	12100	706	Gorka, A.P.; et al. *Malaria J.* **2013**, *12,* 332-?.
MB	5.3	0.1	5.5	0.1	120.2	9.5	108	6.6	Gorka, A.P.; et al. *Malaria J.* **2013**, *12,* 332-?.
Art	22.6	0.7	21.2	2.3	80	N/A	80	N/A	NA
GSK-2126458	89	48.2	124	27	101700	20006	16100	3037	NA
Prop	1000	N/A	1000	N/A	116167	18606	24867	20067	NA
Artem	7.2	0.9	17.1	1.8	16.3	0.4	25.1	3.9	NA
Lumef	33.3	6.9	47.4	18.9	323	52	242.3	13.3	NA
NVP-BGT226	0.63	0.2	1.03	0.1	17.5	7.5	17.3	6.8	NA
Torin2	1	0.7	2.1	0.4	1875	384	2100	493	NA
PIK93	129	30.5	198	42	2250	250	2033	851	NA
INK128	23.9	N/A	69	N/A	N/A	N/A	N/A	N/A	NA
GSK615	7700	N/A	3800	N/A	N/A	N/A	N/A	N/A	NA
KN-62	2100	N/A	1300	N/A	N/A	N/A	N/A	N/A	NA

Table S11. Chou-Talalay defined FIC values for specified combinations.

Combo	Average FIC HB3				Average FIC Dd2				Average FIC_{index}				IC Assignment	
**A/B**	**A**	**SEM**	**B**	**SEM**	**A**	**SEM**	**B**	**SEM**	**HB3**	**SEM**	**Dd2**	**SEM**	**HB3**	**Dd2**
**ATM/ LF**	0.73	0.12	0.36	0.12	0.67	0.13	0.47	0.03	1.1	0.2	1.1	0.2	Add	Add
**GSK212/ LF**	0.54	0.13	0.46	0.09	0.4	0.15	0.27	0.08	1	0.2	0.67	0.2	Syn	Syn
**GSK212/ ATM**	0.35	0.12	0.67	0.22	0.52	0.15	0.54	0.16	1	0.3	1.1	0.3	Syn	Add
**NVP/ LF**	0.98	0.12	0.61	0.07	0.93	0.22	0.6	0.07	1.6	0.1	1.5	0.3	Add	Add
**NVP/ ATM**	0.67	0.08	0.89	0.11	1.05	0.2	0.97	0.18	1.6	0.2	2	0.4	Add	Add
**Torin2/ LF**	0.35	0.13	1.08	0.29	0.48	0.1	0.44	0.06	1.4	0.4	0.9	0.1	Add	Syn
**Torin2/ ATM**	0.16	0.06	1.15	0.42	0.59	0.1	0.75	0.12	1.3	0.5	1.3	0.2	Add	Add
**PIK93/ LF**	0.56	0.15	0.68	0.12	0.34	0.09	0.47	0.1	1.2	0.3	0.81	0.2	Add	Syn
**PIK93/ ATM**	0.47	0.15	1.3	0.41	0.38	0.07	0.72	0.14	1.8	0.6	1.1	0.2	Add	Add

Table S12. Chou-Talalay defined FLD values for specified combinations.

Combo	Average FLD HB3				Average FLD Dd2				Average FLD_{index}				LD Assignment	
A/B	A	SEM	B	SEM	A	SEM	B	SEM	HB3	SEM	Dd2	SEM	HB3	Dd2
ATM/ LF	0.78	0.18	0.51	0.19	0.42	0.1	0.58	0.2	1.3	0.4	1	0.3	Add	Syn
GSK212/ LF	0.1	0.02	0.09	0.02	0.15	0.06	0.2	0.08	0.18	0.04	0.35	0.1	Syn	Syn
GSK212/ ATM	0.28	0.04	0.34	0.03	0.21	0.15	0.18	0.11	0.62	0.1	0.39	0.3	Syn	Syn
NVP/ LF	1.75	0.49	1.06	0.24	1.17	0.76	1.1	0.71	2.8	0.7	2	1	Ant	Ant
NVP/ ATM	0.76	0.1	0.66	0.09	0.46	0.19	0.27	0.09	1.4	0.2	0.93	0.3	Add	Syn
Torin2/ LF	0.08	0.05	0.21	0.18	0.19	0.04	0.5	0.12	0.28	0.2	0.5	0.4	Syn	Syn
Torin2/ ATM	0.1	0.07	0.21	0.18	0.6	0.31	0.54	0.23	0.46	0.2	0.94	0.4	Syn	Syn
PIK93/ LF	0.48	0.18	0.46	0.17	0.64	0	0.82	0.01	0.9	0.3	1.5	0.03	Syn	Add
PIK93/ ATM	0.43	0.16	0.45	0.12	1.48	0.46	1	0.16	0.85	0.3	2.1	0.4	Syn	Ant

Table S13. Chou-Talalay defined FIC values for specified combinations at physiologically relevant concentration ratios.

Combo	Average FAIVC HB3				Average FAIVC Dd2				Average $FAIVC_{index}$				AIVC Assignment	
A/B	A	SEM	B	SEM	A	SEM	B	SEM	HB3	SEM	Dd2	SEM	HB3	Dd2
NVP/LF ratio 1:250	0.24	0.04	1.3	0.14	0.10	0.04	0.51	0.20	1.53	0.17	0.61	0.24	Add	Syn
NVP/LF ratio 1:500	0.14	0.02	1.3	0.20	0.05	0.01	0.45	0.11	1.44	0.22	0.49	0.11	Add	Syn
NVP/LF ratio 1:1250	0.06	0.01	1.5	0.25	0.02	0.01	0.50	0.16	1.51	0.26	0.53	0.17	Add	Syn
NVP/LF ratio 1:2500	0.02	0.01	1.4	0.24	0.01	0.00	0.48	0.16	1.41	0.25	0.49	0.16	Add	Syn
NVP/ATM ratio 1:50	0.51	0.09	2.4	0.30	0.33	0.05	1.0	0.16	2.82	0.47	1.35	0.21	Ant	Add
NVP/ATM ratio 1:100	0.29	0.06	2.6	0.50	0.14	0.01	0.85	0.07	2.88	0.56	0.99	0.08	Ant	Syn
NVP/ATM ratio 1:200	0.14	0.03	2.6	0.51	0.07	0.00	0.86	0.05	2.72	0.54	0.93	0.05	Ant	Syn
NVP/ATM ratio 1:400	0.07	0.01	2.4	0.46	0.03	0.00	0.83	0.06	2.44	0.46	0.86	0.06	Ant	Syn

Table S40. Assignment of mechanism of action (MOA) pairing codes for clustering analysis.

Compound 1	Compound 2	Value
antimalarial	antimalarial	1
antimalarial	growth inh	2
antimalarial	mito	3
antimalarial	PI3K/mTOR	4
antimalarial	signaling/transport	5
antimalarial	ion channel blocker	6
growth inh	growth inh	7
growth inh	mito	8
growth inh	PI3K/mTOR	9
growth inh	signaling/transport	10
growth inh	ion channel blocker	11
mito	mito	12
mito	PI3K/mTOR	13
mito	signaling/transport	14
mito	ion channel blocker	15
PI3K/mTOR	PI3K/mTOR	16
PI3K/mTOR	signaling/transport	17
PI3K/mTOR	ion channel blocker	18
signaling/transport	signaling/transport	19
signaling/transport	ion channel blocker	20
ion channel blocker	ion channel blocker	21

Table S41. Sample diagram of plate for Chou-Talalay method assay.

	HB3 (CQS)		Dd2 (CQR)	
	**1 2 3**	**4 5 6**	**7 8 9**	**10 11 12**
**A**	100 nM ATM/ 80 nM NVP	No drug HB3	100 nM ATM/ 80 nM NVP	No drug Dd2
**B**	50 nM ATM/ 40 nM NVP		50 nM ATM/ 40 nM NVP	
**C**	25 nM ATM/ 20 nM NVP		25 nM ATM/ 20 nM NVP	
**D**	12.5 nM ATM/ 10 nM NVP		12.5 nM ATM/ 10 nM NVP	
**E**	6.25 nM ATM/ 5 nM NVP		6.25 nM ATM/ 5 nM NVP	
**F**	3.13 nM ATM/ 2.5 nM NVP		3.13 nM ATM/ 2.5 nM NVP	
**G**	1.56 nM ATM/ 1.25 nM NVP		1.56 nM ATM/ 1.25 nM NVP	
**H**	0.78 nM ATM/ 0.63 nM NVP		0.78 nM ATM/ 0.63 nM NVP	

Supplemental References

1 Dodge, Y., Cox, D., Commenges, D., Solomon, P. J. & Wilson, S. *The Oxford dictionary of Statistical Terms*. (Oxford University Press, 2003).

2 Moran, P. A. Notes on continuous stochastic phenomena. *Biometrika*, 17-23 (1950).

3 Guha, R. & Schürer, S. C. Utilizing high throughput screening data for predictive toxicology models: protocols and application to MLSCN assays. *J. Comp. Aid. Molec. Des.* **22**, 367-384 (2008).

4 Fisher, R. A. On the interpretation of χ^2 from contingency tables, and the calculation of P. *J. R. Stat. Soc.*, 87-94 (1922).

5 Benjamini, Y. & Hochberg, Y. Controlling the false discovery rate: a practical and powerful approach to multiple testing. *J. R. Stat. Soc.*, 289-300 (1995).

6 Team, R. C. R: A language and environment for statistical computing. (2012).

7 Bennett, T. N. *et al.* Novel, rapid, and inexpensive cell-based quantification of antimalarial drug efficacy. *Antimicrob. Agents Chemother.* **48**, 1807-1810 (2004).

8 Klonis, N. *et al.* Altered temporal response of malaria parasites determines differential sensitivity to artemisinin. *Proc. Nat. Acad. Sci.* **110**, 5157-5162 (2013).

9 Desjardins, R. E., Canfield, C., Haynes, J. & Chulay, J. Quantitative assessment of antimalarial activity in vitro by a semiautomated microdilution technique. *Antimicrob. Agents Chemother.* **16**, 710-718 (1979).

10 Chou, T.-C. & Talalay, P. Quantitative analysis of dose-effect relationships: the combined effects of multiple drugs or enzyme inhibitors. *Adv. Enz. Regul.* **22**, 27-55 (1984).
11 Suberu, J. O. *et al.* Anti-plasmodial polyvalent interactions in *Artemisia annua* L. aqueous extract–possible synergistic and resistance mechanisms. *PLoS One* **8**, e80790 (2013).
12 Bell, A. Antimalarial drug synergism and antagonism: mechanistic and clinical significance. *FEMS Microbiol. Lett.* **253**, 171-184 (2005).
13 Griner, L. A. M. *et al.* High-throughput combinatorial screening identifies drugs that cooperate with ibrutinib to kill activated B-cell–like diffuse large B-cell lymphoma cells. *Proc. Nat. Acad. Sci.* **111**, 2349-2354 (2014).
14 Ali, S., Najmi, M. H., Tarning, J. & Lindegardh, N. Pharmacokinetics of artemether and dihydroartemisinin in healthy Pakistani male volunteers treated with artemether-lumefantrine. *Malar. J.* **9**, 275 (2010).
15 Pawluk, S. A., Wilby, K. J. & Ensom, M. H. Pharmacokinetic profile of artemisinin derivatives and companion drugs used in artemisinin-based combination therapies for the treatment of *Plasmodium falciparum* malaria in children. *Clin. Pharmacokinet.* **52**, 153-167 (2013).
16 Markman, B. *et al.* Phase I safety, pharmacokinetic, and pharmacodynamic study of the oral phosphatidylinositol-3-kinase and mTOR inhibitor BGT226 in patients with advanced solid tumors. *Ann. Oncol.* **23**, 2399-2408 (2012).
17 Rottmann, M. *et al.* Spiroindolones, a potent compound class for the treatment of malaria. *Science* **329**, 1175-1180 (2010).
